# Field- and temperature-dependent quantum tunnelling of the magnetisation in a large barrier single-molecule magnet

**DOI:** 10.1038/s41467-018-05587-6

**Published:** 2018-08-07

**Authors:** You-Song Ding, Ke-Xin Yu, Daniel Reta, Fabrizio Ortu, Richard E. P. Winpenny, Yan-Zhen Zheng, Nicholas F. Chilton

**Affiliations:** 1 Frontier Institute of Science and Technology (FIST), State Key Laboratory of Mechanical Behavior for Materials, MOE Key Laboratory for Nonequilibrium Synthesis of Condensed Matter and School of Science, Xi’an Jiaotong University, 99 Yanxiang Road, 710054 Xi’an, Shaanxi China; 2 School of Chemistry, The University of Manchester, Oxford Road, Manchester, M13 9PL UK

## Abstract

Understanding quantum tunnelling of the magnetisation (QTM) in single-molecule magnets (SMMs) is crucial for improving performance and achieving molecule-based information storage above liquid nitrogen temperatures. Here, through a field- and temperature-dependent study of the magnetisation dynamics of [Dy(^t^BuO)Cl(THF)_5_][BPh_4_]·2THF, we elucidate the different relaxation processes: field-independent Orbach and Raman mechanisms dominate at high temperatures, a single-phonon direct process dominates at low temperatures and fields >1 kOe, and a field- and temperature-dependent QTM process operates near zero field. Accounting for the exponential temperature dependence of the phonon collision rate in the QTM process, we model the magnetisation dynamics over 11 orders of magnitude and find a QTM tunnelling gap on the order of 10^−4^ to 10^−5^ cm^−1^. We show that removal of Dy nuclear spins does not suppress QTM, and argue that while internal dipolar fields and hyperfine coupling support QTM, it is the dynamic crystal field that drives efficient QTM.

## Abstract (web summary)

Understanding quantum tunnelling of the magnetisation in single-molecule magnets is crucial for their potential application in information storage. Here the authors conduct a field- and temperature-dependent study of the magnetisation dynamics of a dysprosium-based SMM, finding four distinct relaxation processes that dominate in different regimes.

## Introduction

Individual molecules are attractive candidates as bits for magnetic data storage because they are orders of magnitude smaller than current technologies, cheap to make, monodisperse, reproducible, and solution processable. Single-molecule magnets (SMMs) are paramagnetic molecules with large magnetic anisotropy that generates an energy barrier to magnetic relaxation, *U*_eff_, resulting in slow relaxation of magnetisation and magnetic hysteresis^1,2^; aside from data storage applications, SMMs have great potential in quantum computing as generalised qubits (known as qudits)^3^, and have recently starred in the demonstration of Grover’s quantum search algorithm^4,5^. For SMMs to be economically viable in magnetic data storage, hysteresis should be observable at temperatures greater than that of liquid nitrogen (77 K)^2^; however, it has become clear that a large *U*_eff_ barrier is not the sole requirement for this to be achieved^6,7^. For example, a dysprosocenium SMM that exhibits hysteresis at 60 K has *U*_eff_ = 1760 K (see ref. ^2^), while [Dy(^t^BuO)_2_(pyridine)_5_][BPh_4_] has a higher *U*_eff_ = 1815 K, but only shows open hysteresis at 3 K (see ref. ^6^). Detailed studies are needed to understand this discrepancy.

SMM behaviour is governed by two factors: the static electronic structure and the dynamic coupling of the molecule to its environment. The static electronic structure must possess a bistable ground state with a large magnetic moment, and well-separated excited states with magnetic moments co-linear with the ground state. Remarkable *U*_eff_ values^6–13^ have been achieved for monometallic terbium(III) and dysprosium(III) complexes with strong axial ligand fields^14–16^. The dynamic coupling of the molecule to its environment, known as spin-phonon coupling, allows exchange of energy and angular momentum and effects magnetic relaxation^17,18^. The *U*_eff_ barrier arises from sequential Direct spin-phonon transitions (collectively known as the Orbach process), resulting in an exponential temperature dependence of the relaxation rate (i.e., $\tau ^{ - 1} \propto {\mathrm e}^{ - U_{\mathrm{eff}}/T}$)^18^. However, many observations of lanthanide SMMs are inconsistent with solely Orbach relaxation, and other processes such as two-phonon Raman relaxation, quantum tunnelling of magnetisation (QTM) and Direct single-phonon relaxation between the ground state (pseudo-)doublet must be operational^7,19–21^. Indeed, QTM can manifest when SMMs are adsorbed to surfaces (a prerequisite of making devices with SMMs)^22^, and thus understanding this process is crucial for commercialising SMM research; recent studies have highlighted the importance of extrinsic effects such as conduction electrons^23^ and the spin-phonon coupling^24^ on QTM.

For the most recent lanthanide-based SMMs, *U*_eff_ is >1000 K and the Orbach relaxation process is irrelevant to the low temperature magnetisation dynamics^6–13^. Therefore, investigation of large-*U*_eff_ systems at the lowest temperatures permits study of the alternative relaxation mechanisms. Our present understanding of spin-phonon relaxation is largely derived from studies of simple inorganic salts^17^, assuming Debye-like phonon spectra and ignoring optical phonon modes. This has provided expressions for the field and temperature dependencies of different relaxation processes along with approximate parameter ranges, but it is not obvious that these assumptions will apply to new and exotic molecular species; such shortcomings have been echoed recently by others^25,26^. For example, the low temperature relaxation rate for dysprosocenium follows a *T*^2^ two-phonon Raman process^2^, where the exponent of 2 is much reduced from the traditionally expected 7–9 for a Kramers ion^17^; recent studies suggest this is due to the nature of the Cp^ttt^ ligand^27^, where the electronic structure of the dysprosium(III) ion is not dictated by any single donor atom but rather by the π-electron system.

Therefore, we have made a concerted effort to determine experimentally the magnetisation dynamics of a dysprosium(III) SMM with large *U*_eff_, viz [Dy(^t^BuO)Cl(THF)_5_][BPh_4_]·2THF (**1**), where *U*_eff_ is ~1000 K. We have employed both alternating and direct current (AC and DC) magnetic measurements to examine the temperature and field dependence of magnetic relaxation. Studies on pure **1** and two solid-state dilute analogues ~12%Dy@**2** (**1a**) and ~3%Dy@**2** (**1b**) (where **2** is the isostructural diamagnetic complex [Y(^t^BuO)Cl(THF)_5_][BPh_4_]·2THF) allows us to assess the role of internal dipolar fields in the relaxation dynamics. Compound **1** was chosen, in preference to dysprosocenium^2^, as relaxation can be measured in a reasonable timescale over a wide temperature range, and because **1** exhibits QTM at low temperatures in addition to thermally driven processes.

## Results and Discussion

### Synthesis and structure

**Fig. 1:**
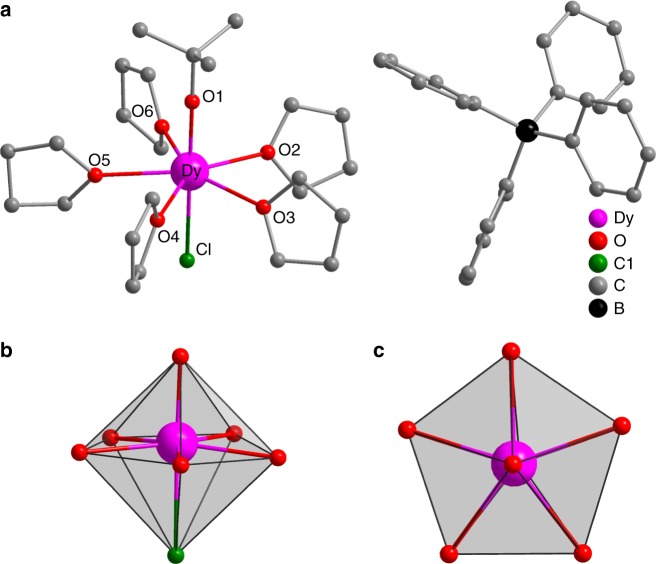
Molecular structure. **a** X-ray structure of **1**; hydrogen atoms and solvent molecules are omitted for clarity. **b** Side view of coordination polyhedron of **1**. **c** Top view of coordination polyhedron of **1**. Dysprosium (magenta), oxygen (red), chlorine (green), carbon (grey) and boron (black)

[Dy(^t^BuO)Cl(THF)_5_][BPh_4_]·2THF (**1**) was prepared based on a modification of published protocols^28^, by reaction of anhydrous DyCl_3_ with one equivalent of both NaO^t^Bu and NaBPh_4_ in dry THF, followed by crystallisation by layering with hexane (see Methods). The dysprosium(III) ion is found to be seven coordinate at the centre of a pentagonal bipyramid with pseudo-C_5v_ symmetry (Fig. 1a; Supplementary Table 1). The Dy–O(^t^BuO), Dy–Cl and Dy-O(THF) bond lengths are 2.043(4), 2.6619(12), and 2.390(3)–2.426(3) Å, respectively. The Cl–Dy–O (^t^BuO) angle is 178.26(9)°, displaying an essentially linear coordination of the negatively charged donor atoms. The nearest-neighbour equatorial O(THF)–Dy–O(THF) angles lie between 71° and 73° (72° for ideal C_5v_), highlighting the high pseudo-symmetry of **1** (Supplementary Table 2). The shortest intermolecular Dy···Dy distance is 8.54 Å. The isostructural yttrium(III) analogue [Y(^t^BuO)Cl(THF)_5_][BPh_4_]·2THF (**2**) has very similar metric parameters (Supplementary Table 1); we have also prepared two magnetically dilute compounds: ~12% Dy@**2** (**1a**) and ~3% Dy@**2** (**1b**).

### Electronic structure

The static electronic structure of **1** was determined by complete active space self-consistent field spin-orbit (CASSCF-SO) calculations with MOLCAS 8.0 (see Methods)^29^. The electronic structure is dominated by the strong axial potential of the trans-disposed ^t^BuO^−^ and Cl^−^ ligands, leading to the lowest two Kramers doublets having well defined projections of the total angular momentum (|±15/2> and |±13/2>, respectively) along the main O-Dy-Cl axis, and large energy splittings between the excited states (Supplementary Table 3). These calculations suggest that **1** will be an SMM and that Orbach relaxation will be seen via the 3rd and/or 4th Kramers doublets, which are strongly mixed, giving *U*_eff_ ~ 850 K.

### Magnetic measurements

**Fig. 2:**
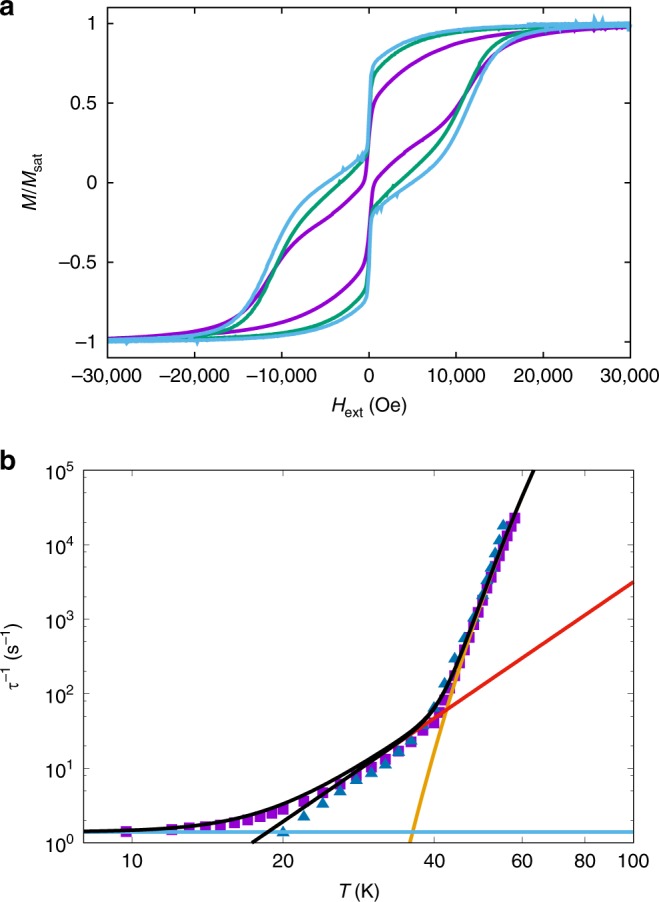
Magnetic hysteresis and relaxation rate. **a** Magnetic hysteresis for **1** (purple), ~9% Dy@**2** (green, similar concentration to **1a**) and ~5% Dy@**2** (light blue, similar concentration to **1b**) at 2 K with a sweep rate of ~50 Oe s^−1^. **b** Magnetic relaxation rate for **1** (purple squares) and **1a** (blue triangles) measured by AC magnetometry (note the log-log scale); black line is a fit with Eq. (1) using the parameters: *U*_eff_ = 950(20) K, *τ*_0_ = 3(1) × 10^−12^ s, *C* = 2(1) × 10^−6^ s^−1^ K^−*n*^, *n* = 4.6(2), *τ*^−1^_QTM_ = 1.4(1) s^−1^ ($\tau _{\mathrm{QTM}}^{ - 1} = 0$ for **1a**) and *D* = 0 (fixed), orange line is the Orbach component alone, red line is the Raman component alone, blue line is the QTM component alone (for **1**)

The DC magnetic properties for a restrained polycrystalline sample of **1** are as expected for a monometallic dysprosium(III) complex with large magnetic anisotropy (Supplementary Figs. 1 and 2). The magnetisation exhibits hysteresis at low temperatures with a waist-restricted shape (Fig. 2a; Supplementary Fig. 3) and AC magnetic studies in zero DC field show frequency-dependent out-of-phase signals (Supplementary Figs. 4 and 5); therefore **1** is an SMM. AC studies in zero DC field for the ~12% dilute sample **1a** also show frequency-dependent out-of-phase signals (Supplementary Figs. 6 and 7); the most dilute sample **1b** is too weak to measure with AC magnetometry. Fitting the AC data with the generalised Debye model^18^ yields the temperature-dependent relaxation rates *τ*^−1^ (Supplementary Tables 4 and 5). The relaxation rate has an exponential temperature dependence above 40 K, curves with decreasing temperature, and approaches a temperature-independent regime below 16 K for **1** (Fig. 2b). The AC relaxation rates for **1a** are practically superimposable with those for **1** above 30 K (Fig. 2b), however we do not observe these data transitioning towards a temperature independent regime like that observed for **1**; we expect that this would occur at lower temperatures, however these timescales are outside the range of our AC instrumentation.

**Fig. 3:**
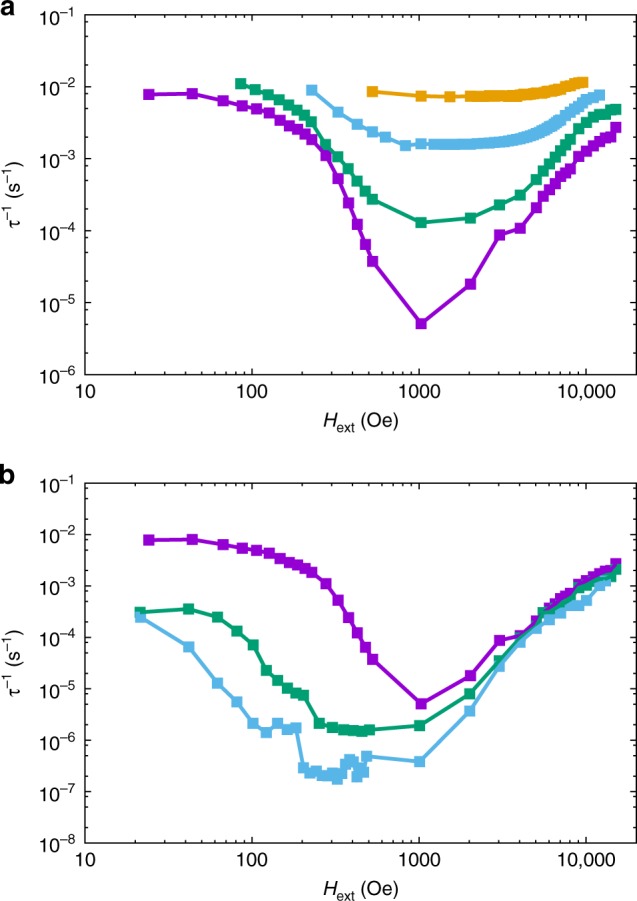
Field dependence of magnetic relaxation rate. **a** Magnetic relaxation rate for **1** (note log-log scale) at 2 (purple), 4 (green), 6 (light blue) and 8 K (orange; see Supplementary Fig. 10 for 3, 5, 7, and 9 K data). **b** Magnetic relaxation rate for **1** (purple), **1a** (green) and **1b** (light blue; note log-log scale) at 2 K. Solid lines are a guide for the eye. Error bars are within the data points

The hysteresis data for **1** indicate efficient relaxation at low temperatures and zero field, and while experiments on dilute samples show that the zero field relaxation is slightly slower than for pure **1**, it is still a significant effect. To understand the origin of this enhanced relaxation, we would like to measure the relaxation rate at lower temperatures, however, with our instrumentation AC techniques are not applicable for *τ*^−1^ < 1 s^−1^ (*T* < 10 K for **1** and < 20 K for **1a**), and DC decay techniques are not reliable for *τ*^−1^ > 0.01 s^−1^. Therefore, we adopted a different approach: the sample was magnetised at low temperature (2 K ≤ *T* ≤ 9 K) and high field (*H*_*i*_ = 50 kOe), the field was then reduced to a smaller value (10 Oe ≤ *H*_ext_ ≤ 15 kOe) and the magnetisation decay to equilibrium was measured under fixed DC field (Supplementary Fig. 8). Fitting the decay curves with stretched exponentials (see Methods) yields *τ*^−1^ and the equilibrium magnetisation *M*_eq_ (Fig. 3; Supplementary Figs. 9–12; Supplementary Tables 6–22)^18^; note that the lowest field at which *τ*^−1^ can be measured is temperature dependent.

The relaxation rate for **1** is fast near zero-field but is slowed by orders of magnitude in small fields at low temperatures. At 2–4 K the field dependence is weak for *H*_ext_ < 200 Oe and stronger for 300 Oe ≤ *H*_ext_ ≤ 600 Oe (Fig. 3; Supplementary Figs. 8–12). As the field is increased further (> 1 kOe), the relaxation rate increases again. At higher temperatures, e.g., 8 K, the field dependence of the relaxation rate becomes far less pronounced (Fig. 3). Data for analogous experiments on the ~12% doped sample **1a** show similar features (Fig. 3b; Supplementary Figs. 11 and 12) to **1**, including being coincident for *H*_ext_ > 2 kOe, however the low-field relaxation rates are almost two orders of magnitude slower than those for **1**, and the transition from weak to strong field dependence happens at a smaller field (*H*_ext_ ~ 60 Oe). Data for the most dilute sample **1b** (~3%) is only reliable for *T* = 2 K (Fig. 3b) and show four notable features: (i) the low-field relaxation rates are further slowed compared to **1** and **1a**, (ii) the high-field data (>2 kOe) are nearly coincident with those of **1** and **1a**, (iii) the field dependence for *H*_ext_ < 300 Oe has a similar gradient (on a log–log plot) as in the strongly field-dependent regimes for **1** and **1a**, and (iv) there is no weakly field dependent regime at the lowest fields.

### AC studies to obtain temperature dependence of the relaxation rate

The temperature dependence of the relaxation rate from AC experiments shows three distinct regimes for pure **1** (Fig. 2b), a feature observed previously in dysprosium(III) SMMs^21^. Above 40 K there is an exponential dependence on *T*, between 40 and ca. 20 K the *T* dependence of the relaxation rate curves, and below ca. 14 K the relaxation rate is almost independent of *T*. To fit these data, we use Eq. (1).

1 $$\tau ^{ - 1} = \tau _0^{ - 1}{\mathrm e}^{ - U_{\mathrm{eff}}/T} + {\mathrm{CT}}^n + \tau _{\mathrm{QTM}}^{ - 1} + {\mathrm{DH}}^mT^l$$

Above 40 K the exponential profile suggests that relaxation occurs via an Orbach mechanism over a large *U*_eff_ barrier; this region is fitted by the first term in Eq. (1). Between 40 and 20 K the curving profile of the relaxation rate suggests the onset of a two-phonon Raman process, described by the second term in Eq. (1). The near temperature-independent regime for **1** is ascribed to QTM (accounted for by the third term in Eq. (1)), a feature that is not observed on the timescale of our AC experiments for **1a**, suggesting that the QTM for **1** at least partially owes to internal dipolar fields. The fourth term is included to allow for the field dependence of relaxation (see below) and is omitted for now. Fitting the AC data for **1** and **1a** simultaneously (assuming *τ*^−1^_QTM_ = 0 for **1a**) with Eq. (1) gives (values in parentheses are the standard errors associated with each parameter): *U*_eff_ = 950(20) K, *τ*_0_ = 3(1) × 10^−12^ s, *C* = 2(1) × 10^−6^ s^−1^ K^−*n*^, *n* = 4.6(2) and *τ*^−1^_QTM_ = 1.4(1) s^−1^ (Fig. 2b). Notably, the data for **1** cannot be modelled without a temperature-independent term, nor without the Raman term; therefore, there must be at least three relaxation mechanisms operational in zero field.

### DC studies to obtain field dependence of the relaxation rate

**Fig. 4:**
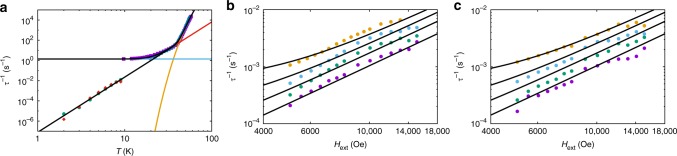
Low temperature and high field magnetic relaxation. **a** Magnetic relaxation rate measured by AC magnetometry for **1** (purple squares) and **1a** (blue triangles) and DC magnetometry for **1** (green circles) and **1a** (red diamonds; note the log-log scale); DC points chosen as the slowest relaxation rates at each temperature for each sample. Black lines are a fit with Eq. (1) using the parameters: *U*_eff_ = 950(20) K, *τ*_0_ = 3(1) × 10^−12^ s, *C* = 7.8(8) × 10^−8^ s^−1^ K^−*n*^, *n* = 5.46(4), *τ*^−1^_QTM_ = 1.4(1) s^−1^ (*τ*^−1^_QTM_ = 0 for AC data of **1a** and all DC data) and *D* = 0 (fixed), orange line is the Orbach component alone, red line is the Raman component alone, blue line is the QTM component alone (only applicable for AC data of **1**). Note the two different relaxation rates for **1** at ~10 K: the AC data are collected in zero DC field where QTM is operable (*τ*^−1^_QTM_ ≠ 0) while for the DC data we assume QTM to be effectively quenched (*τ*^−1^_QTM_ = 0). **b**, **c** Magnetic relaxation rate measured by DC magnetometry (note the log–log scale) for **1** (**b**) and for **1a** (**c**) at 2 (purple circles), 3 (green circles), 4 (light blue circles) and 5 K (orange circles). Black lines are fits with Eq. (1) using the parameters given for Fig. 5a, however with *D* = 7(3) × 10^−13^ s^−1^ Oe^−*m*^ K^−*l*^, *m* = 2.20(5) and *l* = 1.23(5). Error bars for the DC data are within the data points

To probe the nature of the low temperature relaxation dynamics and QTM, we have measured the field and temperature dependence of the relaxation rate with DC decay studies. The field dependence of the relaxation rates for **1** and **1a** (Fig. 3a; Supplementary Figs. 9–12) are similar to profiles observed recently for vanadyl complexes^30–32^. In those cases, the field dependence of magnetic relaxation was ascribed to a combination of a field-dependent Raman process at low fields, originally described by van Vleck^33^, and to the single-phonon Direct process at high fields^17^. However, here we observe a transition to a temperature-independent regime for *T* < 14 K at *H*_ext_ = 0 in the AC data for **1**, which cannot be a Raman process; this leaves QTM as the only plausible mechanism to explain the field dependence of the relaxation rate for **1** at low temperatures and low fields (*T* < 10 K, *H*_ext_ < 1 kOe). Indeed, taking the experimental data points of the slowest relaxation rate at each temperature from the DC data for **1** and **1a** and extending the temperature-dependent relaxation plot to lower temperature (n.b. these data points are in non-zero fields and we assume that QTM has been effectively quenched in the relevant field), we clearly observe the characteristic power-law profile of a Raman relaxation process that agrees well with the zero-field AC data for **1** and **1a** (i.e., *τ*^−1^ ∝ *T*^*n*^, linear on a log–log plot, Fig. 4a). Because these data points are all at different fields and yet show an excellent *τ*^−1^ ∝ *T*^*n*^ correlation for both **1** and **1a**, we do not believe there is significant field dependence to the Raman process. Our observations are in agreement with the recent derivation of Ho and Chibotaru^34^, who show that the Raman relaxation rate for Kramers ions is independent of the magnetic field strength. Therefore, unlike the conclusions drawn for vanadyl complexes in refs. ^30–32^, we contend that the field dependence at low temperatures and low fields arises from QTM; that there is a difference between *S* = 1/2 vanadyl complexes and highly anisotropic *J* = 15/2 dysprosium(III) complexes is not unexpected. Finally, our high field data (*H*_ext_ ≥ 5 kOe) show a power-law dependence on magnetic field (i.e., *τ*^−1^ ∝ *H*^*m*^, linear on a log–log plot, Fig. 4b) that is characteristic of a Direct single-phonon relaxation process between the two states of the ground doublet, also seen in refs. ^30–32^; this process is accounted for by the fourth term in Eq. (1) (see ref.^17^).

To model the field-dependent data, we note that the Orbach mechanism (first term in Eq. (1)) has an exponential dependence on *T* and is therefore insignificant for these data where *T* < 10 K. However, the Raman term has only been modelled with data above 10 K and therefore must be refined: fitting the data in Fig. 4a for both **1** and **1a** simultaneously (*U*_eff_, *τ*_0_ and *τ*^−1^_QTM_ are fixed from above), assuming that QTM is effectively quenched under the relevant fields (i.e., *τ*^−1^_QTM_ = 0 for the DC data), we obtain *C* = 7.8(8) × 10^−8^ s^−1^ K^−*n*^ and *n* = 5.46(4) (Fig. 4a). [We note that there is a significant change in the Raman parameters from our initial fit of the AC data alone, and thus we suspect that in cases where relaxation data only curve without displaying a clear power-law temperature dependence that the Raman parameters are perhaps less reliable.] To avoid over-parameterisation when modelling the Direct and QTM processes (see below), we fix *U*_eff_, *τ*_0_, *C* and *n* from here onwards. Hence, fitting the experimental DC relaxation data for **1** and **1a** simultaneously (*H*_ext_ ≥ 5 kOe, *T* ≤ 5 K) with Eq. (1) (*U*_eff_, *τ*_0_, *C*, and *n* fixed from above and *τ*^−1^_QTM_ = 0 for these DC data) gives *D* = 7(3) × 10^−13^ s^−1^ Oe^−*m*^ K^−*l*^, *m* = 2.20(5) and *l* = 1.23(5) (Figs. 4b, c). The fitted parameter values for the Direct process are in reasonable agreement with those suggested by Abragam and Bleaney^17^ who approximated *m* = 4 and *l* = 1, and gave an order-of-magnitude guess for *D* ~ (*gμ*_B_)^4^ s^−1^ Oe^−4^ K^−1^; the latter gives ca. 7.6 × 10^−13^ s^−1^ Oe^−4^ K^−1^ for *g* = 20 (appropriate when the field is along the quantisation axis of the *m*_*J*_ = ± 15/2 ground Kramers doublet). The deviation from *τ*^−1^ ∝ *H*^4^*T* could be because the approximation of $\left| {E_{ + 15/2} - E_{ - 15/2}} \right| \ll k_{\mathrm B}T$ (ref.^34^) is not satisfied for **1** when the field is along the quantisation axis in this field and temperature regime (*H*_ext_ ≥ 5 kOe, *T* ≤ 5 K), or because of the presence of hyperfine interactions which can give rise to *τ*^−1^ ∝ *H*^2^*T* behaviour^20^.

### The field and temperature dependence of QTM

**Fig. 5:**
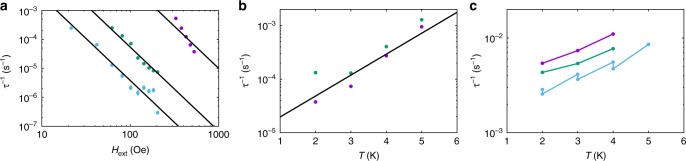
Low temperature and low field magnetic relaxation. **a** Magnetic relaxation rate measured by DC magnetometry (note the log-log scale) for **1** (purple circles), **1a** (green circles) and **1b** (light blue circles) at 2 K; black lines are best fits to $\tau _{\mathrm{QTM}}^{ - 1} = {{AH}}^{ - i}$ with *i* = 2.9(2), *A*_**1**_ = 5(1) × 10^3^, *A*_**1a**_ = 34(3) and *A*_**1b**_ = 2.4(4) s^−1^ Oe^*i*^. **b** Magnetic relaxation rate measured by DC magnetometry (note the log-lin scale) for **1** at 526(1) (purple circles) and for **1a** at 79(1) (green circles); black line is a best fit to $\tau _{\mathrm{QTM}}^{ - 1} = {{A{\mathrm e}}}^{jT}$ with *j* = 0.9(2) K^−1^ and *A* = 8(5) × 10^−6^ s^−1^. **c** Magnetic relaxation rate measured by DC magnetometry (note the log-lin scale) for **1** at 86(1) (purple circles), 126(1) (green circles) and 176(10) Oe (light blue circles); solid lines are a guide for the eye, there are two data points at 2, 3, and 4 K for the 176(10) Oe data, giving the appearance of a zig-zag line. Error bars are mostly within the data points

Focussing now on the low field, low temperature data (*H*_ext_ ≤ 1 kOe, *T* ≤ 8 K), we observe that the data in the regions 300 Oe ≤ *H*_ext_ ≤ 600 Oe for **1**, 70 Oe ≤ *H*_ext_ ≤ 200 Oe for **1a** and 20 Oe ≤ *H*_ext_ ≤ 200 Oe for **1b** have power-law dependencies on magnetic field with similar slopes (at *T* = 2 K, Figs. 3b and 5a), and that the isotherms are not superimposed in these field ranges (Fig. 3a; Supplementary Figs. 10–12); the former feature is ascribed to suppression of QTM and is commonly modelled with Eq. (2) (refs.^20,35,36^.), while the latter feature directly indicates a temperature dependence that is at odds with the usual description of QTM as a temperature-independent process. While Eq. (2) does not explicitly include temperature, the original form of this expression^37,38^ can be written as Eq. (3), and contains a term *η*^−1^ representing the characteristic phonon collision rate of the lattice; a term that must have some dependence on temperature. In Eq. (3), *ω* is a perturbation that allows QTM by mixing the two components of the ground doublet, related to the tunnelling gap *ħω*.

2 $$\tau _{\mathrm{QTM}}^{ - 1} = \frac{{Q_1}}{{1 + Q_2H^2}}$$

3 $$\tau _{\mathrm{QTM}}^{ - 1} = \frac{{\left( {2\omega } \right)^2\eta }}{{1 + \left( {\eta g_{\mathrm{eff}}\mu _{\mathrm B}\hbar ^{ - 1}H} \right)^2}}$$

However, in this strongly field-dependent regime we observe a power-law dependence on field that is not *τ*^−1^ ∝ *H*^*−*2^*T* as suggested by Eq. (3) (Fig. 5a). To model our data we must vary the field exponent whilst maintaining a dimensionally-consistent expression (i.e., *τ*^−1^_QTM_ must be a rate in units of s^−1^, even when *H* = 0), and thus the exponents in the numerator must also be allowed to vary; hence we define $\tau _{\mathrm{QTM}}^{ - 1} = \frac{{\left( {2\omega } \right)^i\eta ^{i - 1}}}{{1 + \left( {\eta g_{\mathrm{eff}}\mu _{\mathrm B}\hbar ^{ - 1}H} \right)^i}}$, which in this field-activated regime can be approximated as $\tau _{\mathrm{QTM}}^{ - 1} \approx \left( {\frac{{2\hbar \omega }}{{g_{\mathrm{eff}}\mu _{\mathrm B}}}} \right)^i\eta ^{ - 1}H^{ - i}$. Fitting the field dependence of the *T* = 2 K relaxation rates (the other relaxation mechanisms are insignificant in this field and temperature regime) for **1**, **1a**, and **1b** with $\tau _{\mathrm{QTM}}^{ - 1} = AH^{ - i}$ (with independent $A = \left( {\frac{{2\hbar \omega }}{{g_{\mathrm{eff}}\mu _{\mathrm B}}}} \right)^i\eta ^{ - 1}$ coefficients for each sample), we find that *i* ~ 3 (Fig. 5a). While obtaining a direct measurement of the temperature dependence of the phonon collision rate for **1** is unfeasible, approximate values have been obtained for silicon (Supplementary Fig. 13)^39–41^; these data show that *η*^−1^ has an exponential temperature dependence, $\eta ^{ - 1} = B{\mathrm e}^{jT}$, with *B* ~ 5 × 10^7^ s^−1^ and *j* ~ 0.01 K^−1^. This value of *j* would lead to QTM with a near-temperature-independent region, as observed in our AC data for **1** (Fig. 2b); therefore, we fix *j* = 0.01 K^−1^ based on the silicon data as an order-of-magnitude estimate. In the middle of the strongly field-dependent regimes for **1** and **1a** (at *H*_ext_ = 526(1) and 79(1) Oe, respectively, and *T* ≤ 5 K where QTM dominates so that $\tau ^{ - 1} \approx \left( {\frac{{2\hbar \omega }}{{g_{\mathrm{eff}}\mu _{\mathrm B}}}} \right)^i\eta ^{ - 1}H^{ - i}$), the relaxation rates show an exponential temperature dependence as expected (Fig. 5b), but in this regime we find a value of *j* that is almost two orders of magnitude larger than that for silicon (Fig. 5b). We do not currently understand why such strong temperature dependence manifests in the presence of strong field dependence; further studies of **1** and other large-*U*_eff_ SMMs will be required to understand these observations.

With an expression proposed for QTM relaxation, can this model explain the near field-independence of the relaxation rate at the lowest fields? Such behaviour could have two origins: (i) small external fields are not strong enough to supress relaxation by QTM (i.e., $\left( {\eta g_{\mathrm{eff}}\mu _{\mathrm B}\hbar ^{ - 1}H} \right)^i \ll 1$), or (ii) the internal dipolar field *H*_int_ acts to mitigate QTM in concert with *H*_ext_. Luckily, these two situations are easily distinguishable: for (i) we would have $\tau _{\mathrm{QTM}}^{ - 1} \approx \left( {2\omega } \right)^i\left( {B^{ - 1}{\mathrm e}^{ - jT}} \right)^{i - 1}$ which has a negative exponential dependence on temperature (as *i* > 1 and *j* > 0), and for (ii) we would have $\tau _{\mathrm{QTM}}^{ - 1} \approx \left( {\frac{{2\hbar \omega }}{{g_{\mathrm{eff}}\mu _{\mathrm B}}}} \right)^iB{\mathrm e}^{jT}H^{ - i}$ which has a positive exponential dependence on temperature. Examination of the temperature dependence of the relaxation rate for **1** at *T* ≤ 5 K and *H*_ext_ = 86(1), 126(1), and 176(10) (i.e., in the near field-independent region and at temperatures where other relaxation mechanisms are negligible) shows a positive exponential form (Fig. 5c), demonstrating that the lack of strong field dependence here is due to mitigation of QTM by internal dipolar fields. This conclusion is also supported by the movement of the low-field plateau to a smaller field at ~12% dilution in **1a** and loss of the plateau entirely at ~3% dilution in **1b** (Fig. 3b). Therefore, we propose to model the overall field-dependent and temperature-dependent relaxation with Eq. (4), where the QTM term is augmented to reflect the internal dipolar field. We consider the internal field *H*_int_ to have a random orientation and thus, coupled with the random powder distribution of our sample, we define *H*_tot_ = max(*H*_ext_, *H*_int_). Furthermore, we assume *H*_int_ has a Gaussian distribution around its average value *μ* with standard deviation *σ*. By examining the field dependence of the 2 K data (Supplementary Fig. 15), we can approximate that *μ*_**1**_ = 200 and *σ*_**1**_ = 50 Oe for **1**, *μ*_**1a**_ = 50 and *σ*_**1a**_ = 10 Oe for **1a**; as we do not observe a low-field plateau for **1b**, *H*_int,**1b**_ < 10 Oe.

4 $$\tau ^{ - 1} = \tau _0^{ - 1}{\mathrm e}^{ - U_{\mathrm{eff}}/T} + CT^n + DH_{\mathrm{ext}}^mT^l + \frac{{\left( {2\omega } \right)^i\eta ^{i - 1}}}{{1 + \left( {\eta g_{\mathrm{eff}}\mu _{\mathrm B}\hbar ^{ - 1}H_{\mathrm{tot}}} \right)^i}}$$

**Fig. 6:**
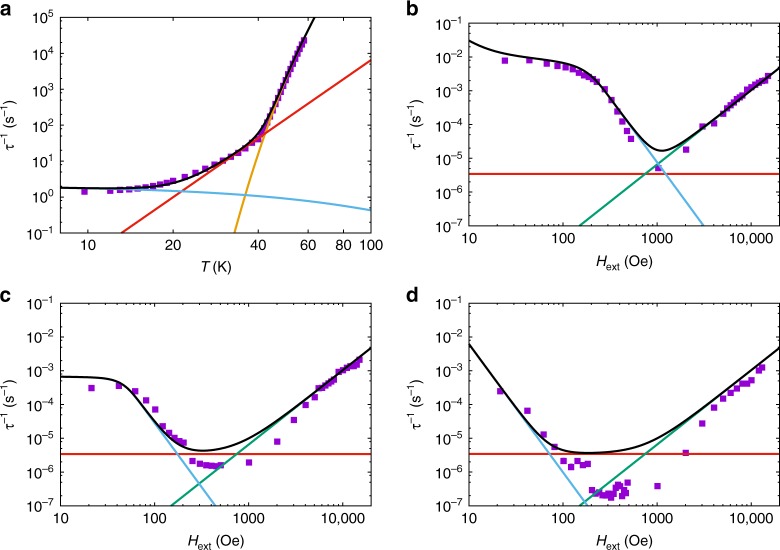
Modelling of magnetic relaxation. **a** Magnetic relaxation rate measured by AC magnetometry for **1** at *H*_ext_ = 0 Oe (note the log–log scale). **b** Magnetic relaxation rate measured by DC magnetometry for **1** at 2 K (note the log–log scale). **c** Magnetic relaxation rate measured by DC magnetometry for **1a** (note the log–log scale). **d** Magnetic relaxation rate measured by DC magnetometry for **1b** (note the log–log scale). For all plots: black line is a fit with Eq. (4) using the parameters in Table 1; orange line is the Orbach component alone, red line is the Raman component alone, green line is the Direct component alone, blue line is the QTM component alone. Error bars are within the data points

This QTM model has far too many parameters to find a fully-optimised parameterisation for our dataset. Therefore, based on the well-defined shapes of the 2 K data, we fix the approximate values for *H*_int_ determined above (Supplementary Fig. 15, with *H*_int,**1b**_ = 0). Furthermore, although the *m*_*J*_ = ± 15/2 ground Kramers doublet has 0 ≤ *g*_eff_ ≤ 20, molecules with *H*_ext_ perpendicular to their anisotropy axis will not be magnetised and therefore do not contribute to the measured relaxation dynamics; thus we assume *g*_eff_ ~ 20. Finally, as mentioned above, we fix the value of *j* to that for silicon (*j* = 0.01 K^−1^) as an order-of-magnitude estimate, but we allow the absolute magnitude of the phonon collision rate to vary via the pre-factor *B*. Hence, we fit all of our AC and DC data simultaneously by varying *ω*, *ω*_**1a**_, *ω*_**1b**_, *B* and *i* (with parameters for the Orbach, Raman and Direct processes fixed) to obtain excellent fits of the relaxation dynamics over 11 orders of magnitude (Fig. 6; Supplementary Figs. 16–23; Table 1). [Note: our fits deviate from the experimental data at intermediate fields for **1a** and **1b** (e.g., in Fig. 6b–d); this is due to an overestimation of the Raman relaxation rate (clearly seen by the red line having too large a value), which we have not re-refined after consideration of the Direct and QTM processes.] We find *B* = 2.6(7) × 10^8^ s^−1^ which is approximately five times larger than that for silicon; at 50 K, where relaxation *via* the Orbach mechanism dominates, the phonon collision time scale is $\eta = B^{ - 1}{\mathrm e}^{ - jT}$ = 2.3(7) × 10^−9^ s, which is on a similar order of magnitude as *τ*_0_ (3(1) × 10^−12^ s) as suggested by Fort et al.^34^ Of most interest, however, are the QTM perturbations: fitting our experimental data with Eq. (4), we find *ω*_**1**_ = 2.4(5) × 10^7^, *ω*_**1a**_ = 0.34(8) × 10^7^ and *ω*_**1b**_ = 0.14(4) × 10^7^ rad s^−1^ which correspond to tunnelling gaps of *ħω*_**1**_ = 1.3(3) × 10^−4^, *ħω*_**1a**_ = 0.18(4) × 10^−4^ and *ħω*_**1b**_ = 0.07(2) × 10^−4^ cm^−1^. These effective QTM tunnelling gaps are purely empirical and in principle account for all experimental contributions to QTM. However, their small magnitude indicates that only minimal mixing between the two ground state wavefunctions is required to facilitate efficient QTM in this dysprosium(III) SMM.

**Table 1:** Model magnetic relaxation parameters

Mechanism	Parameter	1	1a	1b
Orbach	*τ*_0_ (10^−12^ s)	3 (1)		
	*U*_eff_ (K)	950 (20)		
Raman	*C* (10^−8^ s^−1^ K^−*n*^)	7.8 (8)		
	*n*	5.46 (4)		
Direct	*D* (10^−13^ Oe^−*m*^ K^−*l*^)	7 (3)		
	*m*	2.20 (5)		
	*l*	1.23 (5)		
QTM	*ω* (10^7^ rad s^−1^)	2.4 (5)	0.34 (8)	0.14 (4)
	*i*	3.8 (2)		
	*B* (10^8^ s^−1^)	2.6 (7)		
	*j* (K^−1^)	0.01 (fixed)		
	*g*_eff_	20 (fixed)		
	*μ* (Oe)	200 (fixed)	50 (fixed)	0 (fixed)
	*σ* (Oe)	50 (fixed)	10 (fixed)	–

### Origins of QTM

We have found that the magnetic relaxation rate for **1** at *T* ≤ 16 K and *H*_ext_ = 0 approaches a temperature-independent regime (Fig. 2b); this is not compatible with a two-phonon Raman process. We have found that for *T* ≤ 5 K and *H*_ext_ ≥ 1 kOe the relaxation rate behaves according to a single-phonon Direct process; such a process must vanish for a Kramers ion in zero field. Taking these two observations together, the enhanced relaxation for *T* ≤ 16 K and *H*_ext_ < 1 kOe must be a QTM process. We have been able to model this QTM process using a field- and temperature-dependent expression, and used this model to show that the low-field plateau is due to the effect of an internal dipolar field. From fits using this model, we have determined the QTM tunnelling gaps to be 1.3(3) × 10^−4^, 0.18(4) × 10^−4^ and 0.07(2) × 10^−4^ cm^−1^ for **1**, **1a**, and **1b**, respectively. As *ω* changes as a function of dilution, QTM must owe, at least in part, to the effect of internal dipolar fields; this can be directly observed by the reduction of the zero-field step in the 2 K hysteresis upon dilution (Fig. 2a) and that a transition to a near-temperature-independent regime is not observed in our AC experiments for **1a** in contrast to **1**. Therefore, we have a rather strange situation where the internal dipolar field is both partially responsible for the QTM tunnelling gap, as well as mitigating QTM in the near-zero-field regime. The simultaneity of these two functions is clearly shown by our 2 K DC relaxation data (Fig. 3b): if there was no change in the QTM tunnelling gap upon dilution, and only the internal field was altered, then the 2 K relaxation dynamics for **1a** would be coincident with those for **1** excepting at low fields where the rate for **1a** would continue to rise with reducing *H*_ext_ until it plateaued at a smaller field corresponding to the reduced value of *H*_int_. However, we observe a clear reduction of the relaxation rate in the strongly-field activated region upon dilution, which directly indicates a reduction of *ω*. These observations cannot be explained by the effect of hyperfine coupling: averaging over all Dy-containing molecules in **1**, **1a**, and **1b**, the statistical distribution of spin-active nuclei will be the same, and thus the effects of hyperfine coupling should not change upon dilution. Therefore, we suggest that the internal dipolar field is responsible for these observations. We note, however, that an internal dipolar field is not the only way to mitigate QTM: insulation of an SMM from its environment to reduce the phonon density of states^24^ and to reduce collisions with conduction electrons^23^ are especially important when SMMs are adsorbed to surfaces.

However, given the large difference in zero-field relaxation for the dysprosocenium SMM^2^ compared to other recent large-barrier dysprosium(III) SMMs^6–13^, including **1**, internal dipolar fields cannot be the sole cause of QTM. A common suggestion is that QTM for dysprosium(III) SMMs could also be due to hyperfine coupling with spin active ^163^Dy and ^161^Dy nuclei having *I* = 5/2 (see refs.^42–45^); we also do not believe this to be the sole cause of QTM in monometallic dysprosium(III) SMMs for two reasons: (i) the dysprosocenium SMM displays a much-reduced zero-field relaxation step at 2 K compared to other recent large-barrier dysprosium(III) SMMs^6–13^, all containing naturally abundant Dy, and (ii) enrichment with nuclear-spin-free ^164^Dy does not remove the zero-field step in the magnetic hysteresis of SMMs compared to the nuclear-spin-active ^163^Dy isotope^42–45^.

**Fig. 7:**
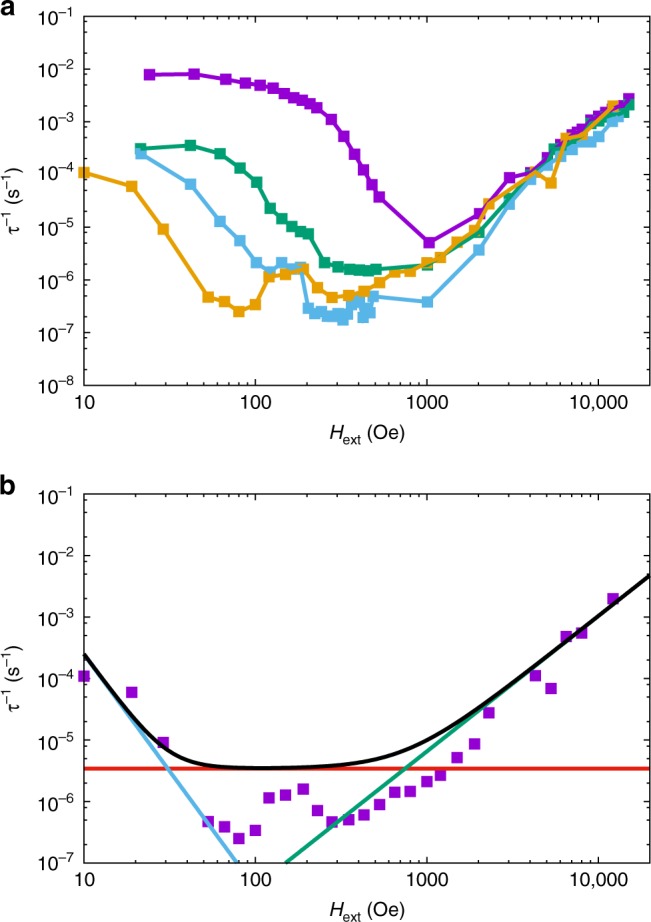
Field dependence of magnetic relaxation rate with ^164^Dy enrichment. **a** Magnetic relaxation rate for **1** (purple), **1a** (green), **1b** (light blue) and ~4% ^164^Dy@**2** (yellow; note log-log scale) at 2 K. Solid lines are a guide for the eye. **b** Magnetic relaxation rate for ~4% ^164^Dy@**2** (note the log-log scale). Black line is a fit with Eq. (4) using the parameters in Table 1, however with *ω*_**164**_ = 0.06(2) × 10^7^ rad s^−1^; red line is the Raman component alone, green line is the Direct component alone, blue line is the QTM component alone. Error bars are within the data points

These isotopic experiments show that the relaxation rate at low temperatures and low fields is slowed upon enrichment with ^164^Dy, however the precipitous zero-field drop is not removed. In order to probe directly the contribution of hyperfine coupling to the QTM of **1**, we have prepared the ^164^Dy isotopomer (96.80% ^164^Dy) diluted into the diamagnetic yttrium(III) analogue, ~4% ^164^Dy@**2** (see Methods), and performed DC decay experiments at 2 K (Supplementary Table 23). We observe that the relaxation rates are similar to those obtained for the similarly dilute naturally abundant Dy sample **1b** at high fields (*H*_ext_ > 100 Oe, Fig. 7a), indicating that the Direct and Raman relaxation processes are not appreciably influenced by hyperfine coupling with ^161/163^Dy nuclear spins. Furthermore, we still observe an increase in relaxation rate with decreasing field for *H*_ext_ < 100 Oe with a similar slope to **1b**, and that a field-independent regime is not reached down to at least 10 Oe. These two features indicate respectively that: (i) QTM is still active at low temperatures and fields despite ~4% magnetic dilution and no Dy-based nuclear spin (this is also exemplified by the presence of a zero-field step in the 2 K magnetic hysteresis trace of ~4% ^164^Dy@**2**, Supplementary Fig. 13), and (ii) the dipolar field is still *H*_int_ < 10 Oe, which is consistent with the natural abundance Dy analogue with a similar dilution, **1b** (ca. 3 vs. 4%). However, there is a marked decrease in the absolute value of the relaxation rates in the strongly field active regime at low fields compared to **1b**: this must be due to the effect of removing Dy nuclear spins. By fixing all parameters determined above for **1**, **1a**, and **1b** (Table 1), we can fit the relaxation data for ~4% ^164^Dy@**2** with Eq. (4) by varying only the QTM term *ω*. We obtain an excellent fit of the low-field data for *ω*_**164**_ = 0.06(2) × 10^7^ rad s^−1^ (Fig. 7b, corresponding to an effective tunnelling gap of *ħω*_**164**_ = 0.03(1) × 10^−4^ cm^−1^); clearly this parameter is less than half that for **1b** (*ω*_**1b**_ = 0.14(4) × 10^7^ rad s^−1^), even though both samples have a similar magnetic dilution. This directly indicates that Dy nuclear spins must have some influence on the effective QTM rate, as found previously^42–45^, however that QTM is still clearly present despite removal of Dy nuclear spins and at high dilutions.

Therefore, we contend that internal dipolar fields and hyperfine coupling (including other nuclei beyond ^161/163^Dy, such as ^1^H) play supporting roles in QTM by removing the Kramers degeneracy, such that a more efficient coupling mechanism can have an effect. Far from providing a definitive theorem on the origin of the QTM here, we note that the non-axial CF operators can mix the |±15/2> states via excited states in low orders of perturbation theory (as these operators mix states with Δ*m*_*J*_ = ±1, ±2, ±3, ±4, ±5, and ±6), compared to both hyperfine coupling and dipolar fields that can only mix the |±15/2> states in high orders of perturbation theory (as these operators only mix states with Δ*m*_*J*_ = ±1). Therefore, the non-axial CF is much more likely to mix the ground state wavefunctions, and we propose that the dynamic CF, as induced by molecular vibrations, has a crucial role in the efficient QTM mechanism for dysprosium(III) SMMs. Under the paradigm that internal dipolar fields and hyperfine coupling play only supporting roles of in QTM, it becomes clear why the zero-field step in magnetic hysteresis loops for dysprosium(III) SMMs can be modified but not completely removed by dilution or isotopic enrichment: the degree to which the Kramers degeneracy is removed is important, but it is practically impossible to ensure the absence of any stray magnetic field (including dipolar and hyperfine fields), and thus the intrinsic QTM driven by the dynamic CF remains prevalent. Thus, we propose that engineering the molecular structure to reduce the facility of vibrational modes that generate non-axial perturbations in the dynamic CF holds the key to achieving molecular magnetic hysteresis above the temperature of liquid nitrogen; this is the chemical equivalent of reducing the low-energy phonon density of states that couple to the magnetic centre, which has been shown to completely remove the QTM in low-dimensional molecular or atomic magnets^24,46^.

Through a field- and temperature-dependent study of the magnetic relaxation rate for the large *U*_eff_ SMM [Dy(^t^BuO)Cl(THF)_5_][BPh_4_]·2THF, we have found four distinct relaxation processes that each dominate in different regimes. Above 16 K, field-independent Raman and Orbach relaxation mechanisms dominate, while below 6 K we find a strong dependence of the relaxation rate on the applied magnetic field, providing clear evidence of Direct and QTM relaxation processes. By explicitly including the temperature dependence of the phonon collision rate, we have modelled the QTM process and therefore the relaxation dynamics over 11 orders of magnitude (Fig. 6; Supplementary Figs. 16–23). We find that the perturbation allowing QTM within the |±15/2 > ground doublet is related to the magnitude of the internal dipolar field and to the presence of *I* = 5/2 nuclear spins from ^161/163^Dy nuclei, but reason that neither dipolar fields nor hyperfine coupling can be the sole cause of this effect for dysprosium(III) SMMs. We suggest, rather, that efficient zero-field QTM is related to the dynamic non-axial CF. This suggests that QTM processes in dysprosium(III) SMMs may be modulated by molecular design, rather than by dilution or enrichment, thus returning the future prospects for technologically useful SMMs back into the hands of imaginative synthetic chemists.

## Methods

### Synthesis and structure

All reactions were carried out under a dry and oxygen-free argon atmosphere by using Schlenk techniques or in a glovebox. Toluene, Tetrahydrofuran and hexane were dried and degassed by standard techniques. Anhydrous DyCl_3_ and YCl_3_ were prepared according to the literature procedure^47^. ^164^Dy_2_O_3_ (^164^Dy isotopic content 96.80%) was purchased from Euriso-top and used as received to prepare ^164^DyCl_3_ following literature procedures^48^. The ^164^Dy-enriched sample was digested with HNO_3_ and analysed by ICP-MS with an Agilent 7500cx by Mr Paul Lygoth at The University of Manchester: isotopic fingerprint for the ^164^Dy sample was: 96.62% ^164^Dy, 2.27% ^163^Dy, 0.64% ^162^Dy, 0.37% ^161^Dy, 0.05% ^160^Dy, 0.02% ^158^Dy, 0.03% ^156^Dy. NaO^t^Bu, and NaBPh_4_ were commercial available and used without further treatment. Infrared spectra were collected on a Thermo Fisher Nicolet 6700 FT-IR spectrometer using ATR (Attenuated Total Reflectance) method. Absorption maxima (*ν*_max_) are reported in wavenumbers (cm^−1^).

[Dy(^t^BuO)Cl(THF)_5_][BPh_4_]·2THF **1** was prepared by small modification of the synthesis of [Y(^t^BuO)Cl(THF)_5_][BPh_4_]·2THF **2** (see ref.^28^). Reaction of DyCl_3_ (0.5 mmol, 134 mg), NaO^t^Bu (0.5 mmol, 48 mg) and NaBPh_4_ (0.5 mmol, 171 mg) in THF gives a cloudy solution, which was filtrate and the solvent was removed by vacuum to give a white powder of the product (480 mg, 87.7% yield). Crystals suitable for X-ray diffraction were grown by layering saturated THF solution of **1** with hexane at −35 °C. IR (KBr, cm^−1^): 3056 (m), 2982 (s), 2883 (w), 1943 (w) 1877(w), 1818 (w), 1752 (w), 1651 (w), 1591 (m), 1580 (m), 1480 (m), 1457 (m), 1428 (w), 1400 (w), 1353 (w), 1240 (m), 1185 (w), 1147 (w), 1068 (m), 1031 (w), 997 (w),870 (m, br), 773 (w), 743 (s), 704 (s), 643 (m), 638 (w).

The synthesis of [Y(^t^BuO)Cl(THF)_5_][BPh_4_]·2THF **2** was the same as **1** with anhydrous DyCl_3_ replaced by 0.5 mmol of anhydrous YCl_3_. Yield 390 mg, 76.4%. IR (KBr, cm^−1^): 3055 (m), 2982 (s), 2896 (M), 1947 (w) 1879(w), 1819 (w), 1769 (w), 1645 (w), 1604 (m), 1591 (s), 1562 (m), 1495 (m), 1479 (s), 1457 (m), 1427 (m), 1377 (w), 1354 (m), 1266 (w), 1240 (m), 1198 (m, br), 1146 (w), 1066 (w), 1030 (s), 997 (w), 918 (w), 865 (s), 776 (m), 743 (s), 705 (s), 674 (w), 625 (w).

The synthesis of Dy@[Y(^t^BuO)Cl(THF)_5_][BPh_4_]·2THF was the same as **1** with anhydrous DyCl_3_ replaced by 0.475 mmol of anhydrous YCl_3_ and 0.025 mmol of anhydrous DyCl_3_. IR (KBr, cm^−1^): 3057 (m), 2981 (s), 2897 (w), 1959 (w) 1887(w), 1819 (w), 1770 (w), 1646 (w), 1579 (s), 1561 (w), 1480 (s), 1458 (m), 1427 (s), 1398 (m), 1354 (w), 1225 (w), 1185 (m, br), 1152 (w), 1067 (m), 1029 (m), 919 (m),868 (s), 776 (w), 743 (s), 715 (s), 673 (w), 625 (m).

Cation: all the complexes lose co-crystalized and coordinated THF molecule after a few months even stored in a dry glovebox^28^. All measurements were carried out with freshly prepared crystalline samples.

### X-ray crystallography

All data were recorded on a Bruker SMART CCD diffractometer with MoK_α_ radiation (*λ* = 0.71073 Å). The structures were solved by direct methods and refined on *F*^2^ using SHELXTL^49^.

### CASSCF-SO electronic structure

MOLCAS 8.0^29^ was employed to perform CASSCF-SO calculations on the cation in **1** based on the X-ray structure with no optimisation. Basis sets from the ANO-RCC library were employed^50,51^, with VTZP quality for the Dy atom, VDZP quality for the Cl and O atoms, and VDZ quality for all C and H atoms. The two electron integrals were Cholesky decomposed with a threshold of 1 × 10^−8^. The state-averaged CASSCF orbitals of the sextets, quartets and doublets were optimised with 21, 224, and 490 states, respectively, with the RASSCF module. The spin-orbit coupling Hamiltonian was then constructed and diagonalised in the basis of 21, 128, and 130 sextets, quartets and doublets, respectively, with the RASSI module. The crystal field decomposition of the ground *J* = 15/2 multiplet was performed with the SINGLE_ANISO module^52,53^.

### Magnetometry

Magnetisation measurements were carried out with a Quantum Design MPMS-XL7 SQUID magnetometer, while hysteresis measurements were carried out on a Quantum Design MPMS3-VSM SQUID (excepting that for ~4% ^164^Dy@**2**, which was measured on a MPMS-XL7 SQUID). Freshly prepared polycrystalline samples were embedded in eicosane to avoid any field induced crystal reorientation, and contained in a flame-sealed quartz NMR tube. Diamagnetic corrections were applied for the eicosane and for the molecule, the latter being calculated from the Pascal constants.

We extract the magnetic relaxation times from AC susceptibility using the modified Debye model $\chi \left( \omega \right) = \chi _S + \frac{{\chi _T - \chi _S}}{{1 + \left( {i\omega \tau } \right)^{1 - \beta }}}$ (Supplementary Table 4)^18^. The *β* parameters range from ca. 0.03 to 0.3, being at the larger end of the range at the lowest temperatures; this indicates a broadening distribution of relaxation times, likely owing to the inhomogeneity of the QTM process.

We extract the magnetic relaxation times from DC magnetisation decay using a stretched exponential $M\left( t \right) = M_{\rm eq} + \left( {M_0 - M_{\rm eq}} \right){\mathrm e}^{ - \left( {t/\tau } \right)^\alpha }$ (Supplementary Tables 5–13^18^. We note that a single exponential is not adequate to model the data, and we have obtained stretch parameters similar to those reported previously^54,55^. At low and high fields at 2, 3 and 4 K, the extracted *M*_eq_ values compare extremely well to the theoretical values for a pure |±15/2> state (Supplementary Figure 7). For intermediate fields around 1 kOe, we find that the magnetic relaxation rate is so slow that equilibrium magnetisation could not be reached in an acceptable timeframe; in these cases we fix *M*_eq_ to the theoretical value scaled to the experimental *M*_sat_ value at *H*_*i*_ = 50 kOe.

### Data availability

CCDC 1450752 (**1**), and 1566471 (**2**) contain the supplementary crystallographic data for this paper. These data can be obtained free of charge via www.ccdc.cam.ac.uk/conts/retrieving.html (or from the Cambridge Crystallographic Data Centre, 12 Union Road, Cambridge CB2 1EZ, UK; fax: (+44)1223-336-033; or deposit@ccdc.cam.ac.uk). Magnetic data is available from N.F.C. on request.

## Electronic supplementary material

Supplementary Information

Peer Review File
