# Supplementary material for: Field- and temperature-dependent quantum tunnelling of the magnetisation in a large barrier single-molecule magnet
Source: Nat Commun. 2018 Aug 7;9:3134. doi: 10.1038/s41467-018-05587-6 (PMC6081483; doi:10.1038/s41467-018-05587-6)
Supplement: Supplementary file 1 — Supplementary Information [file 41467_2018_5587_MOESM1_ESM.pdf]

Supplementary Information for:

Field- and temperature-dependent quantum tunnelling of the magnetisation in a large barrier single-molecule magnet

Ding *et al.*

Contents

1. X-ray Crystallography	2
2. CASSCF-SO Electronic Structure	4
3. Magnetometry	5
4. Magnetic Modelling	115
5. Supplementary References	120

X-ray Crystallography

Supplementary Table 1. Crystallographic data for **1** and **2** at 100 K.

	**1**	**2**
**Empirical formula**	$C_{56}H_{85}BClDyO_8$	$C_{56}H_{85}BClYO_8$
**Formula weight (g mol^{-1})**	1094.99	1021.40
**Crystal system**	Monoclinic	Monoclinic
**Space group**	*P*2(1)/n	*P*2(1)/n
T **(K)**	100 (2) K	100 (2) K
a **(Å)**	13.5129(14)	13.6356(17)
b **(Å)**	12.4445(13)	12.4340(15)
c **(Å)**	32.491(3)	32.618(4)
α **(°)**	90	90
β **(°)**	90.243(2)	90.434(2)
γ **(°)**	90	90
V **(Å^3)**	5463.7(10)	5530.0(12)
Z	4	4
**ρ_{calcd} (g cm^{-3})**	1.331	1.227
**F(000)**	2292	2184
**Reflns collected**	50278	39183
**Unique reflns**	12784	9544
**R_{int}**	0.0209	0.0482
**Parameters / restraints**	90 / 607	128 / 607
**GOF**	1.160	1.049
**R_1 (all data)**	0.0629	0.0895
**R_1 (I>2sigma(I))**	0.0515	0.0585
**wR_2 (all data)**	0.1238	0.1578
**wR_2 (I>2sigma(I))**	0.1148	0.1423
**Largest diff. peak/hole (e Å^{-3})**	3.429/-2.866	1.075/-1.114

Supplementary Table 2: Selected bond lengths (Å) and angles (°) for **1** and **2**.

**1**		**2**	
Dy(1)-O(1)	2.043(4)	Y(1)-O(1)	2.042(3)
Dy(1)-O(2)	2.414(3)	Y(1)-O(2)	2.421(3)
Dy(1)-O(3)	2.412(3)	Y(1)-O(3)	2.414(3)
Dy(1)-O(4)	2.411(3)	Y(1)-O(4)	2.418(3)
Dy(1)-O(5)	2.426(3)	Y(1)-O(5)	2.412(3)
Dy(1)-O(6)	2.390(3)	Y(1)-O(6)	2.390(3)
Dy(1)-Cl(1)	2.6619(12)	Y(1)-Cl(1)	2.6608(11)
O(1)-Dy(1)-Cl(1)	178.26(9)	O(1)-Y(1)-Cl(1)	178.72(8)
O(5)-Dy(1)-Cl(1)	83.43(9)	O(4)-Y(1)-Cl(1)	83.91(7)
O(3)-Dy(1)-Cl(1)	85.49(10)	O(2)-Y(1)-Cl(1)	85.27(7)
O(4)-Dy(1)-Cl(1)	87.54(7)	O(5)-Y(1)-Cl(1)	87.55(7)
O(6)-Dy(1)-Cl(1)	88.05(9)	O(6)-Y(1)-Cl(1)	87.81(7)
O(2)-Dy(1)-Cl(1)	89.45(9)	O(3)-Y(1)-Cl(1)	89.16(7)
O(1)-Dy(1)-O(2)	89.53(13)	O(1)-Y(1)-O(3)	90.20(10)
O(1)-Dy(1)-O(4)	92.50(13)	O(1)-Y(1)-O(5)	92.33(11)
O(1)-Dy(1)-O(6)	93.61(12)	O(1)-Y(1)-O(6)	93.36(10)
O(1)-Dy(1)-O(5)	94.92(12)	O(1)-Y(1)-O(4)	94.84(10)
O(1)-Dy(1)-O(3)	95.52(14)	O(1)-Y(1)-O(2)	95.59(10)
O(6)-Dy(1)-O(3)	71.42(11)	O(6)-Y(1)-O(2)	71.43(10)
O(6)-Dy(1)-O(4)	71.53(10)	O(6)-Y(1)-O(5)	71.71(10)
O(2)-Dy(1)-O(5)	71.78(11)	O(3)-Y(1)-O(4)	71.75(10)
O(3)-Dy(1)-O(2)	71.94(11)	O(3)-Y(1)-O(2)	71.96(10)
O(4)-Dy(1)-O(5)	73.09(10)	O(5)-Y(1)-O(4)	72.82(10)

CASSCF-SO Electronic Structure

Supplementary Table 3. CASSCF-SO calculated electronic states for **1**. Dominant components of the wavefunctions in the ground $^6H_{15/2}$ basis (> 10%) rounded to the nearest 1%.

Energy (K)	g_x	g_y	g_z	Angle (°)	Wavefunction
0	0.00	0.00	19.88	--	$100\%\vert\pm 15/2\rangle$
571	0.02	0.02	16.97	0.6	$99\%\vert\pm 13/2\rangle$
808	1.79	4.35	15.29	89.1	$59\%\vert\pm 1/2\rangle + 22\%\vert\mp 1/2\rangle$
870	2.84	3.58	5.56	83.2	$56\%\vert\pm 3/2\rangle + 26\%\vert\mp 3/2\rangle$
895	0.06	1.88	12.10	3.4	$76\%\vert\pm 11/2\rangle$
940	0.70	2.61	7.29	19.8	$81\%\vert\pm 5/2\rangle$
1003	0.61	2.13	11.27	34.9	$65\%\vert\pm 7/2\rangle + 24\%\vert\pm 9/2\rangle$
1018	0.81	2.56	13.57	37.7	$69\%\vert\pm 9/2\rangle + 25\%\vert\pm 7/2\rangle$

Magnetometry

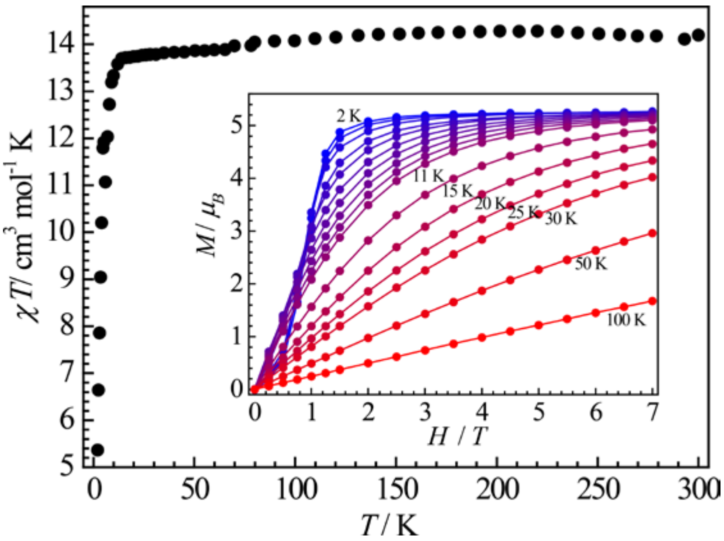

Supplementary Figure 1. Variable temperature magnetic susceptibility for **1** in a 1 kOe field. The high temperature limit for χT is 14.19 cm^3 mol^{-1} K at 300 K. Inset: Variable field magnetisation of **1**, at indicated temperatures from 2 (blue) to 100 (red) K. The saturation value at 2 K and 7 T is 5.26 μ_B. Note that the strange low field and low temperature profiles of the magnetisation data are due to slow magnetic relaxation.

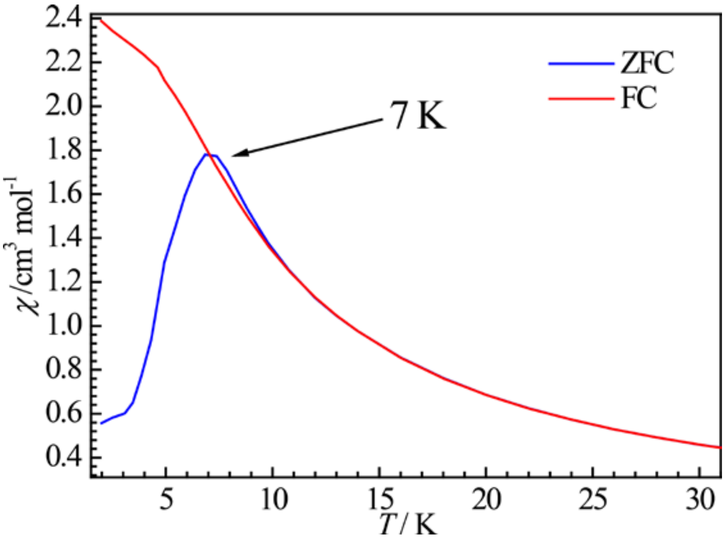

Supplementary Figure 2. Field cooled (FC) and zero FC (ZFC) magnetic susceptibility for **1** in a 2 kOe field.

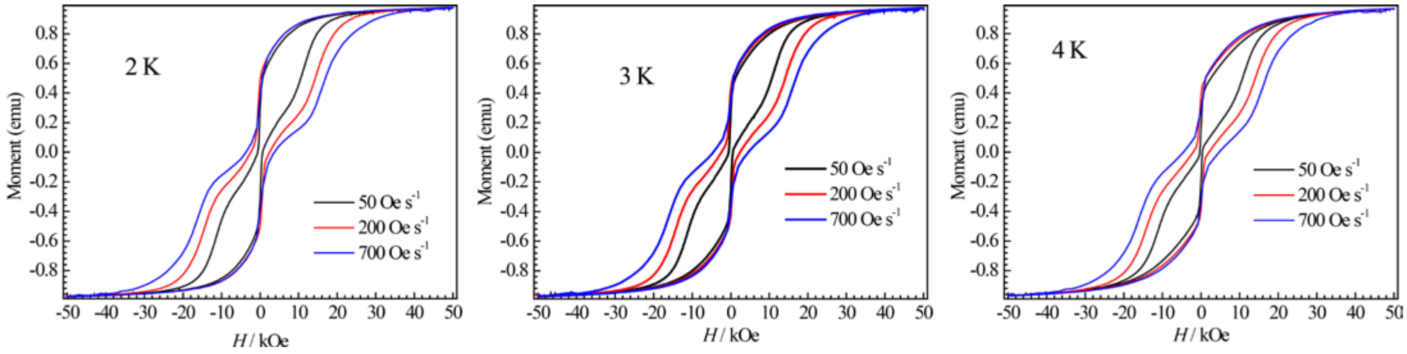

Supplementary Figure 3. Magnetic hysteresis for **1** from 2 K to 4 K at sweep rates of 50, 200 and 700 Oe s^{-1}.

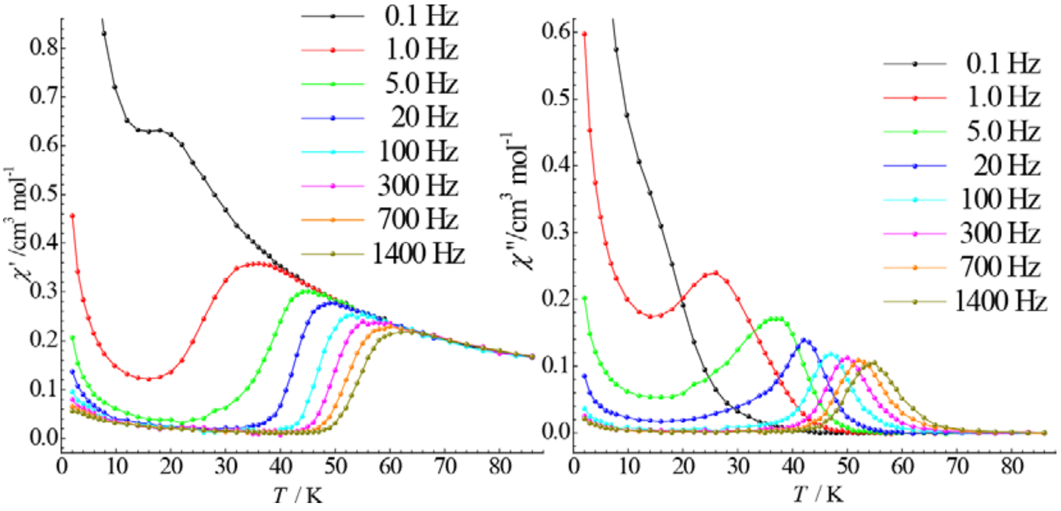

Supplementary Figure 4. In-phase (left) and out-of-phase (right) AC susceptibility for **1** in zero DC field, as a function of temperature.

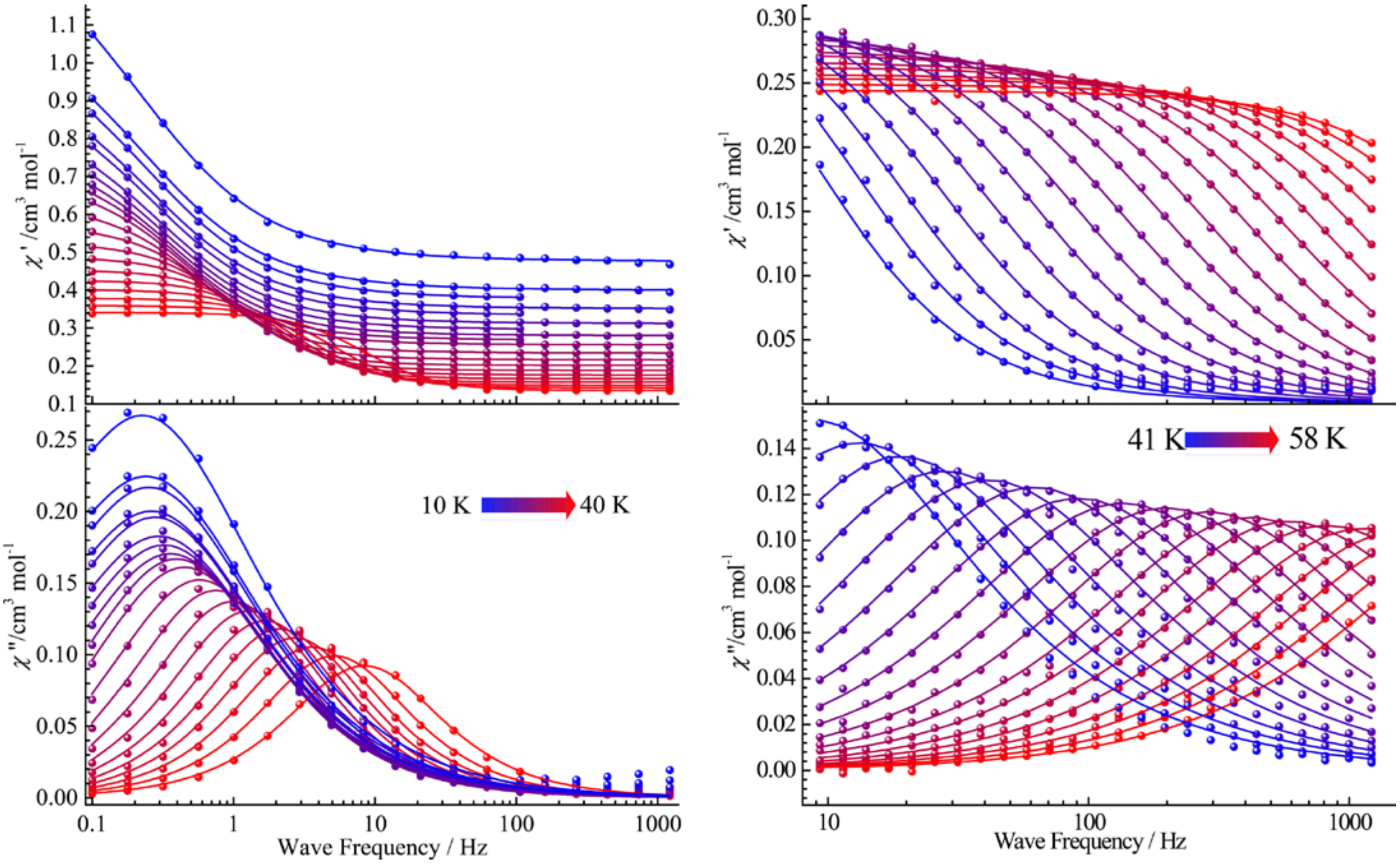

Supplementary Figure 5. In-phase (top) and out-of-phase (top) AC susceptibility for **1** in zero DC field, as a function of AC frequency.

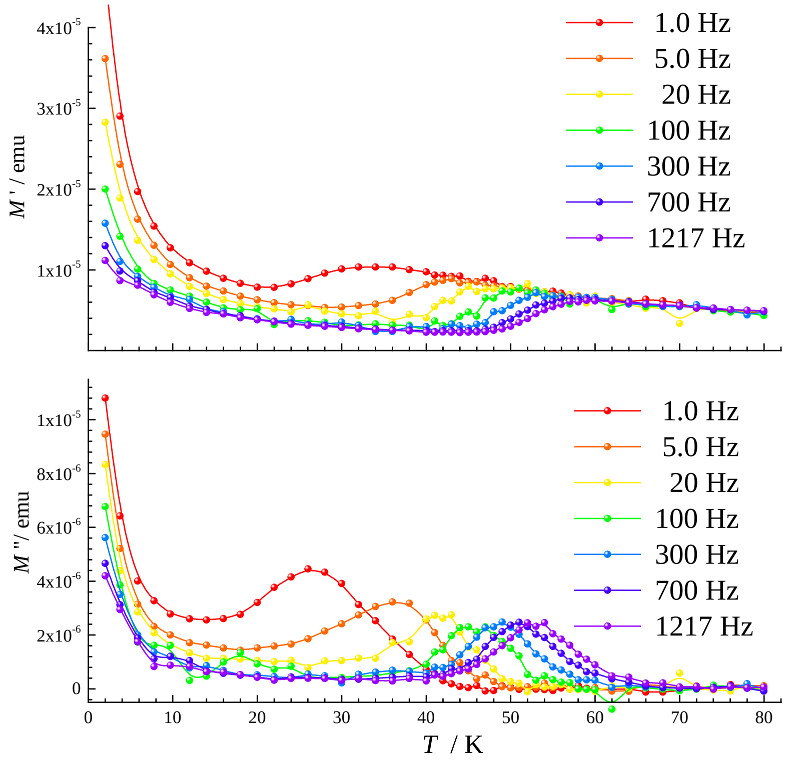

Supplementary Figure 6. In-phase (top) and out-of-phase (bottom) AC susceptibility for **1a** in zero DC field, as a function of temperature.

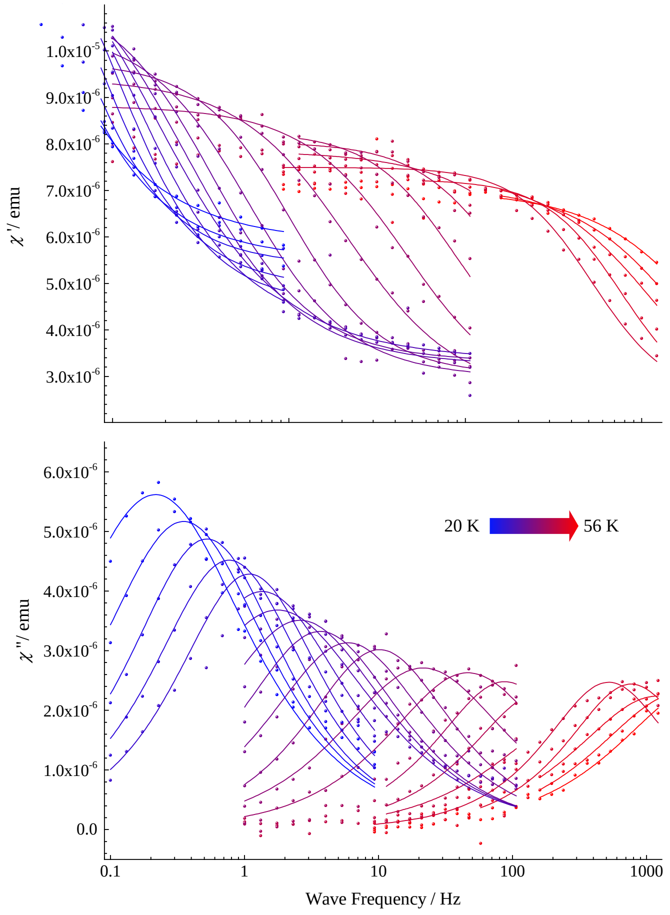

Supplementary Figure 7. In-phase (top) and out-of-phase (bottom) AC susceptibility for **1a** in zero DC field, as a function of AC frequency.

Supplementary Table 4. Debye parameters from AC susceptibility data for **1**.

***T* (K)**	**χ_T (cm^3 mol^{-1} K)**	**χ_S (cm^3 mol^{-1} K)**	***τ* (s)**	***β***
10	0.47737	1.34605	7.043E-01	0.28713
12	0.4006	1.11321	6.634E-01	0.27243
13	0.3781	1.05146	6.298E-01	0.25907
14	0.35168	0.96898	6.085E-01	0.2549
15	0.33437	0.92464	5.750E-01	0.2404
16	0.31253	0.85575	5.375E-01	0.23223
17	0.29653	0.80833	4.925E-01	0.21646
18	0.28058	0.76301	4.451E-01	0.20099
19	0.26904	0.72348	4.010E-01	0.07852
20	0.25607	0.68483	3.543E-01	0.16168
22	0.23479	0.62158	2.765E-01	0.13069
24	0.21689	0.56839	2.121E-01	0.09954
26	0.20163	0.52372	1.627E-01	0.07852
28	0.18843	0.48672	1.251E-01	0.06054

30	0.17666	0.45338	9.647E-02	0.04748
32	0.16681	0.42563	7.546E-02	0.03481
34	0.15747	0.40128	5.875E-02	0.03173
36	0.1493	0.37863	4.414E-02	0.02948
38	0.14217	0.35923	3.082E-02	0.03368
40	0.13885	0.34879	2.456E-02	0.07547
41	3.40573E-16	0.37459	1.779E-02	0.13039
42	4.84559E-16	0.35205	1.213E-02	0.13246
43	5.8617E-16	0.33778	8.400E-03	0.1333
44	6.63929E-16	0.32829	5.730E-03	0.14539
45	7.15702E-16	0.32018	3.870E-03	0.14899
46	8.46044E-16	0.3111	2.580E-03	0.14651
47	9.04354E-16	0.30279	1.750E-03	0.15442
48	9.49382E-16	0.29522	1.180E-03	0.15001
49	1.15109E-15	0.29006	8.070E-04	0.15025
50	1.361E-15	0.28336	5.560E-04	0.14471
51	1.39938E-15	0.27667	3.820E-04	0.14358
52	1.36785E-15	0.27301	2.730E-04	0.14512
53	1.57782E-15	0.26688	1.950E-04	0.14127
54	1.91798E-15	0.26256	1.410E-04	0.1389
55	2.55323E-15	0.25697	1.020E-04	0.13449
56	2.85547E-15	0.25371	7.620E-05	0.13472
57	3.75059E-15	0.2492	5.670E-05	0.13438
58	3.49597E-15	0.24434	4.390E-05	0.12269

Supplementary Table 5. Debye parameters from AC susceptibility data for **1a**.

T **(K)**	χ_T **(emu)**	χ_S **(emu)**	τ ***(s)***	β
55	6.2088E-7	7.03115E-6	5.63751E-5	0.17146
54	1.35311E-6	7.12672E-6	8.92685E-5	0.14712
53	1.75967E-6	7.36328E-6	1.32894E-4	0.14176
52	2.35414E-6	7.23303E-6	2.07567E-4	0
51	2.55509E-6	7.49591E-6	3.04864E-4	0
50	3.02465E-6	7.85989E-6	4.82138E-4	0.1524
48	3.03242E-6	8.0541E-6	9.69146E-4	0.05564
46	2.96985E-6	8.27072E-6	0.00183	0.04215
44	2.40261E-6	8.83764E-6	0.00343	0.12763
42	2.53693E-6	9.44422E-6	0.00743	0.15284
40	3.01109E-6	9.79118E-6	0.0157	0.07402
38	2.94509E-6	1.05206E-5	0.02694	0.12036
36	3.29675E-6	1.13277E-5	0.04329	0.11991
34	3.21284E-6	1.2354E-5	0.0612	0.16637
32	3.33522E-6	1.37413E-5	0.09049	0.21602
30	3.91586E-6	1.39434E-5	0.11763	0.14389
28	4.52843E-6	1.41496E-5	0.14627	0.07351
26	4.8698E-6	1.53572E-5	0.20772	0.09451
24	5.34896E-6	1.67904E-5	0.30425	0.10174
22	5.48487E-6	1.86412E-5	0.45276	0.15233
20	5.84036E-6	2.14013E-5	0.73519	0.20383

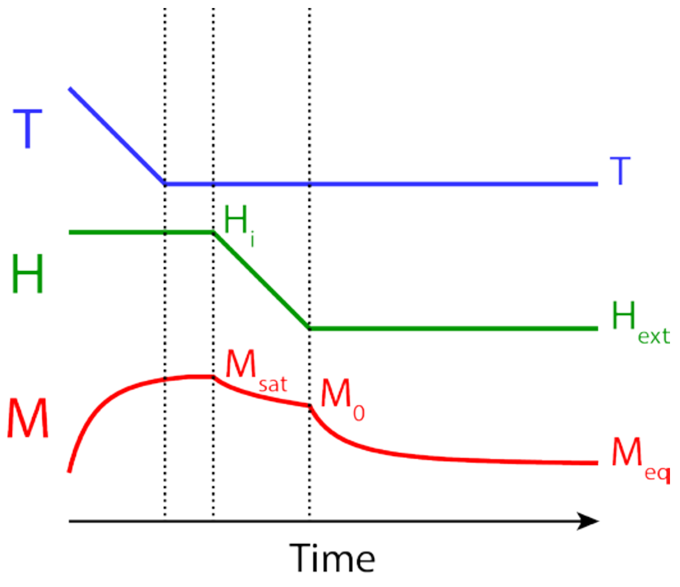

Supplementary Figure 8. Schematic of DC magnetisation decay experiments; samples were magnetised at low temperature (2 K ≤ T ≤ 9 K) and high field (H_i = 50 kOe), the field was reduced to a small value (20 Oe ≤ H_{ext} ≤ 15 kOe), and the magnetisation decay to equilibrium was measured under a fixed DC field.

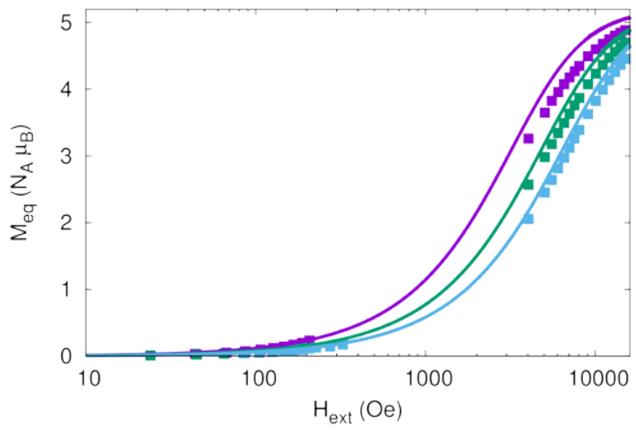

Supplementary Figure 9. M_{eq} values for **1**. Squares are fitted M_{eq} values at 2 (purple), 3 (green) and 4 K (blue), lines are theoretical values scaled to the M_{sat} value at H_i = 50 kOe.

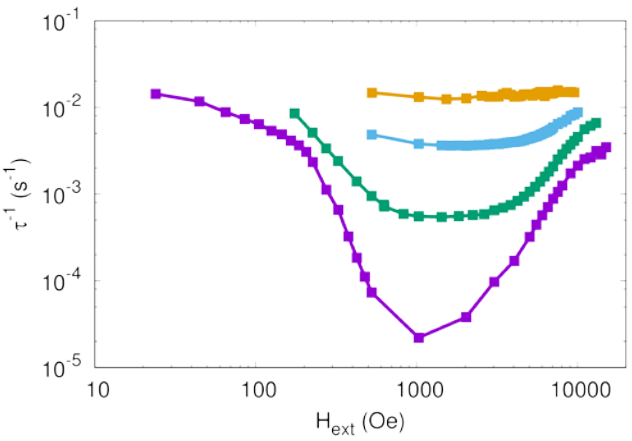

Supplementary Figure 10. Magnetic relaxation rate for **1** (note log-log scale) at 3 (purple), 5 (green), 7 (blue) and 9 K (orange). Solid lines are a guide for the eye. Error bars are within the data points.

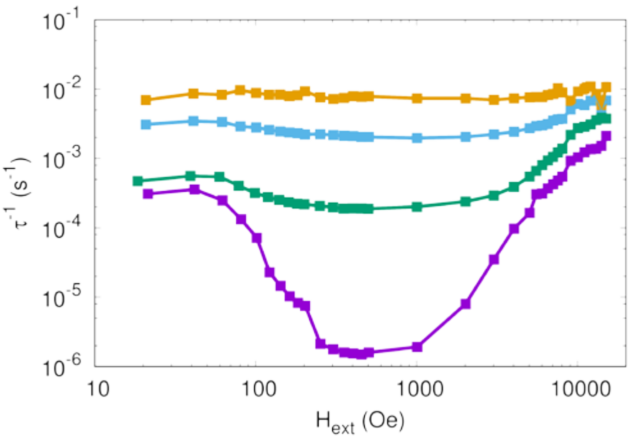

Supplementary Figure 11. Magnetic relaxation rate for **1a** (note log-log scale) at 2 (purple), 4 (green), 5 (blue) and 8 K (orange). Solid lines are a guide for the eye. Error bars are within the data points.

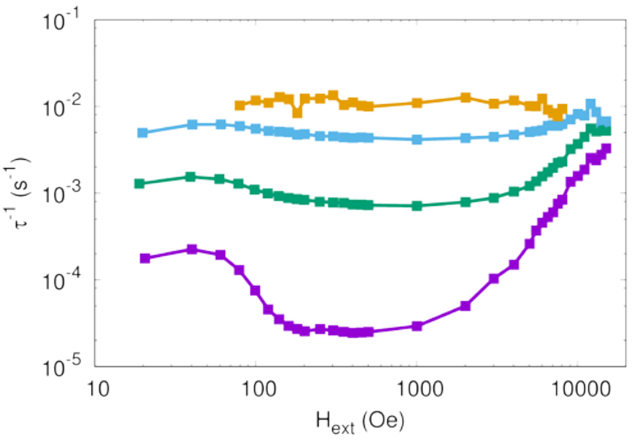

Supplementary Figure 12. Magnetic relaxation rate for **1a** (note log-log scale) at 3 (purple), 5 (green), 7 (blue) and 9 K (orange). Solid lines are a guide for the eye. Error bars are within the data points.

Supplementary Table 6. DC decay data for **1** measured at 2 K.

H_{ext} (Oe)	M_{eq} (μ_B mol^{-1})	ΔM_{eq} (μ_B mol^{-1})	τ (s)	$\Delta\tau$ (s)	α	$\Delta\alpha$	Figure
24	0.008775	0.000038	128	2	0.835	0.018	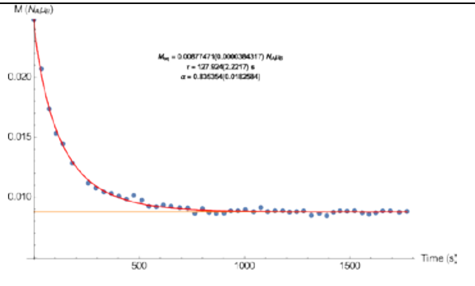
44	0.033595	0.000039	125	2	0.950	0.020	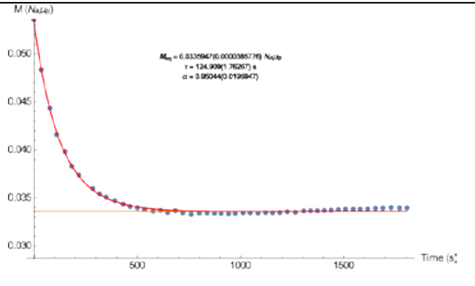
67	0.051726	0.000080	157	1	0.695	0.007	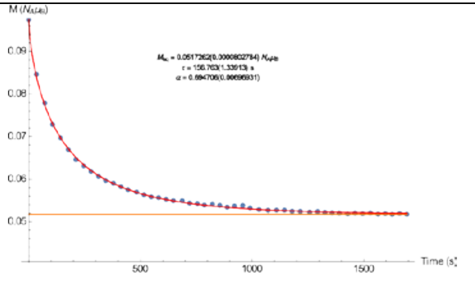
87	0.073875	0.000164	185	2	0.719	0.009	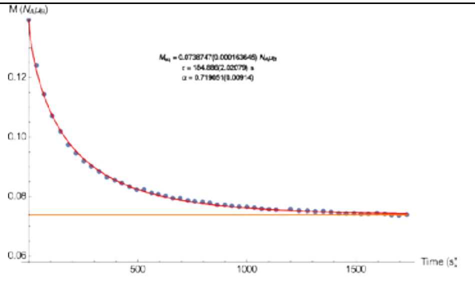
107	0.095565	0.000312	203	3	0.723	0.012	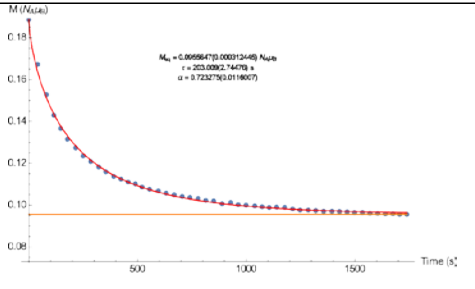
127	0.119965	0.000421	231	3	0.713	0.010	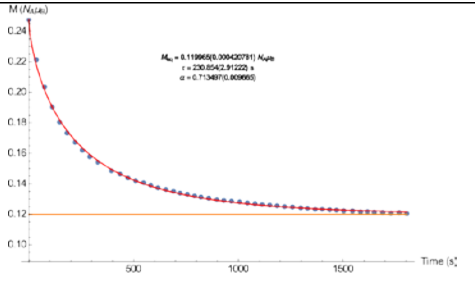

147	0.141331	0.000337	292	3	0.668	0.008	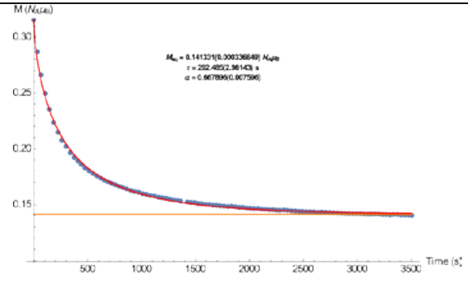
167	0.167896	0.000487	350	4	0.664	0.007	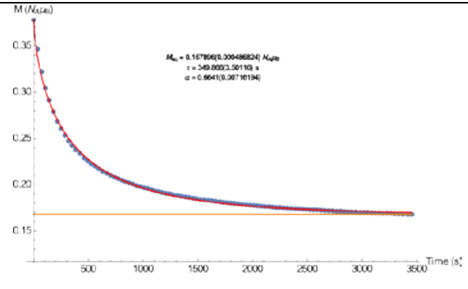
188	0.197598	0.000719	390	4	0.651	0.007	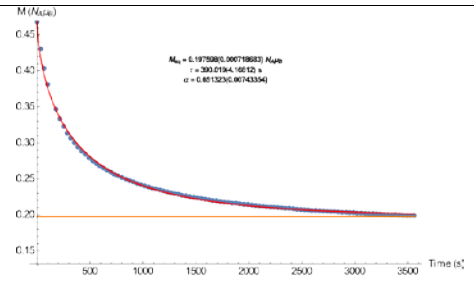
208	0.231820	0.000967	455	5	0.654	0.007	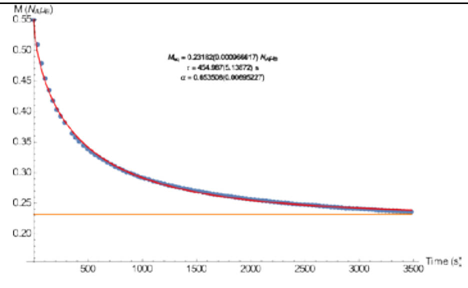
228	0.266751 [a]		544	3	0.634	0.004	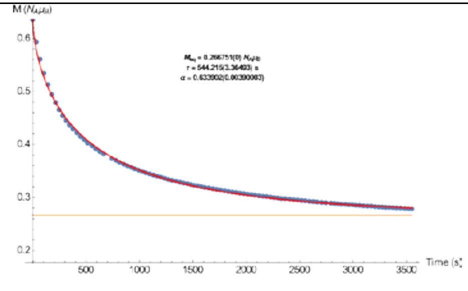
277	0.323899 [a]		902	5	0.476	0.002	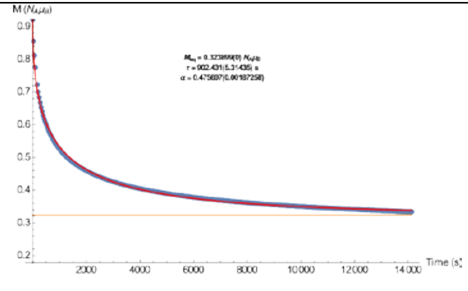

328	0.382220 [a]		1889	13	0.393	0.002	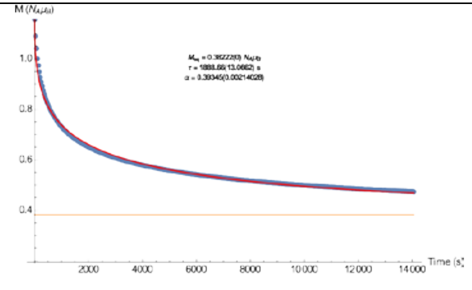
379	0.441734 [a]		4117	21	0.360	0.002	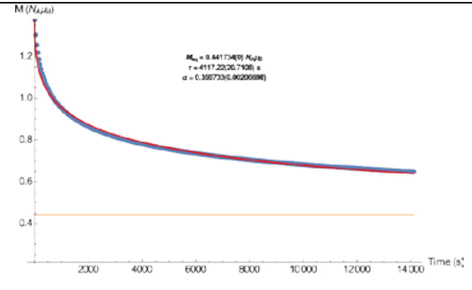
427	0.497679 [a]		8158	40	0.345	0.002	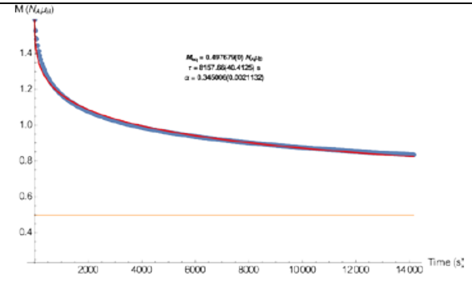
478	0.556726 [a]		15557	100	0.337	0.002	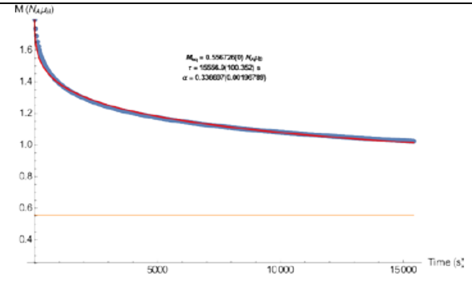
528	0.612903 [a]		26652	223	0.336	0.002	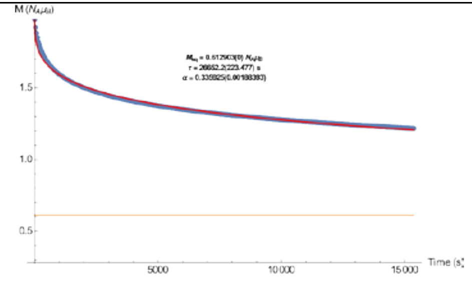
1031	1.178482 [a]		196108	843	0.410	0.001	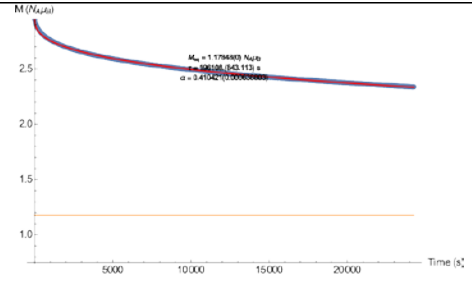

2033	2.182834 [a]		55403	53	0.561	0.000	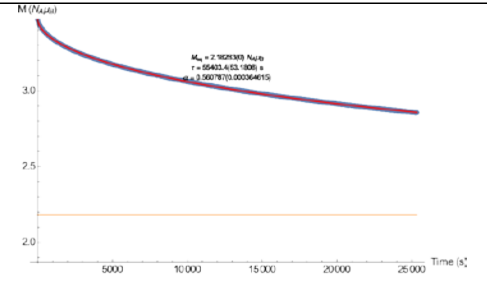
3037	2.980654 [a]		11489	24	0.797	0.003	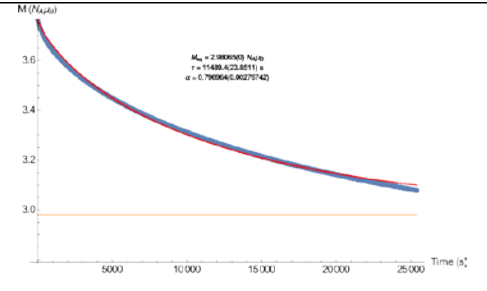
4040	3.260448	0.000601	9264	21	0.721	0.001	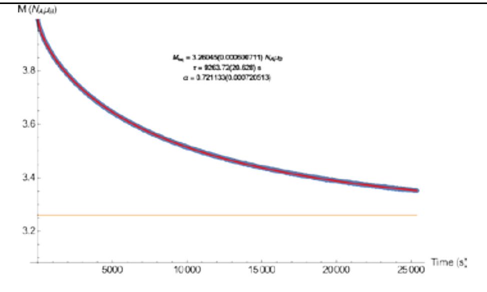
5043	3.649382	0.000376	4802	11	0.699	0.001	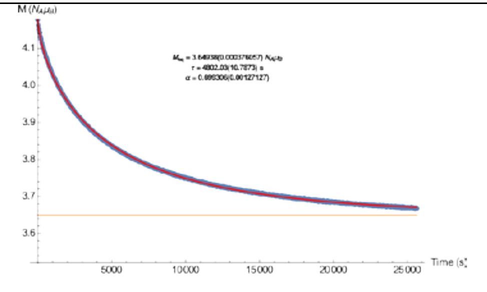
5543	3.825550	0.000743	3325	16	0.737	0.002	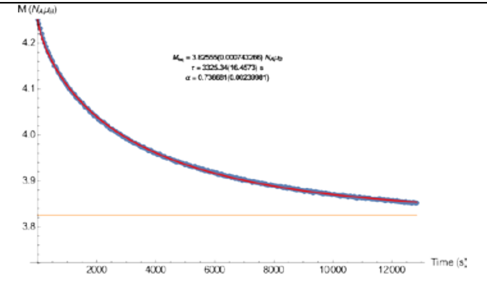
6046	3.957555	0.000583	2697	13	0.712	0.003	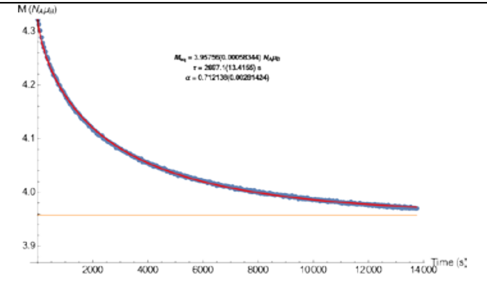

6546	4.075476	0.000506	2225	12	0.695	0.003	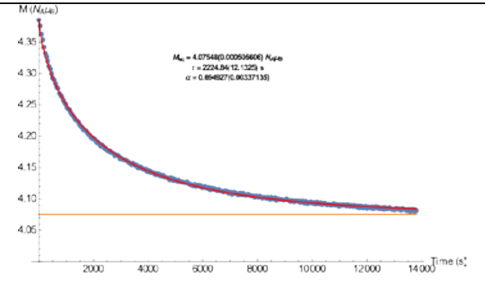
7049	4.178537	0.000355	1767	8	0.646	0.003	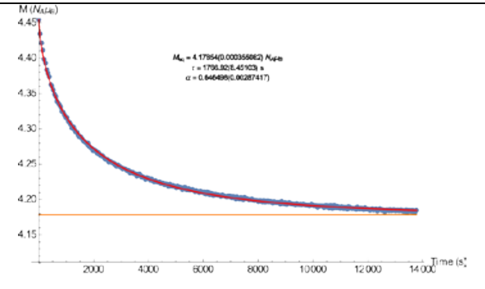
7551	4.271347	0.000326	1578	9	0.657	0.004	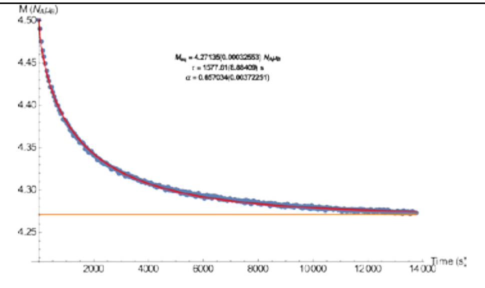
8052	4.351605	0.000289	1387	9	0.640	0.004	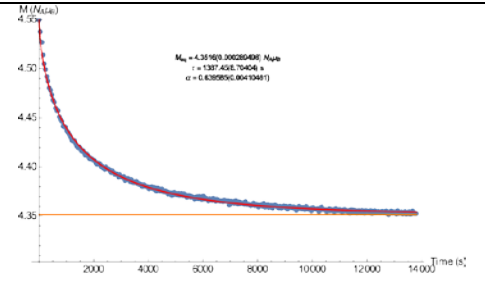
9056	4.496001	0.000941	930	19	0.719	0.012	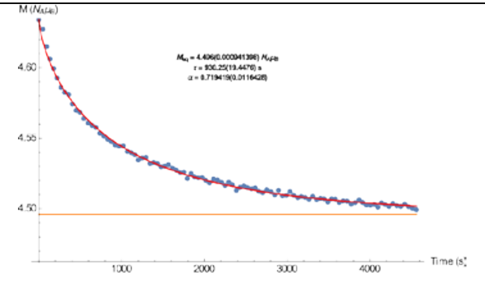
10057	4.598349	0.000641	786	15	0.684	0.011	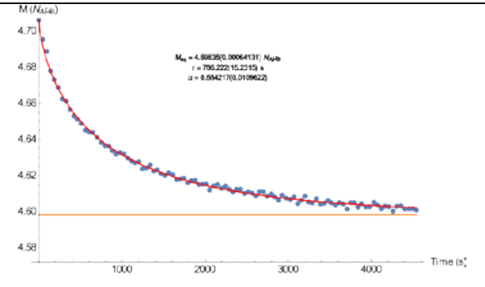

11061	4.680851	0.000596	663	15	0.658	0.013	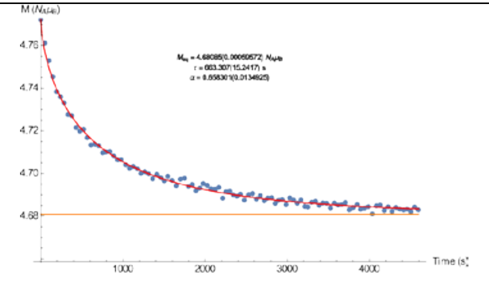
12064	4.748598	0.000464	578	14	0.675	0.016	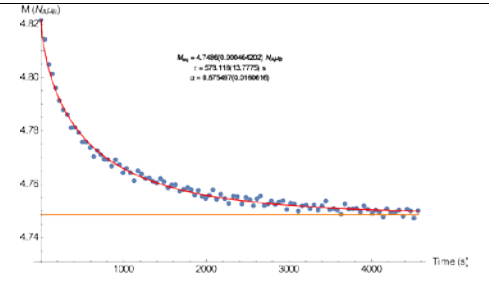
13067	4.803752	0.000397	515	14	0.693	0.020	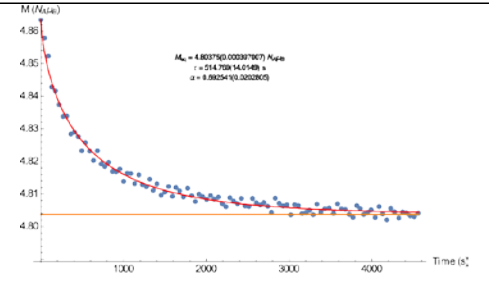
14070	4.848729	0.000370	498	15	0.713	0.025	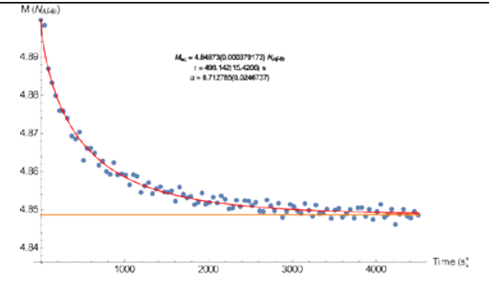
15073	4.886965	0.000260	367	11	0.664	0.022	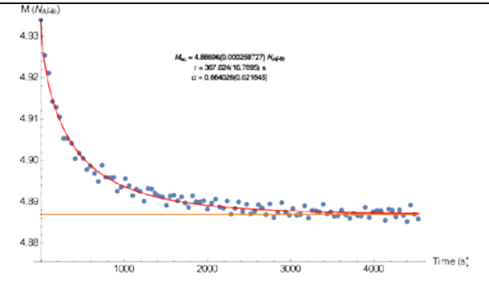

[a] M_{eq} fixed to theoretical value for $m_J = \pm15/2$ doublet scaled to the experimental M_{sat} value at H_i = 50 kOe.

Supplementary Table 7. DC decay data for **1** measured at 3 K.

H_{ext} (Oe)	M_{eq} (μ_B mol^{-1})	ΔM_{eq} (μ_B mol^{-1})	τ (s)	$\Delta\tau$ (s)	α	$\Delta\alpha$	Figure
24	0.005756	0.000028	70	4	0.823	0.061	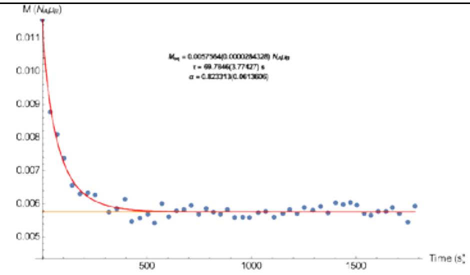
45	0.020419	0.000025	85	2	0.797	0.023	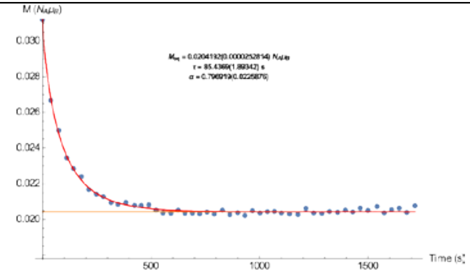
65	0.035092	0.000029	114	1	0.768	0.012	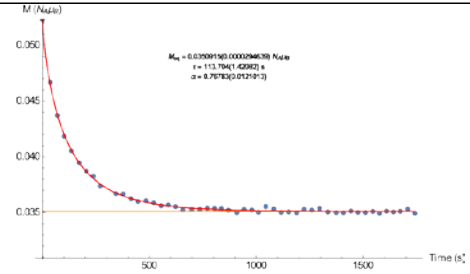
85	0.049956	0.000057	136	2	0.785	0.013	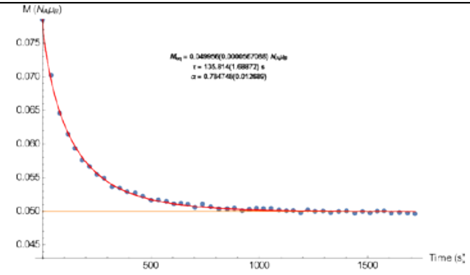
105	0.063810	0.000097	156	2	0.709	0.010	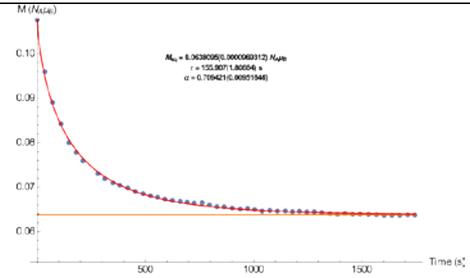
125	0.079847	0.000177	186	2	0.735	0.010	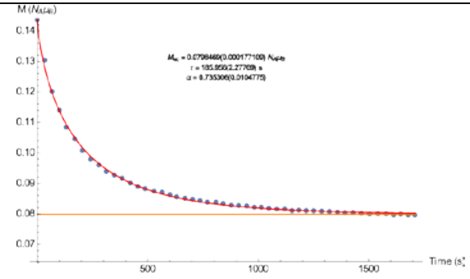

146	0.096666	0.000279	204	2	0.707	0.009	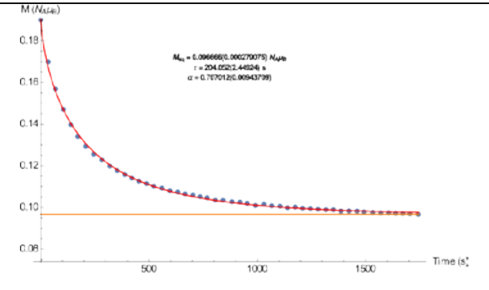
165	0.115250	0.000428	241	3	0.716	0.009	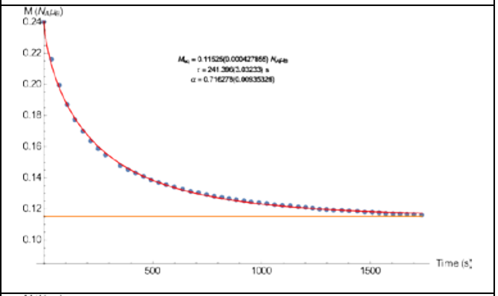
186	0.136727	0.000659	273	4	0.719	0.009	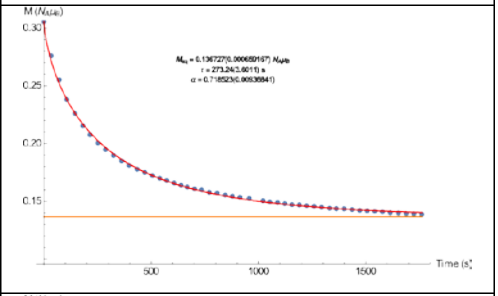
206	0.160698 [a]		325	2	0.712	0.005	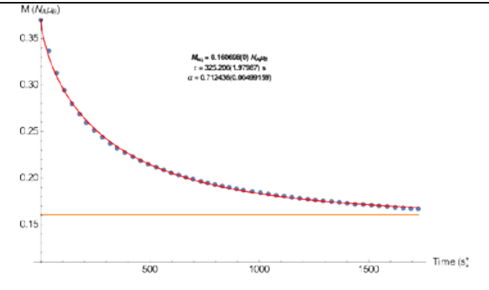
226	0.176540 [a]		426	3	0.644	0.006	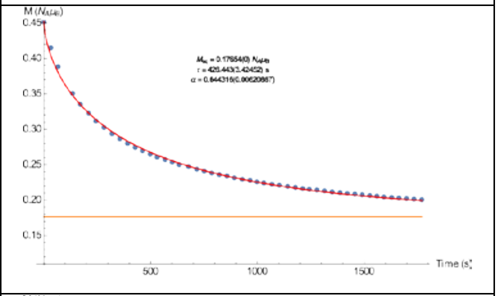
275	0.214700 [a]		893	5	0.542	0.002	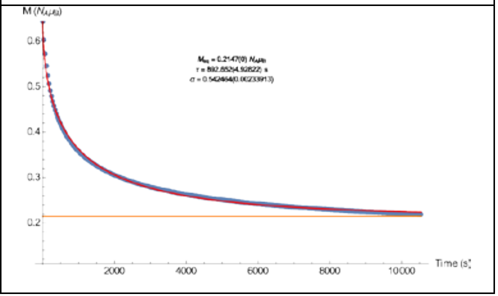

326	0.254382 [a]		1518	8	0.444	0.002	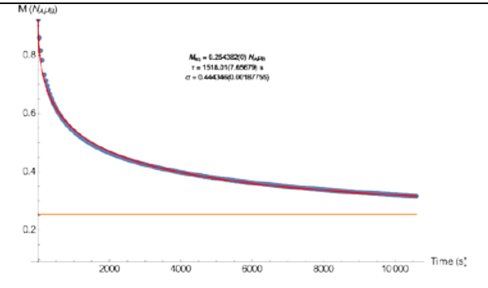
377	0.293745 [a]		3083	12	0.422	0.002	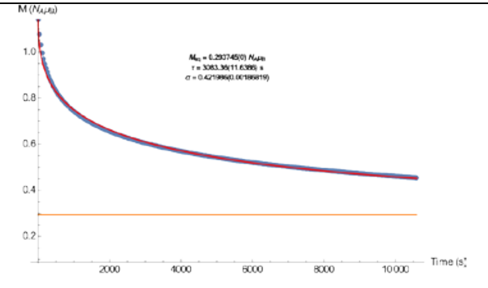
426	0.331517 [a]		5426	18	0.411	0.002	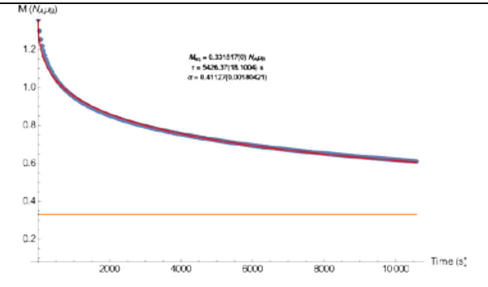
476	0.370715 [a]		9000	33	0.410	0.002	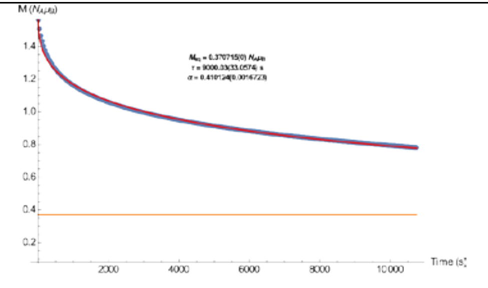
526	0.409071 [a]		13617	54	0.415	0.001	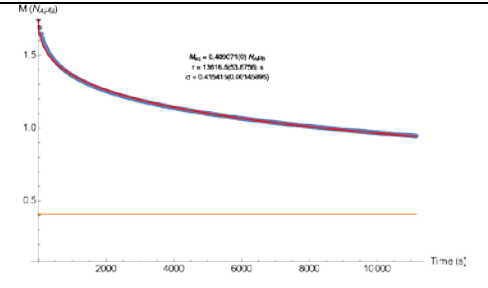
1029	0.794394 [a]		45321	45	0.539	0.000	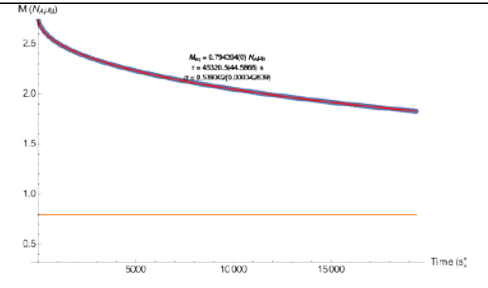

2031	1.523466 [a]		26215	14	0.633	0.000	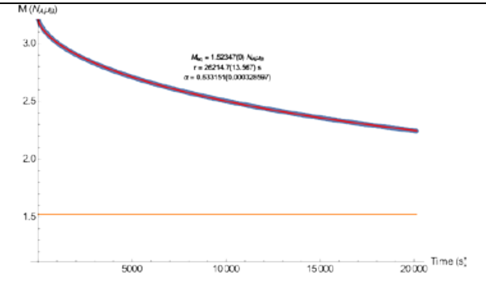
3035	2.175310 [a]		10288	8	0.749	0.001	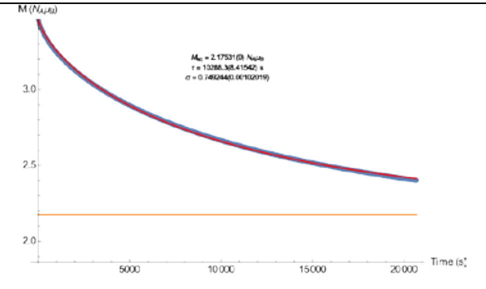
4038	2.569034	0.000633	5891	9	0.749	0.001	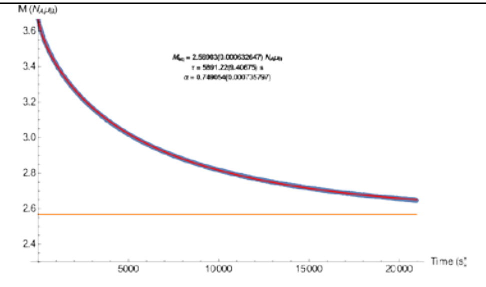
5041	2.982254	0.000497	3124	6	0.734	0.002	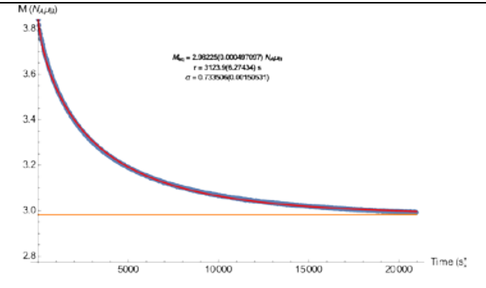
5541	3.179028	0.000720	2249	7	0.752	0.002	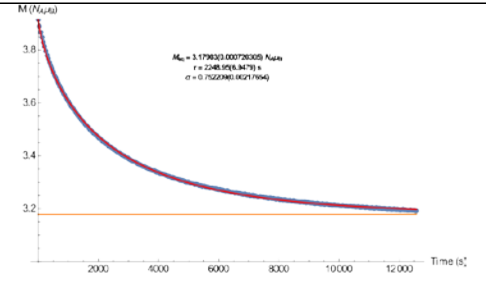
6045	3.343612	0.000605	1758	6	0.738	0.003	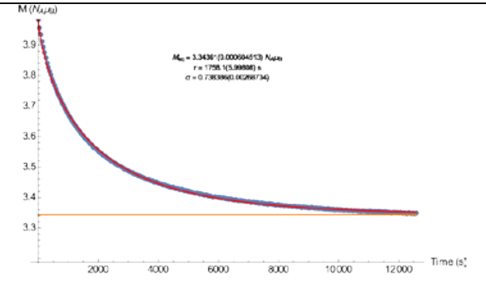

6545	3.493679	0.000490	1406	5	0.720	0.003	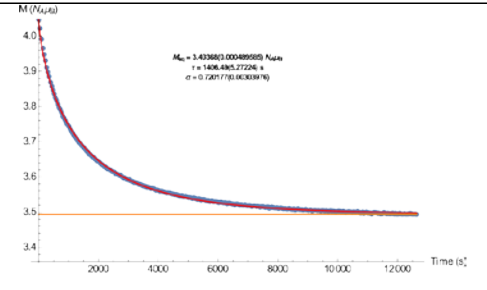
7047	3.631806	0.000374	1132	4	0.701	0.003	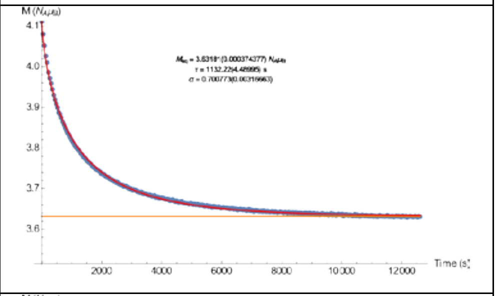
7549	3.757347	0.000311	942	4	0.687	0.004	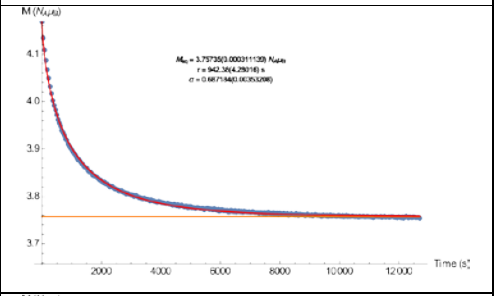
8050	3.871047	0.000262	797	4	0.673	0.004	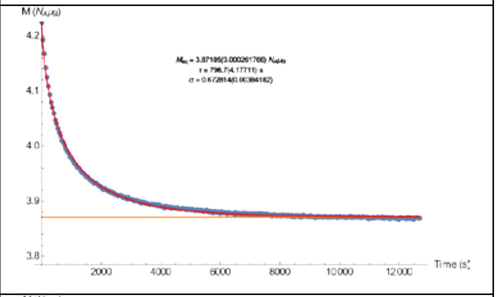
9053	4.074979	0.000667	576	6	0.725	0.008	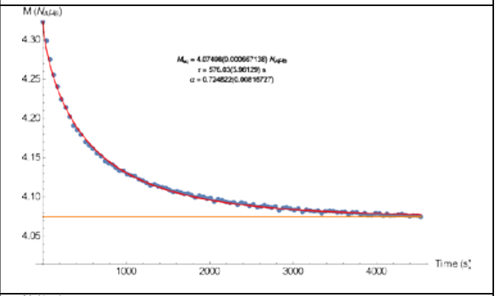
10056	4.232951	0.000493	469	6	0.695	0.009	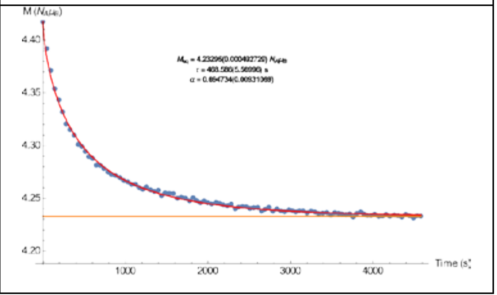

11058	4.363189	0.000380	395	5	0.659	0.010	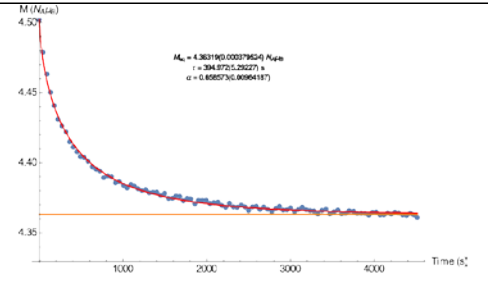
12062	4.471210	0.000367	378	7	0.658	0.013	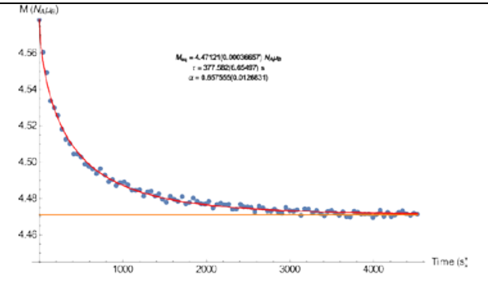
13064	4.562220	0.000258	318	6	0.686	0.015	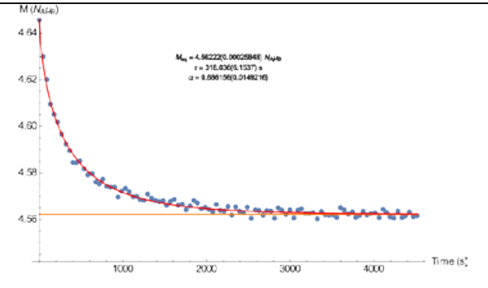
14067	4.637039	0.000257	349	9	0.761	0.024	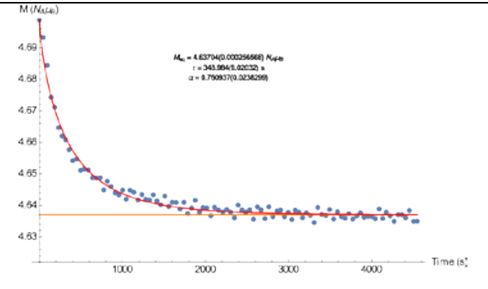
15071	4.699944	0.000270	288	9	0.668	0.024	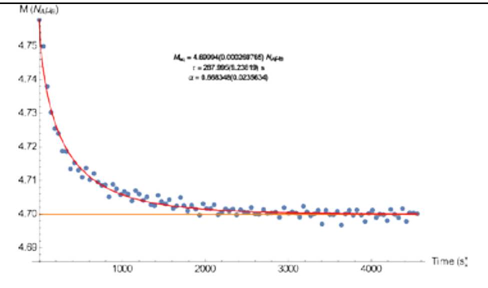

[a] M_{eq} fixed to theoretical value for $m_J = \pm 15/2$ doublet scaled to the experimental M_{sat} value at $H_i = 50$ kOe.

Supplementary Table 8. DC decay data for **1** measured at 4 K.

H H_{ext} (Oe)	M_{eq} (μ_B mol^{-1})	ΔM_{eq} (μ_B mol^{-1})	τ (s)	$\Delta\tau$ (s)	α	$\Delta\alpha$	Figure
86	0.038001	0.000026	91	2	0.847	0.024	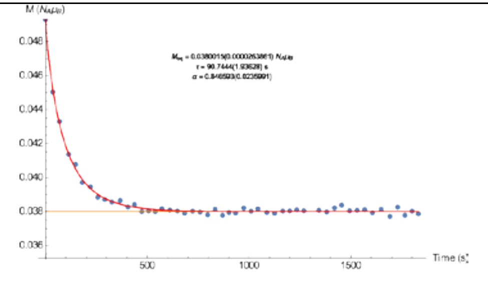
105	0.048377	0.000031	110	1	0.818	0.013	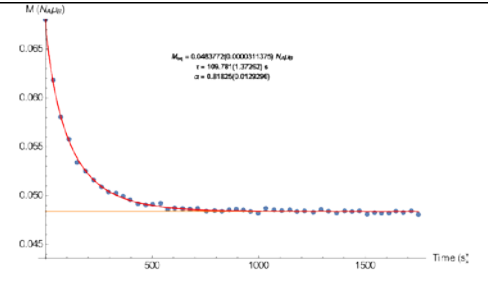
125	0.059601	0.000056	130	1	0.759	0.011	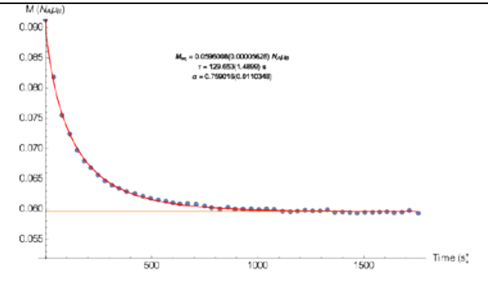
145	0.071054	0.000094	152	2	0.719	0.009	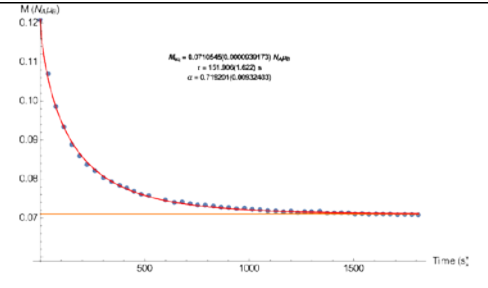
166	0.083233	0.000195	180	2	0.717	0.010	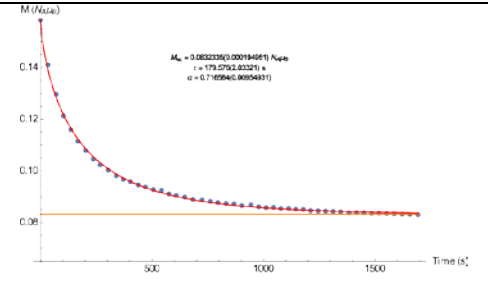
186	0.096390	0.000327	211	2	0.732	0.009	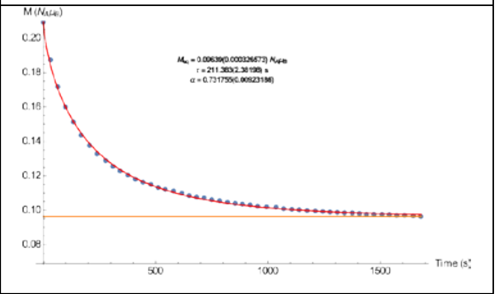

206	0.110039	0.000381	249	2	0.704	0.006	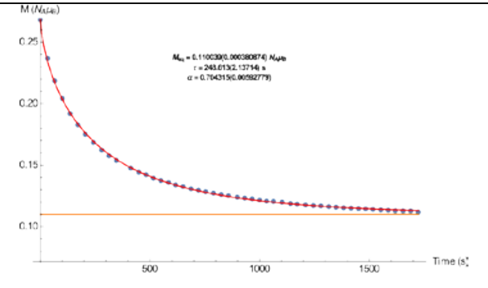
227	0.126752	0.000810	307	4	0.728	0.008	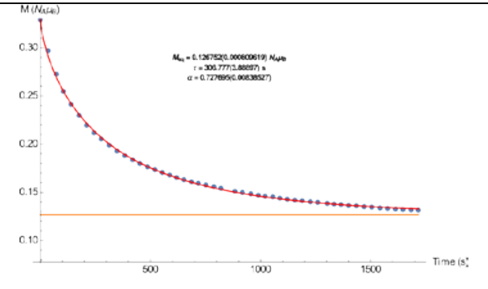
275	0.143299	0.000206	636	2	0.618	0.002	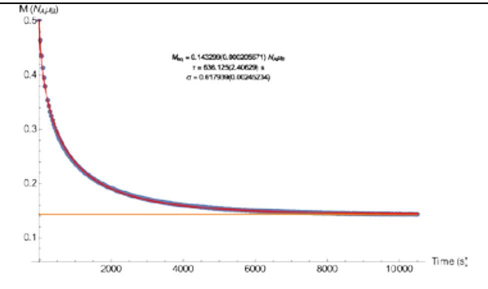
326	0.169555	0.000448	939	3	0.565	0.002	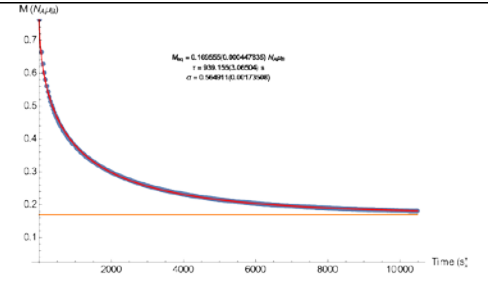
377	0.220364 [a]		1377	5	0.602	0.002	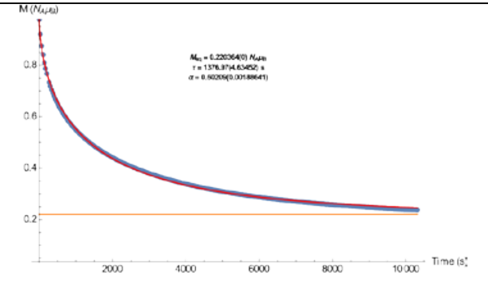
426	0.249096 [a]		2046	4	0.589	0.001	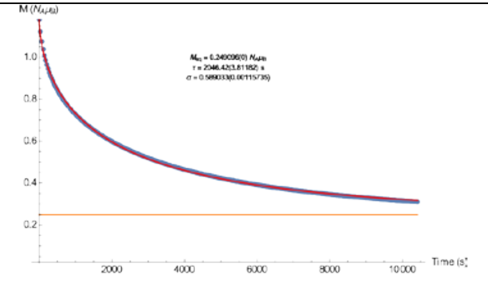

476	0.278666 [a]		2830	3	0.588	0.001	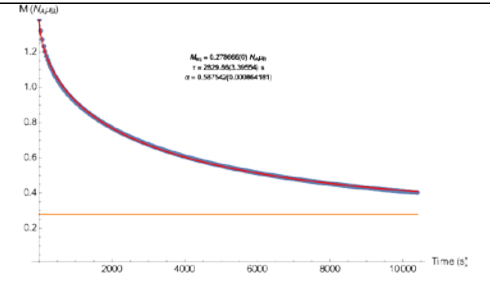
525	0.307285 [a]		3662	3	0.597	0.001	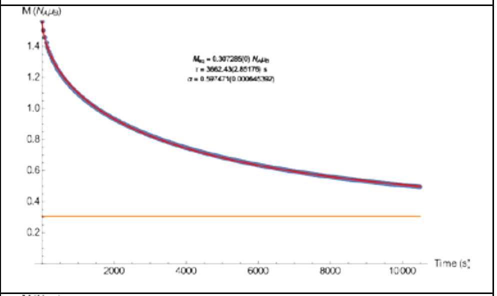
1029	0.599023 [a]		7730	2	0.708	0.000	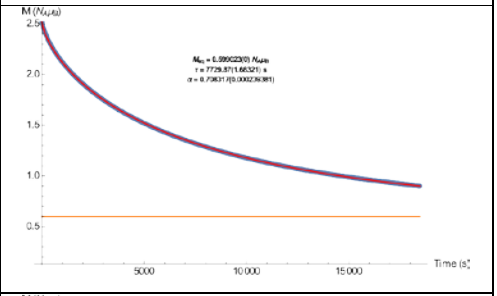
2031	1.163043 [a]		6682	2	0.749	0.000	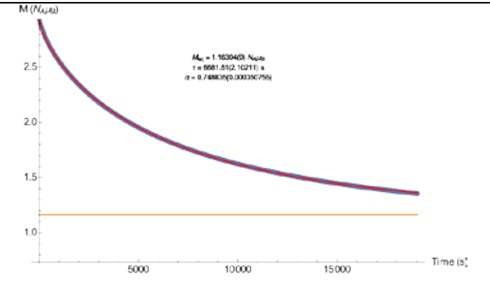
3035	1.693468 [a]		4392	10	0.833	0.003	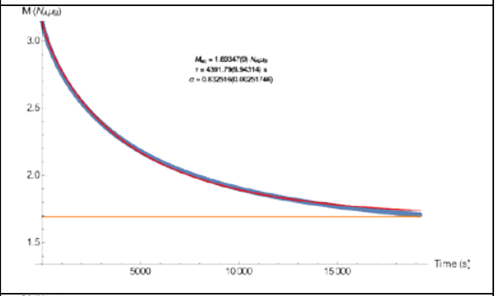
4038	2.052913	0.000532	3208	4	0.770	0.001	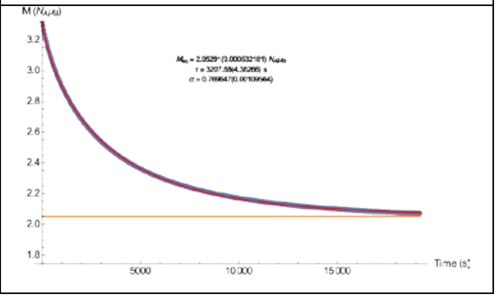

5041	2.450060	0.000397	1951	4	0.758	0.002	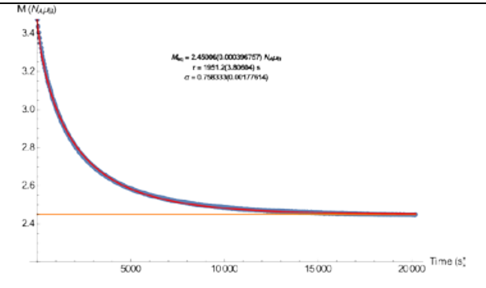
5541	2.640791	0.000566	1483	4	0.767	0.002	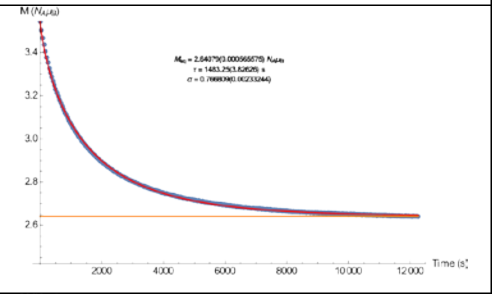
6044	2.811661	0.000495	1184	4	0.762	0.003	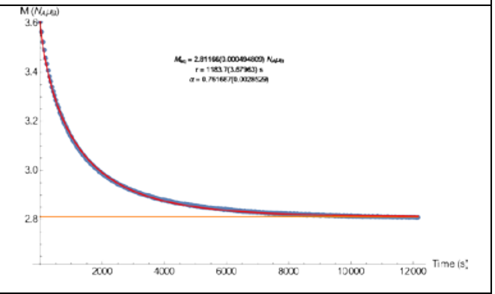
6544	2.970416	0.000379	951	3	0.746	0.003	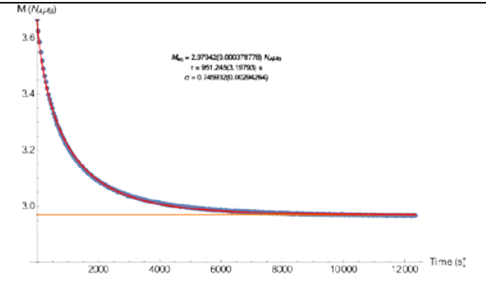
7047	3.120520	0.000338	778	3	0.740	0.004	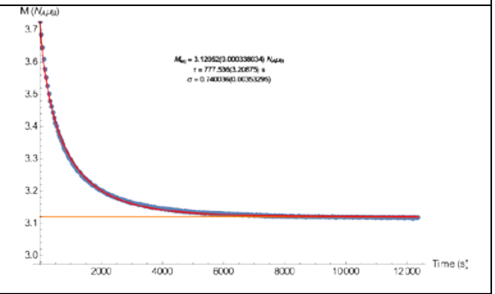
7549	3.260101	0.000280	641	3	0.727	0.004	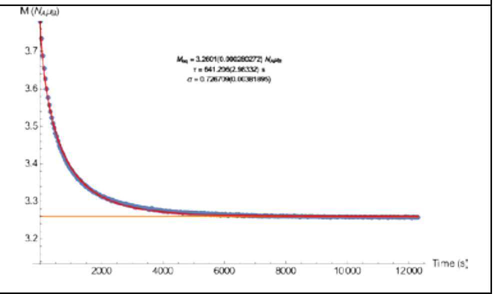

8050	3.389725	0.000236	537	3	0.715	0.004	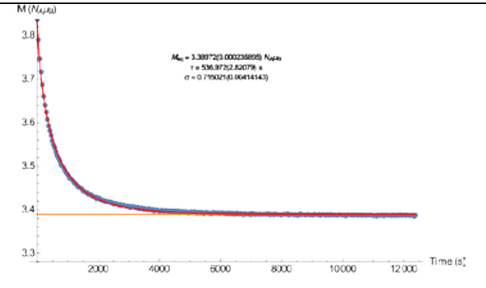
9054	3.626821	0.000518	383	3	0.733	0.007	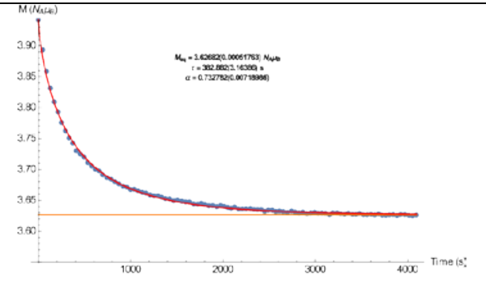
10056	3.823426	0.000362	314	3	0.733	0.009	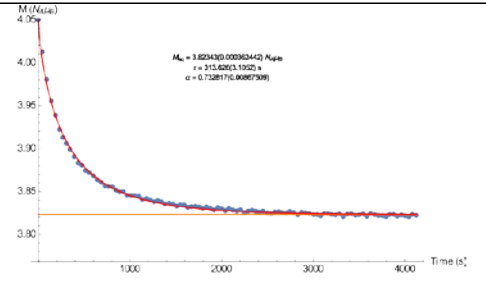
11058	3.991497	0.000234	265	3	0.702	0.009	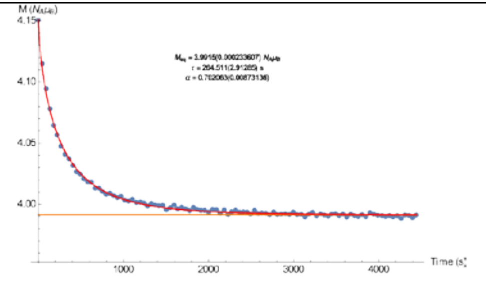
12062	4.135975	0.000232	243	4	0.710	0.013	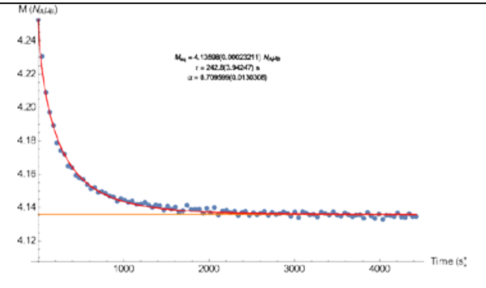
13065	4.259156	0.000200	242	5	0.734	0.017	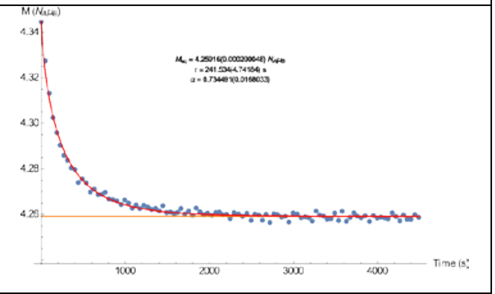

14067	4.364698	0.000183	210	5	0.676	0.018	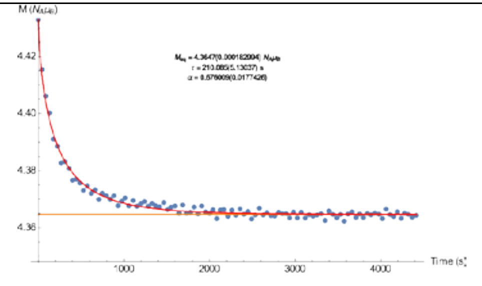
15070	4.455650	0.000163	206	6	0.779	0.028	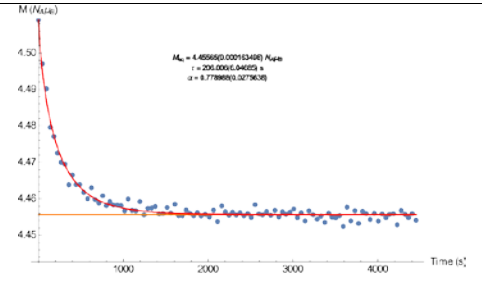

[a] M_{eq} fixed to theoretical value for m_J = ±15/2 doublet scaled to the experimental M_{sat} value at H_i = 50 kOe.

Supplementary Table 9. DC decay data for **1** measured at 5 K.

H_{ext} (Oe)	M_{eq} (μ_B mol^{-1})	ΔM_{eq} (μ_B mol^{-1})	τ (s)	$\Delta\tau$ (s)	α	$\Delta\alpha$	Figure
175	0.069416	0.000047	117	1	0.821	0.007	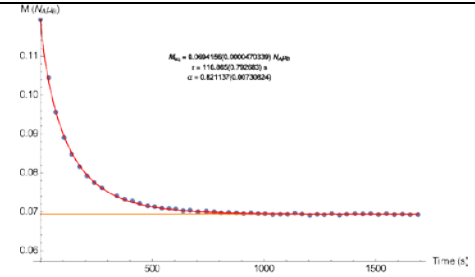
225	0.091932	0.000125	195	1	0.793	0.004	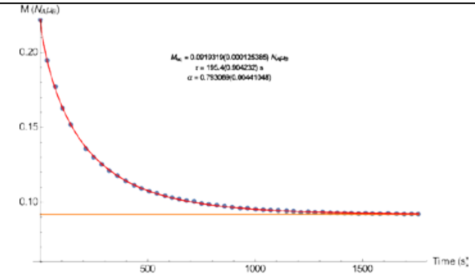
274	0.116233	0.000444	298	2	0.806	0.005	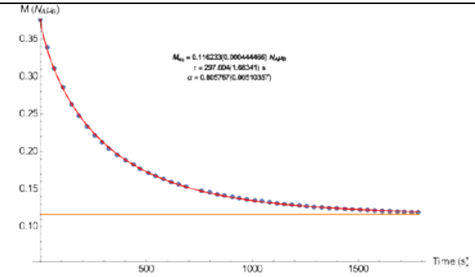
324	0.151968 [a]		414	1	0.824	0.004	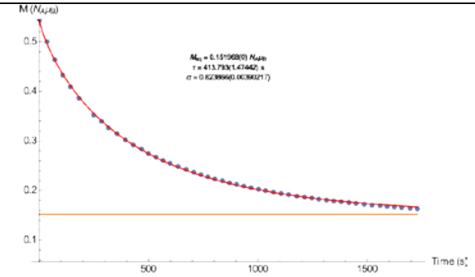
424	0.198725 [a]		712	1	0.747	0.002	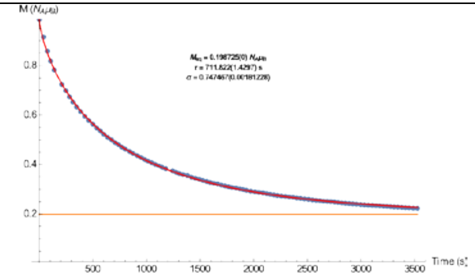
524	0.245481 [a]		1054	1	0.751	0.001	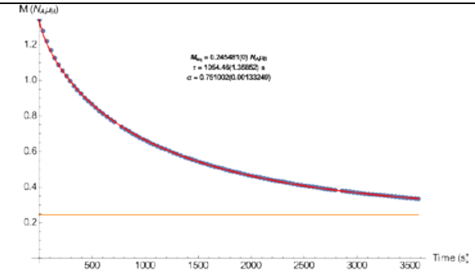

626	0.293031 [a]		1325	1	0.768	0.001	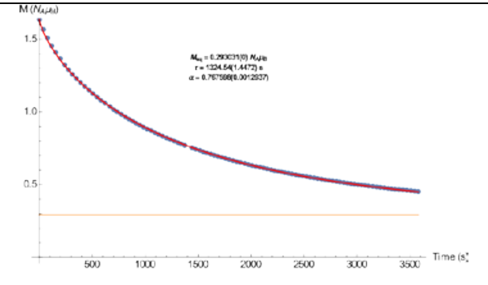
630	0.269740	0.000322	1387	2	0.743	0.001	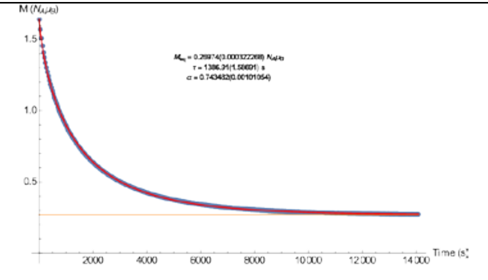
829	0.360080	0.000471	1692	2	0.775	0.001	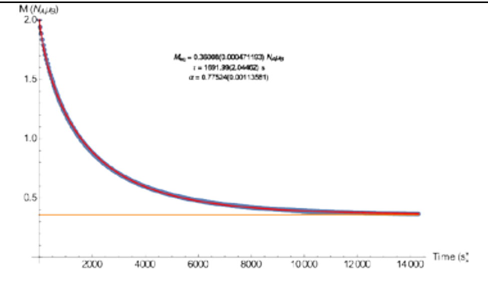
1030	0.449226	0.000565	1807	2	0.790	0.001	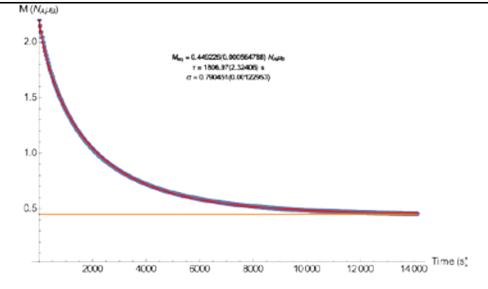
1431	0.630399	0.000997	1835	3	0.809	0.002	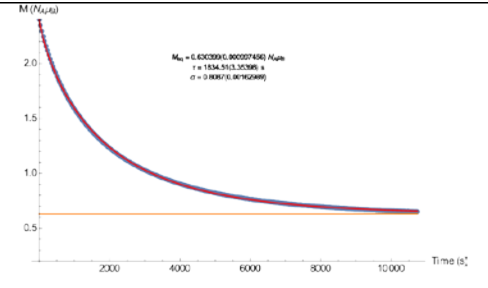
1831	0.796750	0.000896	1799	3	0.802	0.002	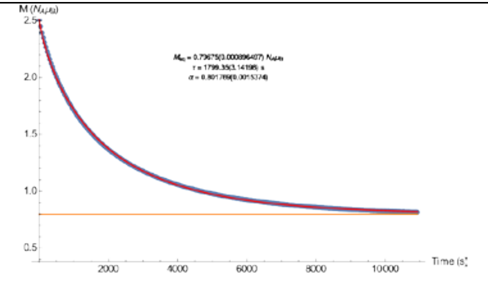

2233	0.962320	0.000774	1746	3	0.802	0.001	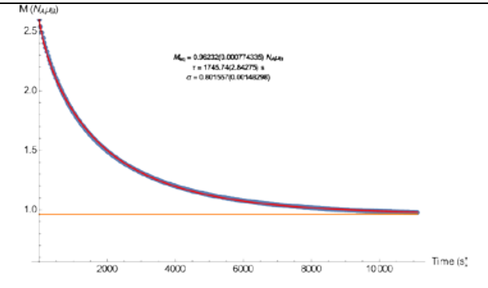
2635	1.115252	0.001108	1703	4	0.796	0.002	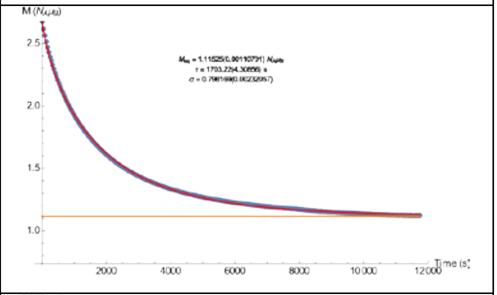
3036	1.283503	0.000669	1540	3	0.802	0.002	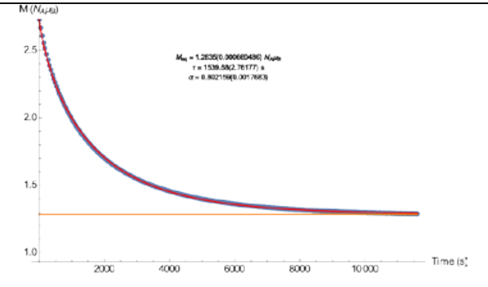
3437	1.442687	0.000540	1435	2	0.804	0.002	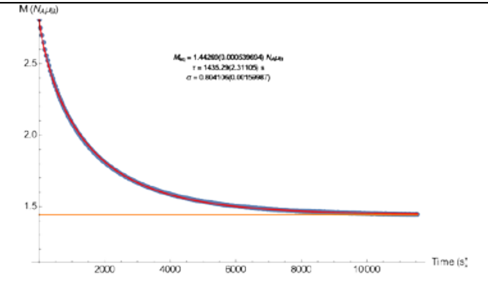
3839	1.595381	0.000475	1339	2	0.807	0.002	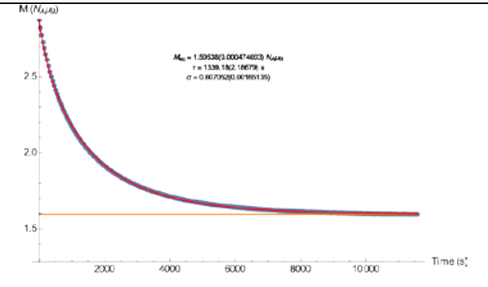
4239	1.744405	0.000414	1199	2	0.809	0.002	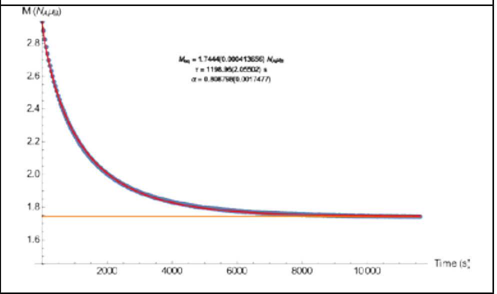

4642	1.889774	0.000371	1065	2	0.807	0.002	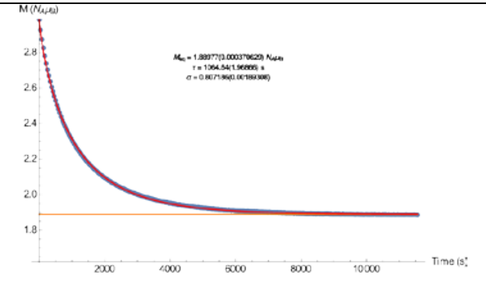
5042	2.029969	0.000341	936	2	0.804	0.002	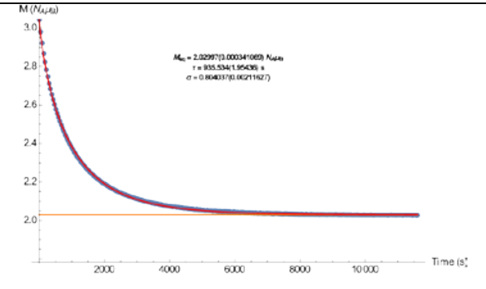
5443	2.165511	0.000305	821	2	0.801	0.002	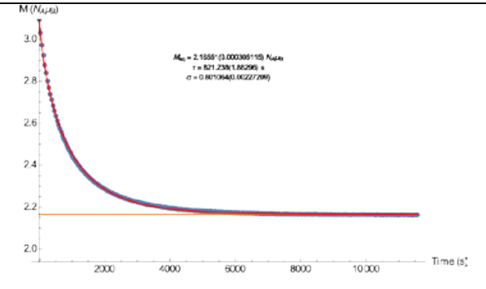
5844	2.296811	0.000285	718	2	0.800	0.003	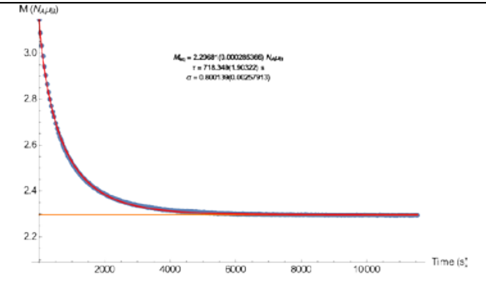
6245	2.423021	0.000257	625	2	0.794	0.003	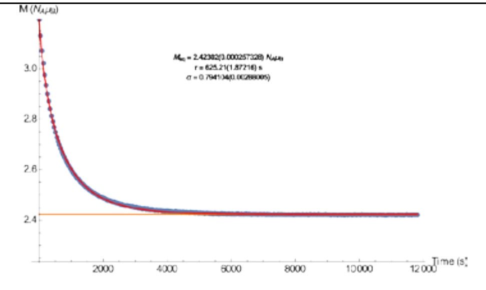
6646	2.544741	0.000221	551	2	0.789	0.003	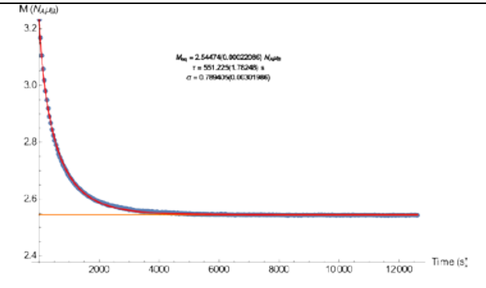

7048	2.661917	0.000193	479	2	0.785	0.003	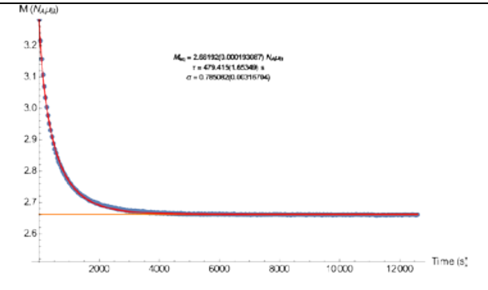
7534	2.806060	0.000511	399	2	0.801	0.005	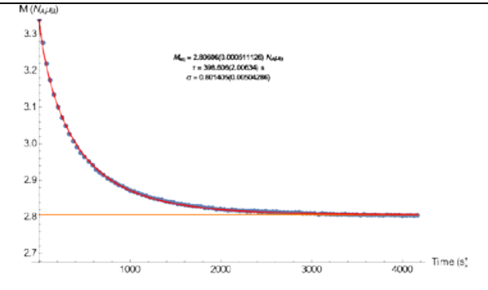
8034	2.937042	0.000517	348	2	0.800	0.007	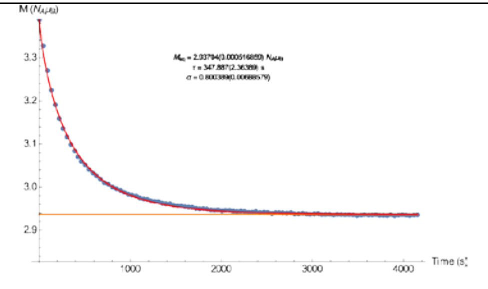
8535	3.060965	0.000356	302	2	0.785	0.006	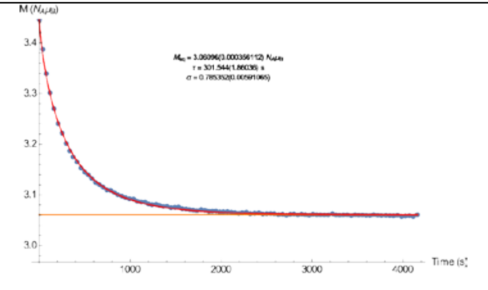
9038	3.179054	0.000260	270	2	0.777	0.006	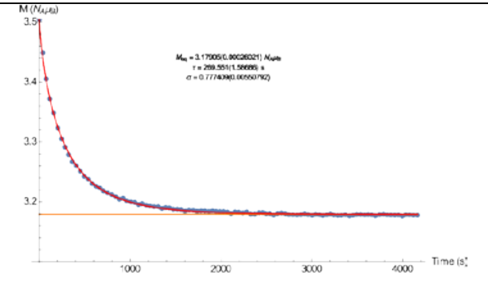
9538	3.289811	0.000308	243	2	0.764	0.009	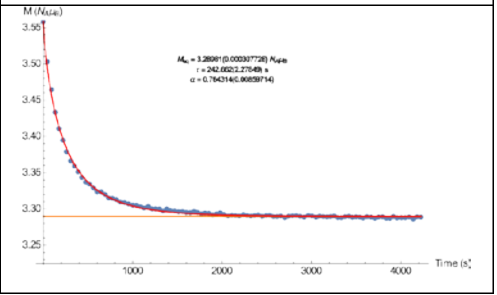

10040	3.394249	0.000238	219	2	0.766	0.008	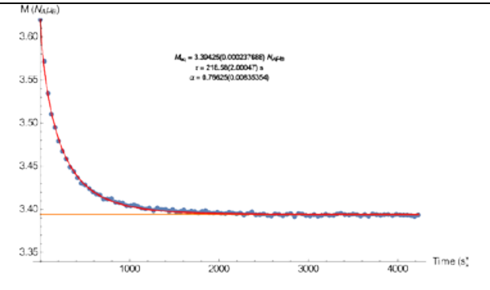
11042	3.585894	0.000367	178	2	0.728	0.011	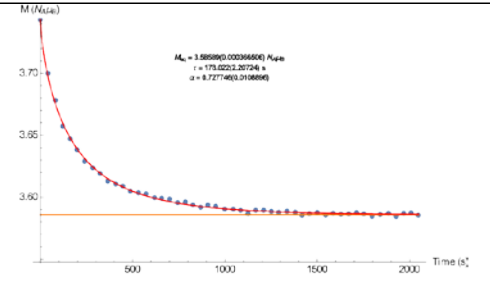
12045	3.755090	0.000327	163	3	0.785	0.019	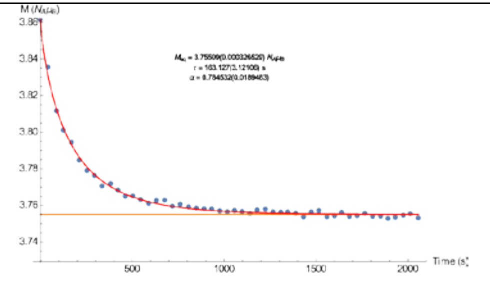
13047	3.903634	0.000288	151	3	0.716	0.020	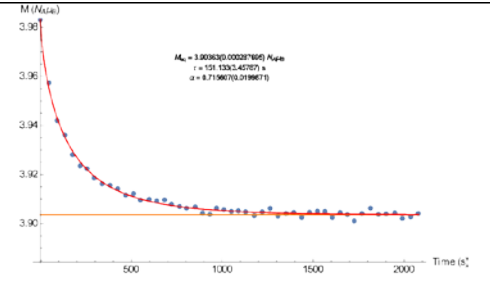

[a] M_{eq} fixed to theoretical value for $m_J = \pm 15/2$ doublet scaled to the experimental M_{sat} value at H_i = 50 kOe.

Supplementary Table 10. DC decay data for **1** measured at 6 K.

H_{ext} (Oe)	M_{eq} (μ_B mol^{-1})	ΔM_{eq} (μ_B mol^{-1})	τ (s)	$\Delta\tau$ (s)	α	$\Delta\alpha$	Figure
228	0.077368	0.000040	111.5	0.4	0.881	0.005	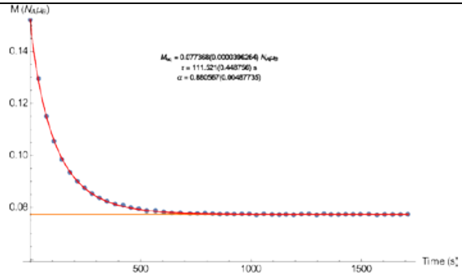
327	0.115497	0.000230	225.8	0.8	0.857	0.004	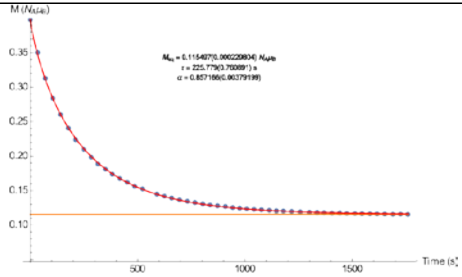
427	0.157951	0.000807	331.8	1.3	0.831	0.004	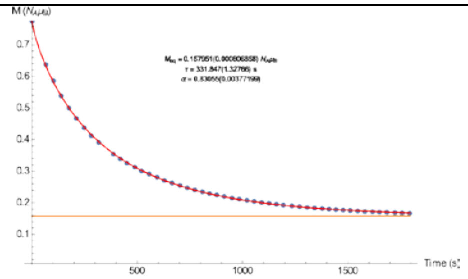
527	0.205909 [a]		423.4	0.7	0.844	0.002	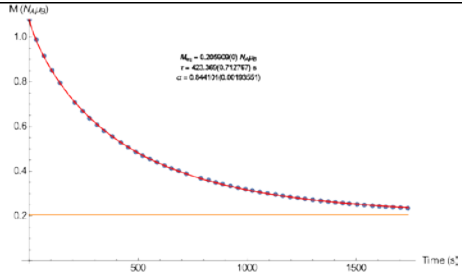
629	0.245485 [a]		501.3	1.2	0.857	0.003	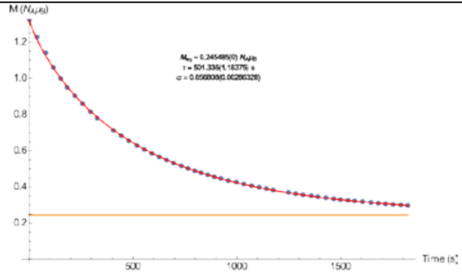
829	0.310330	0.000972	658.6	2.0	0.898	0.003	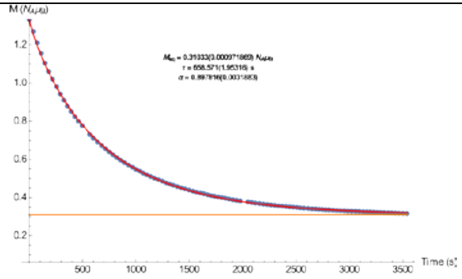

1029	0.387648	0.001516	619.7	2.1	0.869	0.003	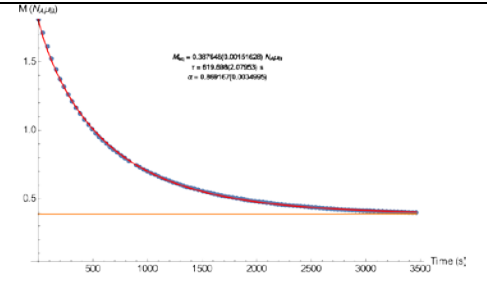
1230	0.460729	0.001287	630.0	1.8	0.869	0.003	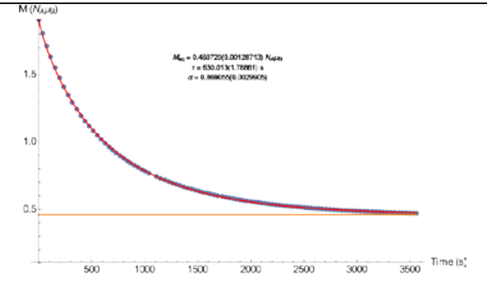
1430	0.534970	0.001449	631.5	2.0	0.876	0.003	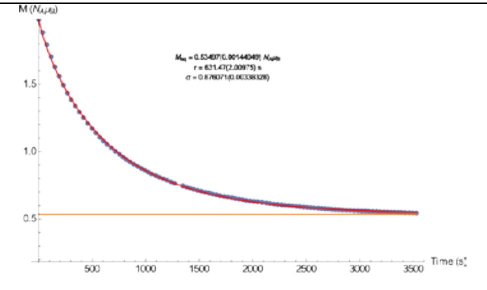
1632	0.604372	0.001121	631.0	1.6	0.863	0.003	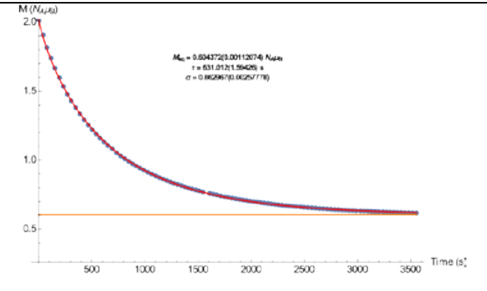
1830	0.677877	0.001236	627.9	1.8	0.874	0.003	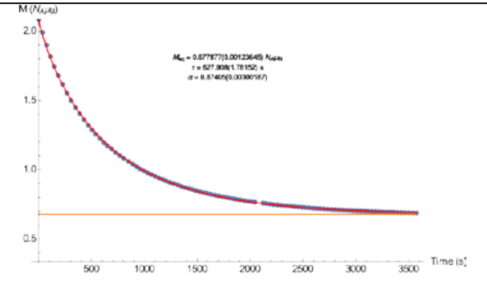
2031	0.748629	0.001362	627.2	2.0	0.873	0.003	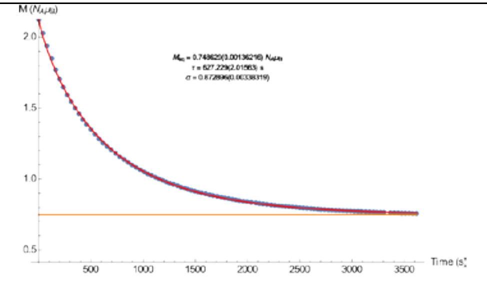

2231	0.819991	0.001146	617.0	1.7	0.872	0.003	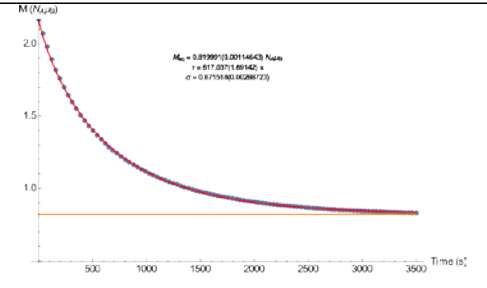
2433	0.891322	0.001182	610.0	1.8	0.872	0.003	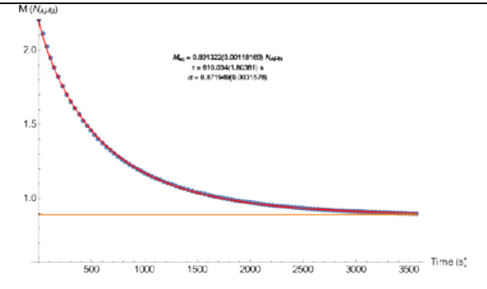
2634	0.959478	0.000943	605.9	1.5	0.869	0.003	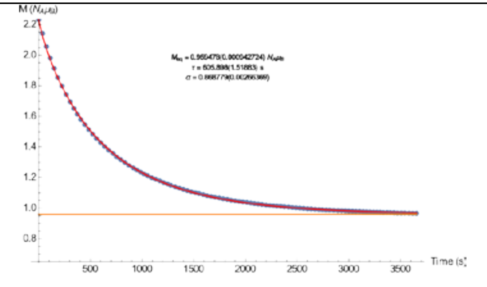
2834	1.028907	0.000988	593.6	1.6	0.863	0.003	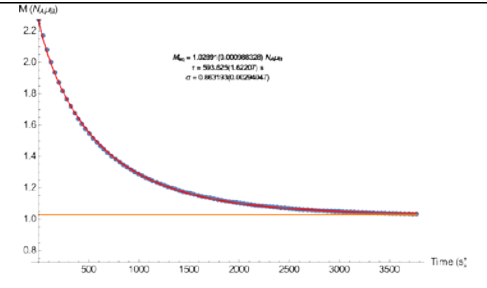
3035	1.098800	0.001030	588.4	1.8	0.869	0.003	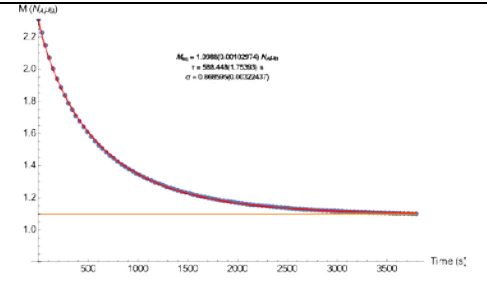
3235	1.165859	0.000962	580.5	1.7	0.865	0.003	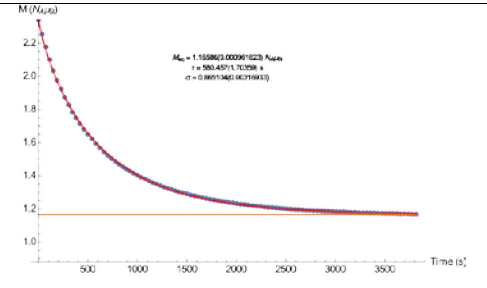

3436	1.234441	0.000842	567.1	1.5	0.866	0.003	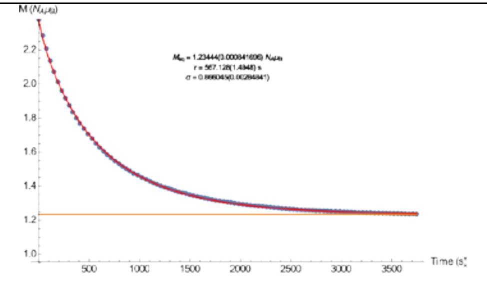
3638	1.302305	0.000882	553.5	1.6	0.863	0.003	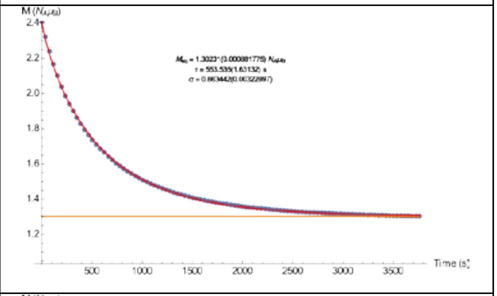
3838	1.368131	0.000744	540.6	1.4	0.858	0.003	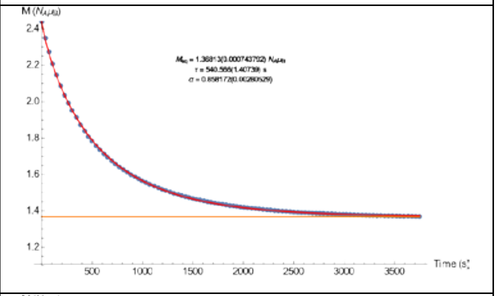
4038	1.433970	0.000675	528.6	1.3	0.859	0.003	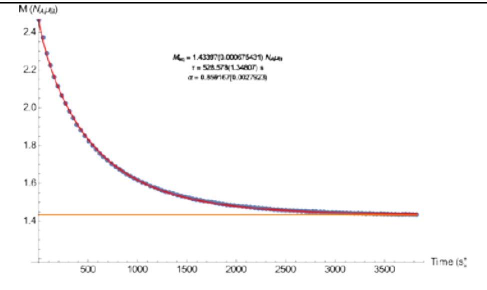
4238	1.499401	0.000726	511.8	1.5	0.859	0.003	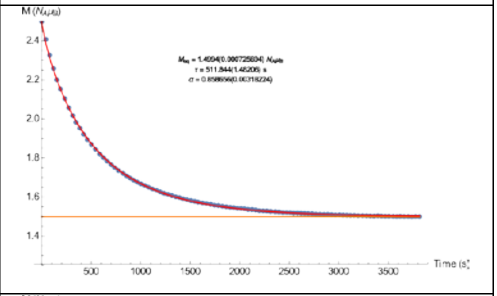
4440	1.564714	0.000583	496.9	1.2	0.861	0.003	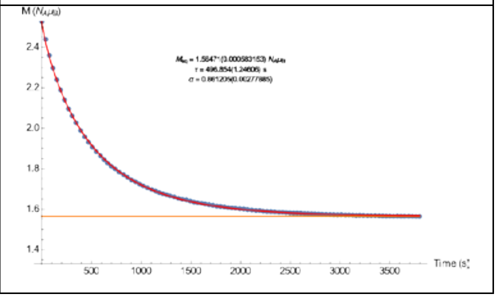

4641	1.628999	0.000598	480.4	1.3	0.859	0.003	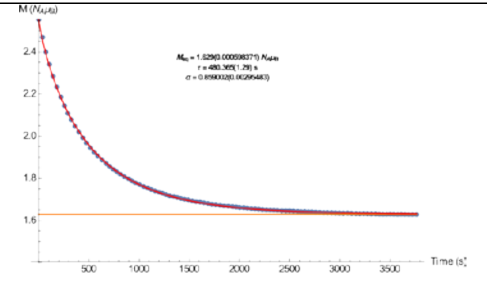
4840	1.692046	0.000600	465.3	1.3	0.860	0.003	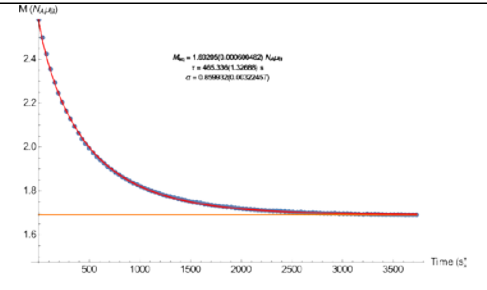
5042	1.754897	0.000610	447.0	1.4	0.860	0.004	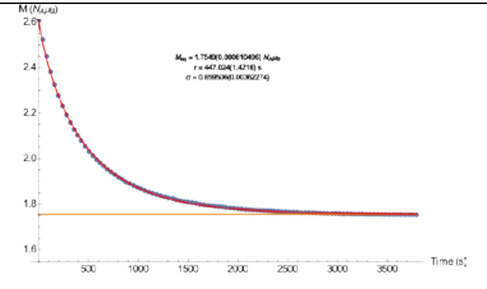
5242	1.816592	0.000551	432.1	1.3	0.861	0.004	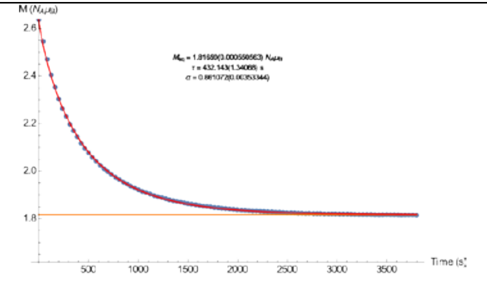
5442	1.877251	0.000521	409.5	1.3	0.848	0.004	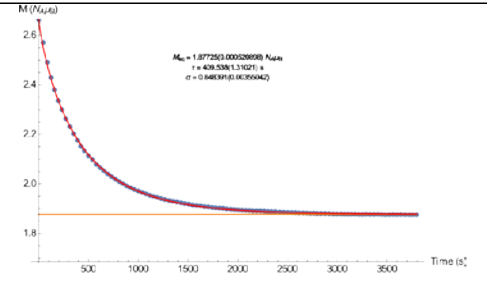
5643	1.937586	0.000427	396.7	1.1	0.859	0.003	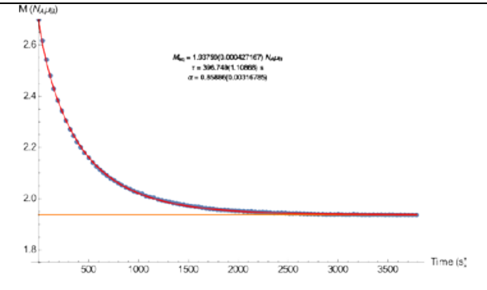

5843	1.997447	0.000493	378.0	1.3	0.856	0.004	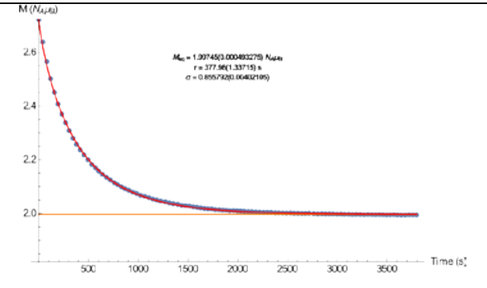
6045	2.055783	0.000434	359.3	1.2	0.840	0.004	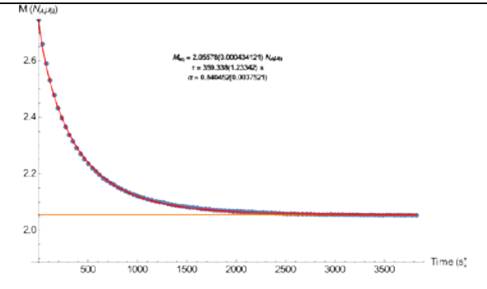
6244	2.113583	0.000447	350.8	1.4	0.855	0.004	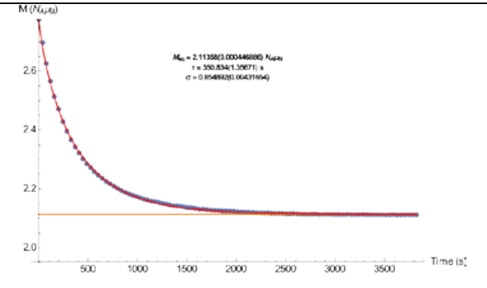
6445	2.171025	0.000358	331.0	1.1	0.852	0.004	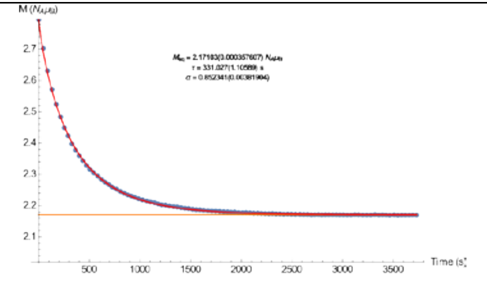
6645	2.227234	0.000391	314.5	1.3	0.850	0.005	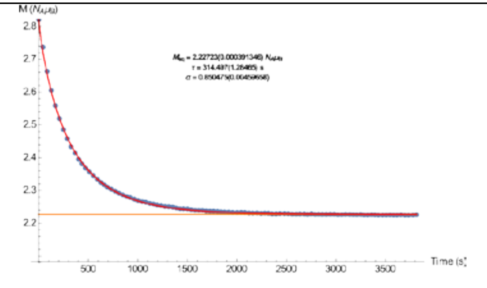
6846	2.282475	0.000353	297.0	1.2	0.833	0.004	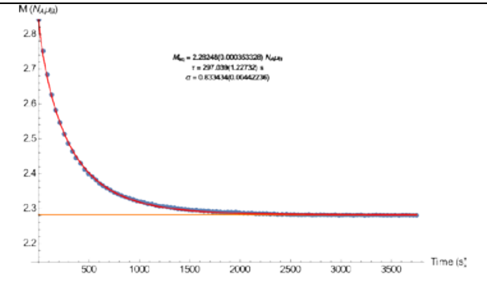

7047	2.336813	0.000396	289.6	1.4	0.840	0.005	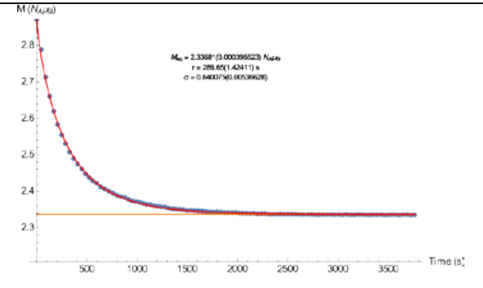
7534	2.471828	0.000713	251.2	1.6	0.846	0.007	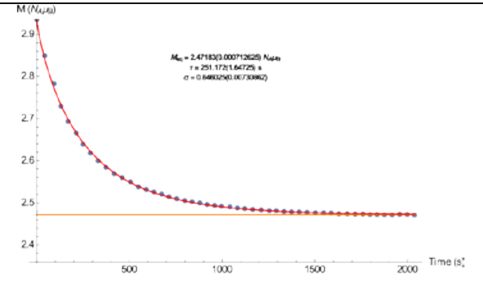
8034	2.599255	0.000535	226.2	1.5	0.839	0.007	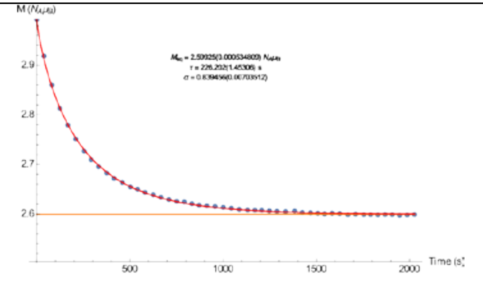
8534	2.720779	0.000545	204.1	1.7	0.841	0.009	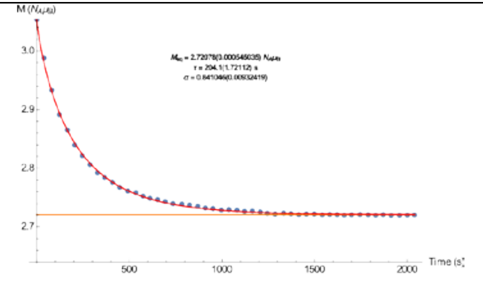
9035	2.838219	0.000478	188.6	1.8	0.849	0.011	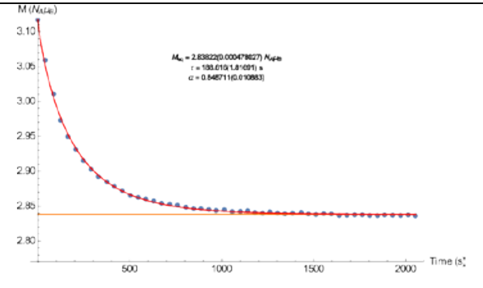
9537	2.949090	0.000357	167.8	1.6	0.827	0.010	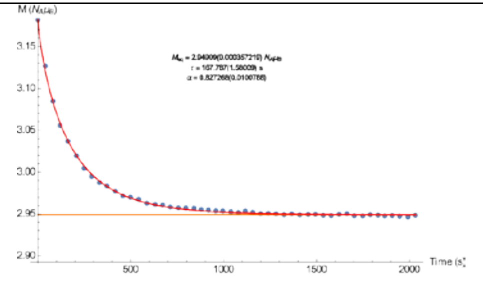

10038	3.055843	0.000251	150.1	1.4	0.815	0.009	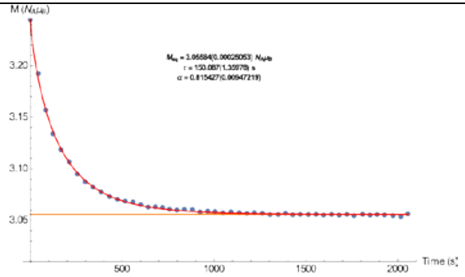
11042	3.252843	0.000226	141.5	1.8	0.863	0.015	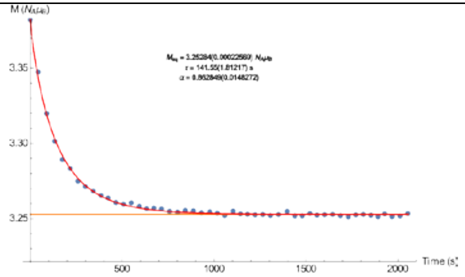
12044	3.431998	0.000246	130.3	2.7	0.767	0.020	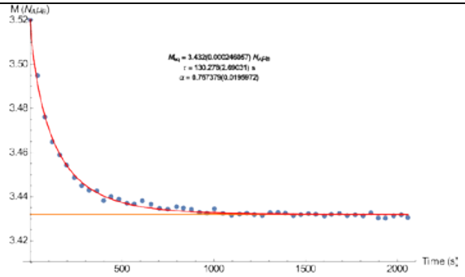

[a] M_{eq} fixed to theoretical value for m_J = ±15/2 doublet scaled to the experimental M_{sat} value at H_i = 50 kOe.

Supplementary Table 11. DC decay data for **1** measured at 7 K.

H_{ext} (Oe)	M_{eq} (μ_B mol^{-1})	ΔM_{eq} (μ_B mol^{-1})	τ (s)	$\Delta\tau$ (s)	α	$\Delta\alpha$	Figure
526	0.167546	0.000774	205.8	0.8	0.884	0.004	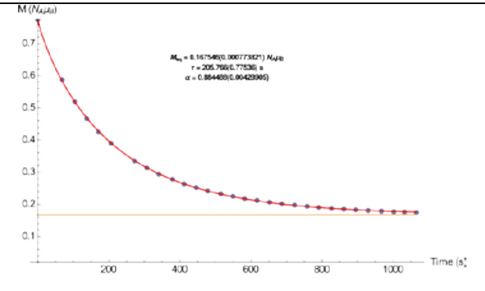
1030	0.343315	0.003024	263.7	2.2	0.941	0.008	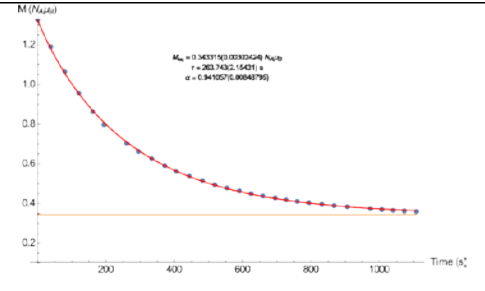
1430	0.457661	0.001215	273.6	1.2	0.910	0.005	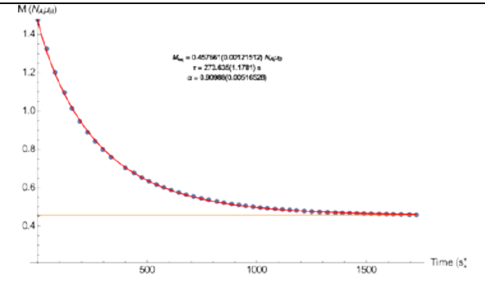
1634	0.514830	0.000435	276.0	0.8	0.885	0.003	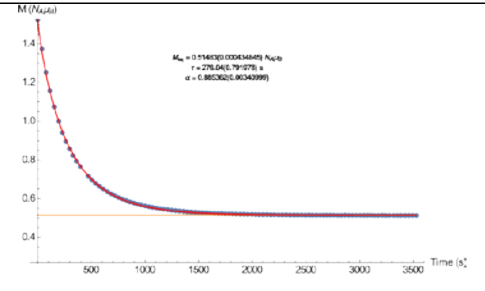
1831	0.577145	0.000344	274.6	0.6	0.882	0.003	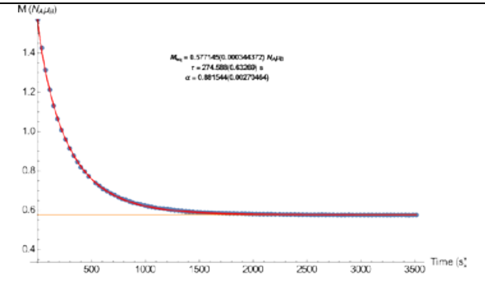
2032	0.640182	0.000384	276.5	0.7	0.892	0.003	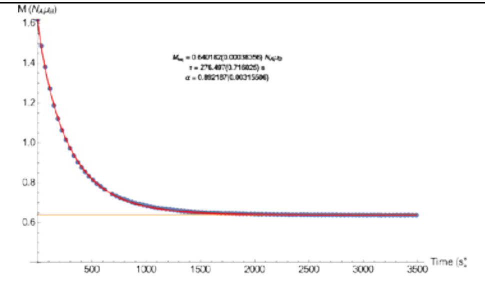

2233	0.702476	0.000382	274.1	0.7	0.891	0.003	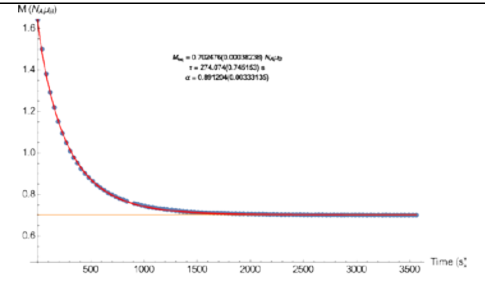
2434	0.764989	0.000357	271.9	0.7	0.890	0.003	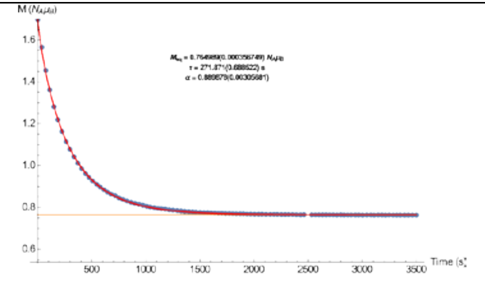
2634	0.826789	0.000328	271.3	0.7	0.892	0.003	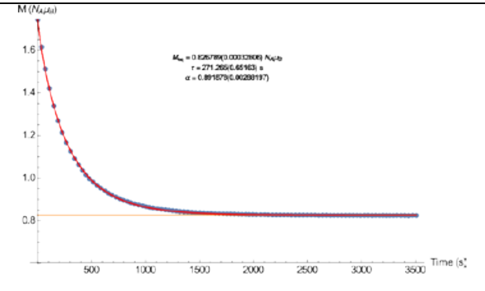
2835	0.888103	0.000375	268.1	0.8	0.890	0.003	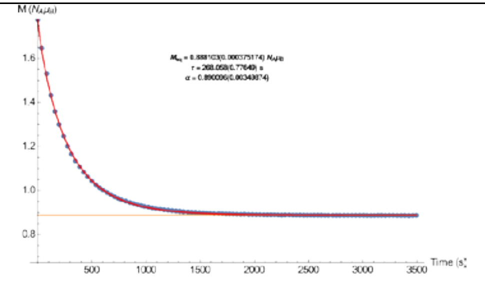
3036	0.949295	0.000248	263.6	0.5	0.877	0.002	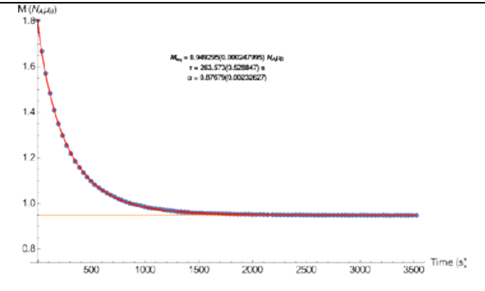
3236	1.010068	0.000313	265.8	0.7	0.892	0.003	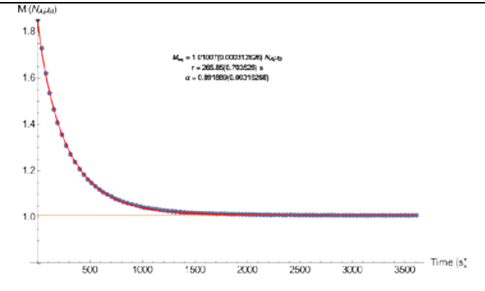

3436	1.070385	0.000337	260.4	0.8	0.886	0.003	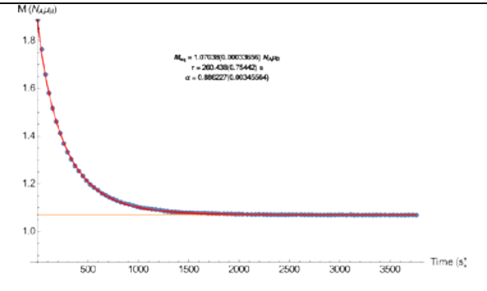
3639	1.130627	0.000236	255.9	0.6	0.878	0.003	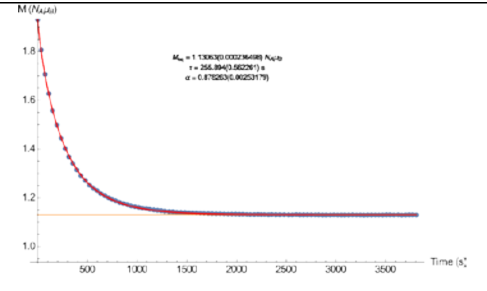
3839	1.190220	0.000278	252.4	0.7	0.880	0.003	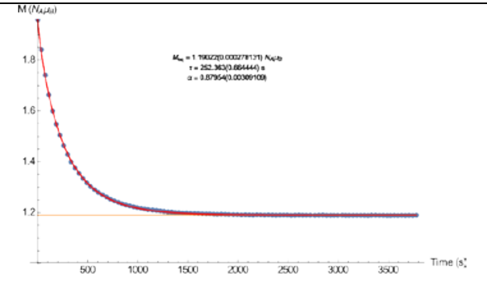
4039	1.249488	0.000295	250.8	0.7	0.886	0.003	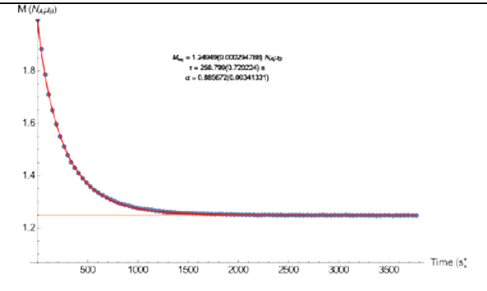
4239	1.308049	0.000299	248.4	0.8	0.891	0.004	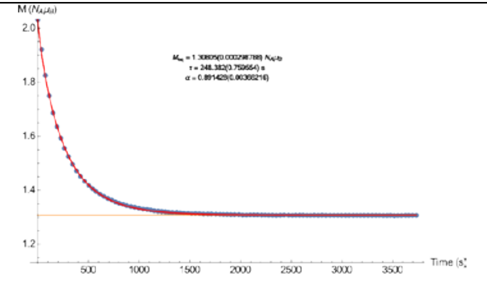
4441	1.366779	0.000308	244.2	0.8	0.895	0.004	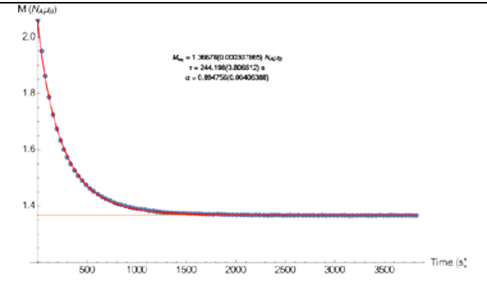

4642	1.424529	0.000256	239.5	0.7	0.890	0.003	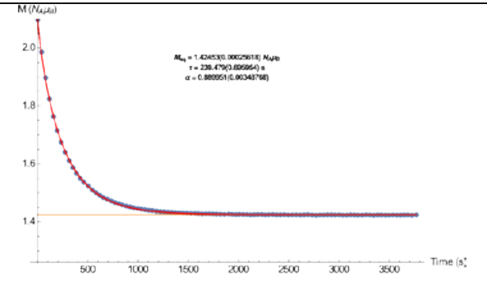
4841	1.481249	0.000249	232.5	0.7	0.884	0.004	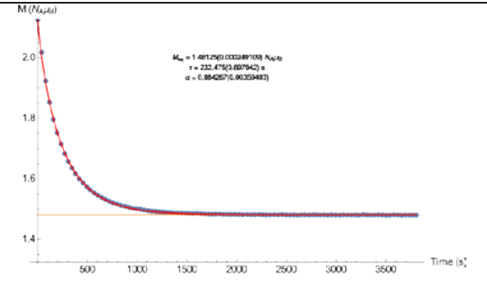
5042	1.538234	0.000239	229.4	0.7	0.888	0.004	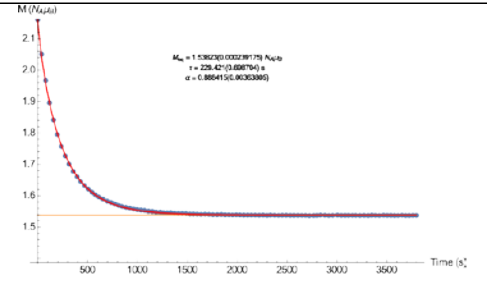
5242	1.594272	0.000194	222.5	0.6	0.878	0.003	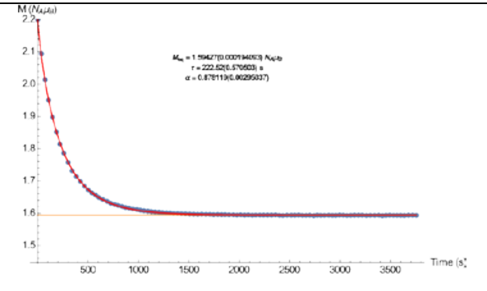
5443	1.649947	0.000254	217.2	0.8	0.882	0.004	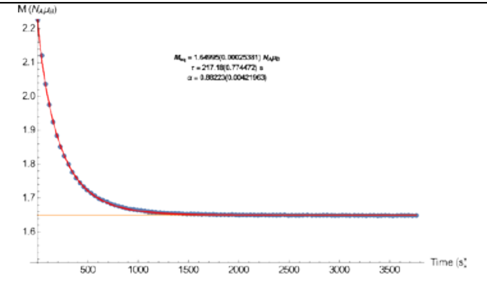
5643	1.705049	0.000212	214.1	0.7	0.888	0.004	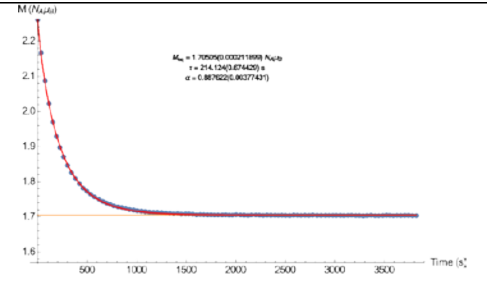

5844	1.759730	0.000209	206.1	0.7	0.878	0.004	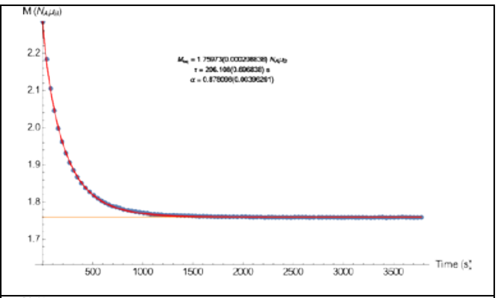
6045	1.813874	0.000215	201.8	0.7	0.883	0.004	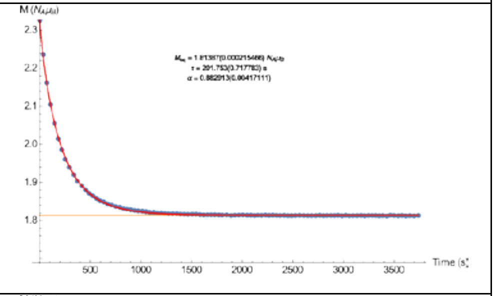
6244	1.867108	0.000218	195.3	0.8	0.880	0.005	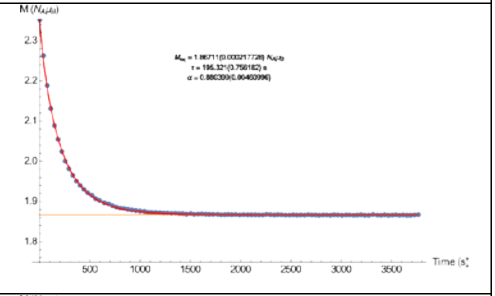
6445	1.919910	0.000207	191.0	0.8	0.880	0.005	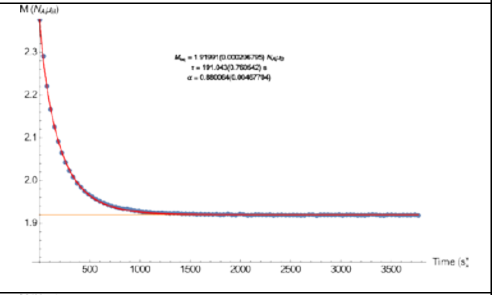
6646	1.972205	0.000171	185.3	0.7	0.877	0.004	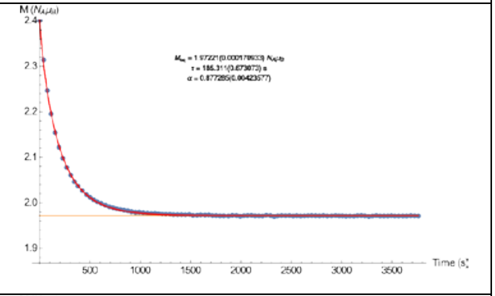
6846	2.025103	0.000251	179.2	0.7	0.876	0.005	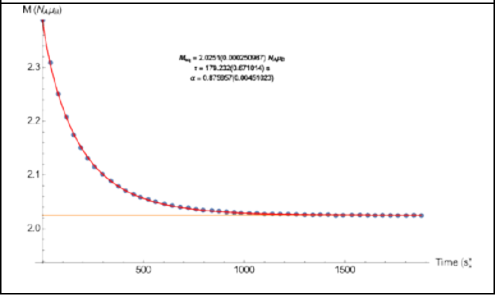

7048	2.075910	0.000292	170.4	0.7	0.870	0.005	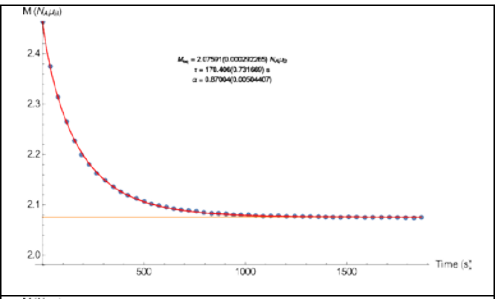
7550	2.201168	0.000317	155.2	0.7	0.867	0.006	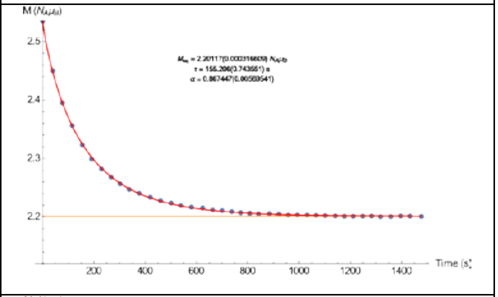
8051	2.321444	0.000383	148.1	1.1	0.895	0.009	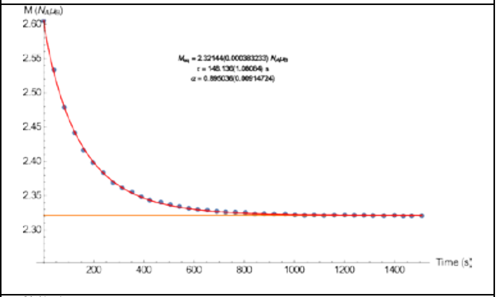
8552	2.437624	0.000248	138.1	0.8	0.882	0.007	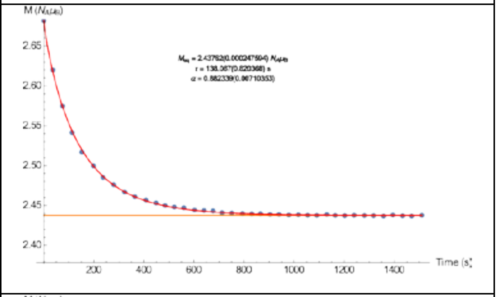
9055	2.550418	0.000257	124.8	1.0	0.864	0.010	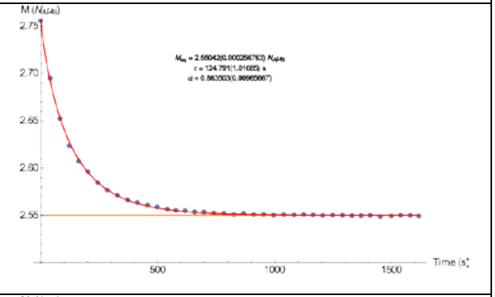
9556	2.658780	0.000259	120.6	1.2	0.872	0.012	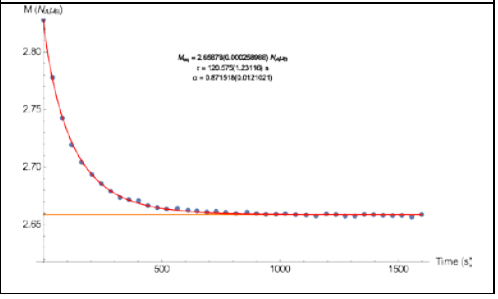

10057	2.763532	0.000192	114.0	1.1	0.890	0.012	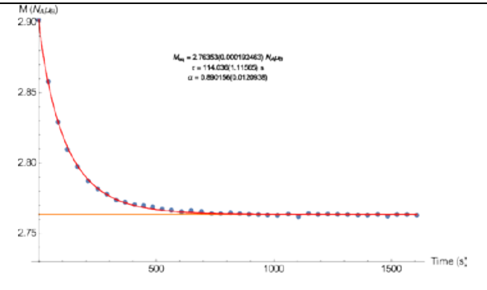

Supplementary Table 12. DC decay data for **1** measured at 8 K.

H_{ext} (Oe)	M_{eq} (μ_B mol^{-1})	ΔM_{eq} (μ_B mol^{-1})	τ (s)	$\Delta\tau$ (s)	α	$\Delta\alpha$	Figure
526	0.144038	0.000374	117.1	0.8	0.931	0.009	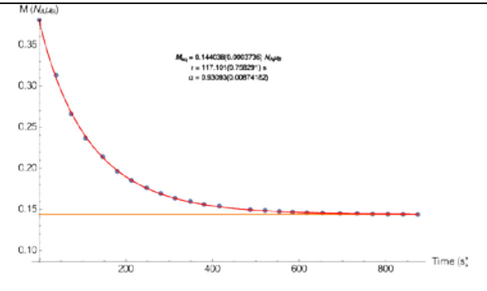
1029	0.287453	0.000623	135.0	0.5	0.916	0.006	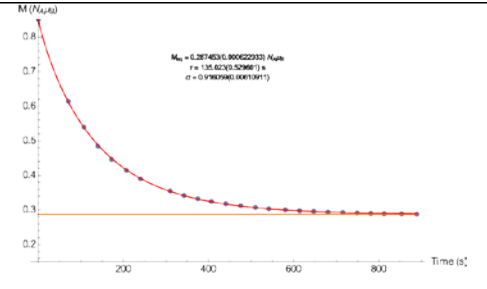
1531	0.427332	0.000709	137.8	0.6	0.919	0.006	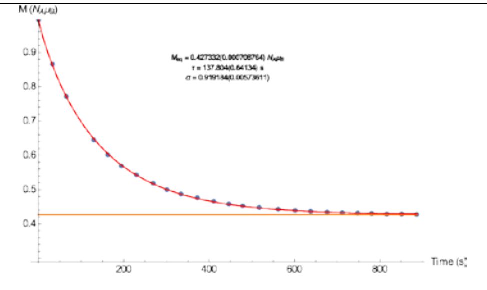
2032	0.565853	0.000401	135.2	0.4	0.902	0.003	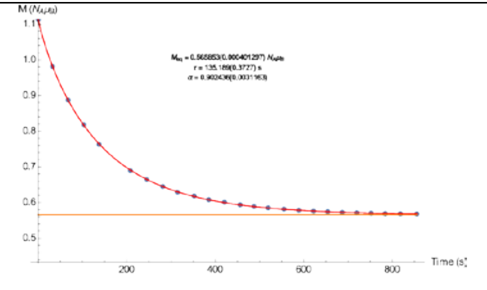
2232	0.619167	0.000354	136.0	0.6	0.902	0.005	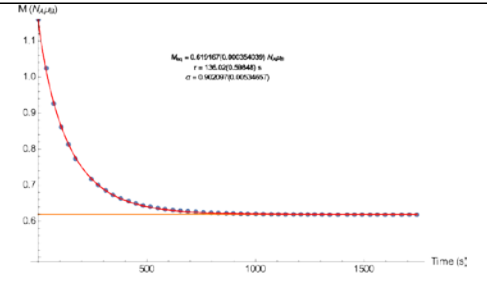
2434	0.674771	0.000301	136.7	0.5	0.907	0.005	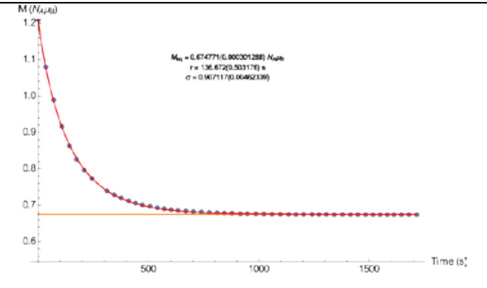

2634	0.729760	0.000192	132.8	0.3	0.889	0.003	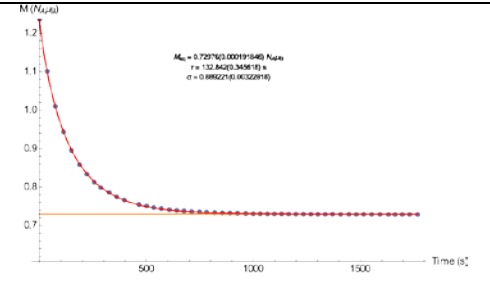
2835	0.784159	0.000357	135.4	0.6	0.913	0.006	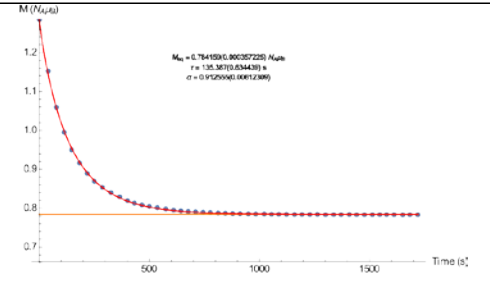
3036	0.839027	0.000259	133.5	0.5	0.902	0.004	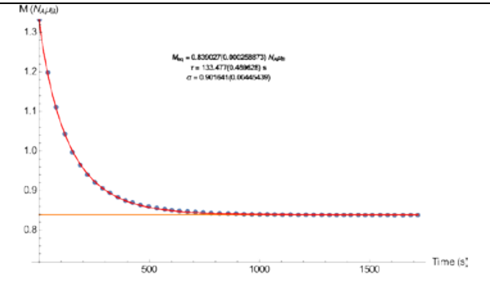
3236	0.892984	0.000302	134.1	0.6	0.917	0.006	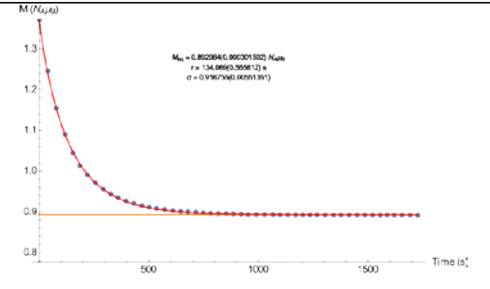
3437	0.947118	0.000270	136.1	0.5	0.923	0.005	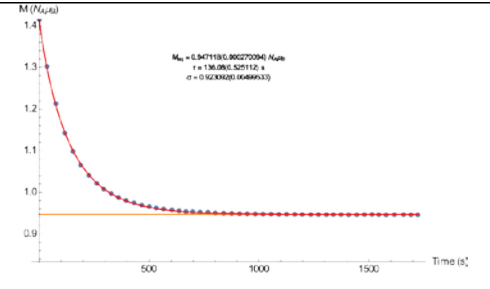
3639	1.000857	0.000264	132.5	0.5	0.912	0.005	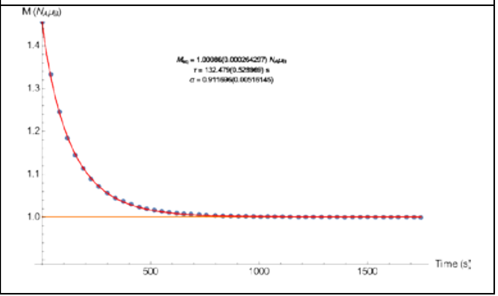

3839	1.054243	0.000274	136.2	0.6	0.933	0.006	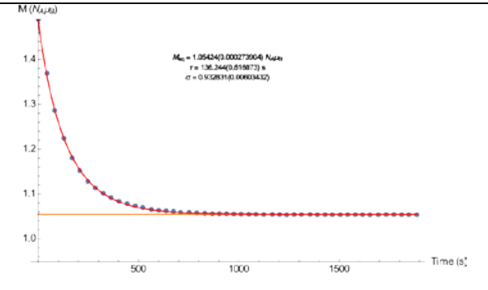
4039	1.107083	0.000281	130.3	0.6	0.907	0.006	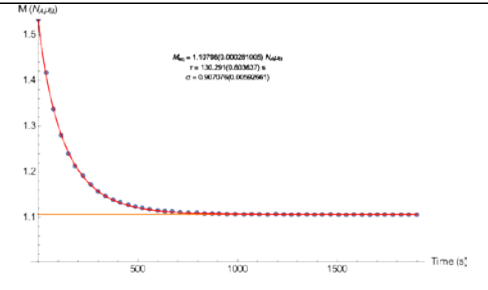
4239	1.159965	0.000191	126.9	0.4	0.893	0.004	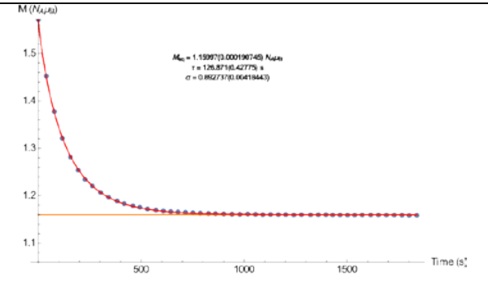
4441	1.212254	0.000259	129.7	0.6	0.911	0.006	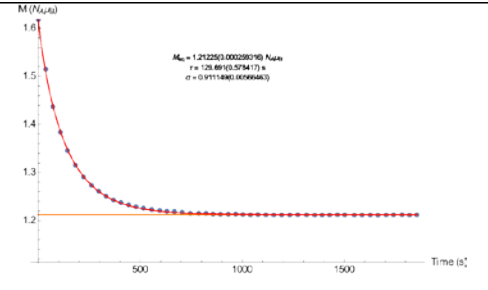
4642	1.264403	0.000232	127.3	0.6	0.909	0.006	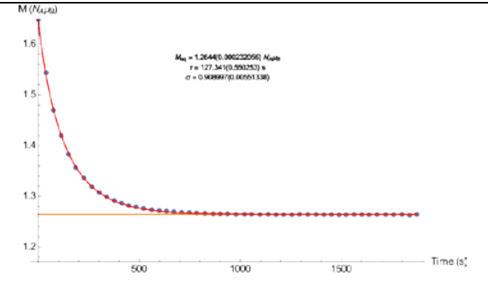
4841	1.316142	0.000127	125.0	0.3	0.894	0.003	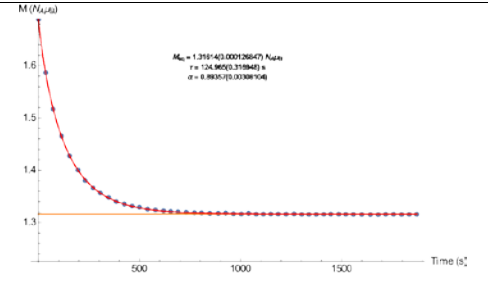

5042	1.367244	0.000151	122.4	0.4	0.888	0.004	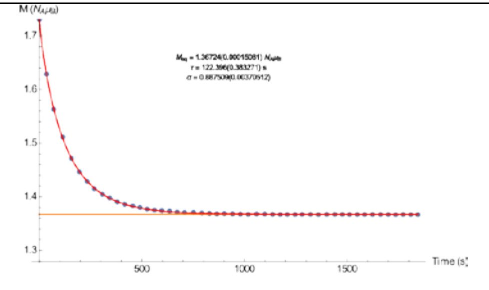
5242	1.418871	0.000232	122.6	0.6	0.909	0.006	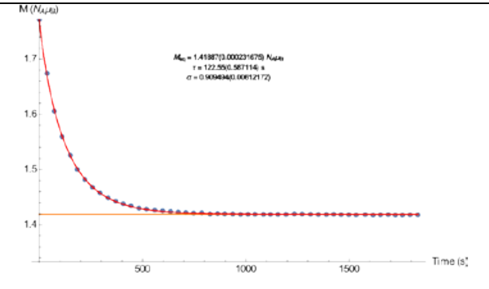
5443	1.468804	0.000210	123.3	0.6	0.922	0.006	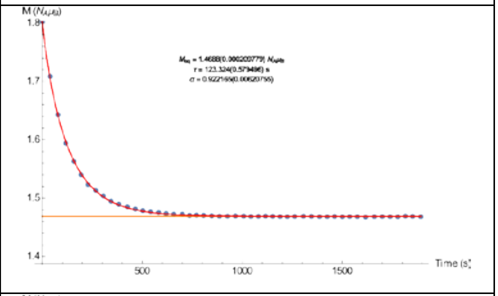
5643	1.519051	0.000236	120.0	0.6	0.916	0.007	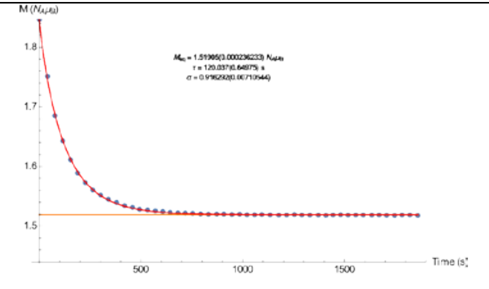
5844	1.569266	0.000214	116.1	0.6	0.894	0.007	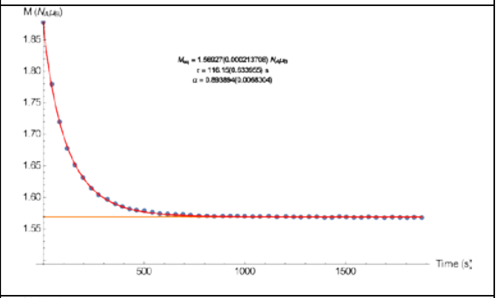
6045	1.618350	0.000170	115.0	0.5	0.891	0.006	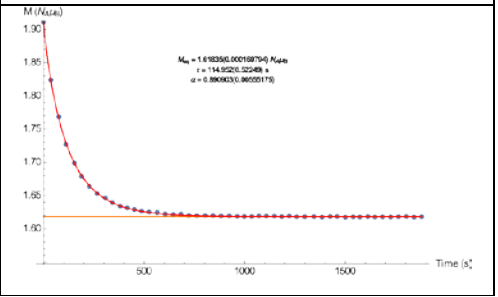

6244	1.667395	0.000188	115.4	0.6	0.910	0.007	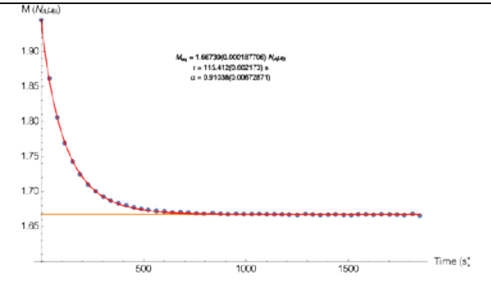
6445	1.715629	0.000170	112.6	0.6	0.919	0.007	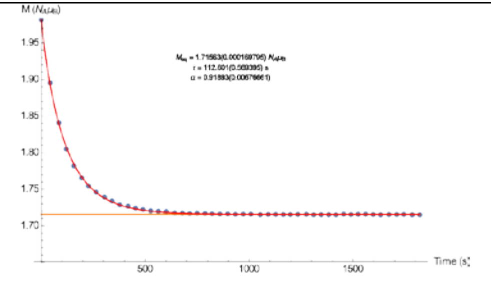
6646	1.763646	0.000217	110.7	0.7	0.904	0.008	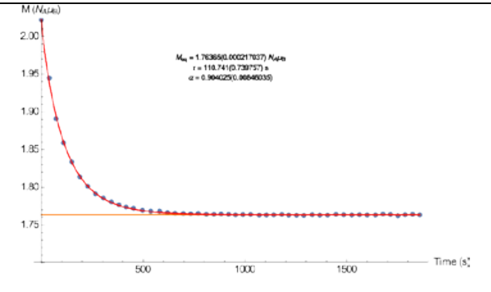
6847	1.811117	0.000154	108.0	0.6	0.880	0.006	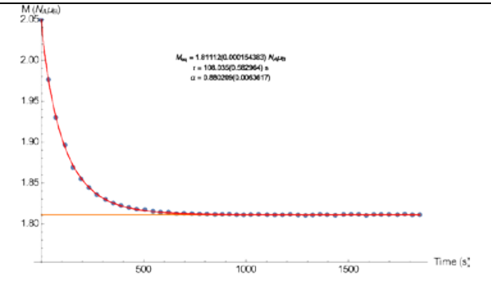
7048	1.858574	0.000195	108.4	0.8	0.915	0.009	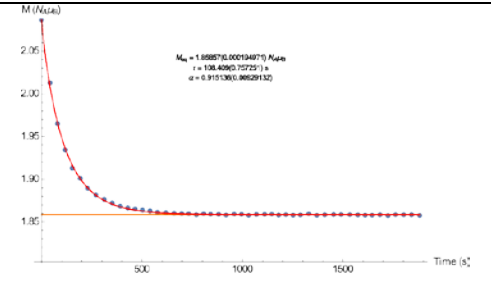
7549	1.975601	0.000321	99.2	0.8	0.905	0.011	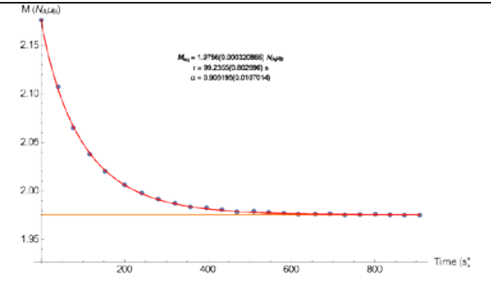

8051	2.088300	0.000279	97.0	0.8	0.923	0.011	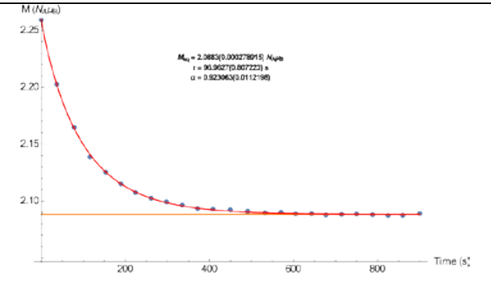
8551	2.197869	0.000207	90.7	0.7	0.886	0.010	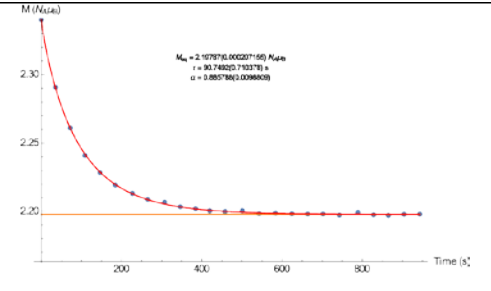
9054	2.304963	0.000242	88.2	1.0	0.888	0.014	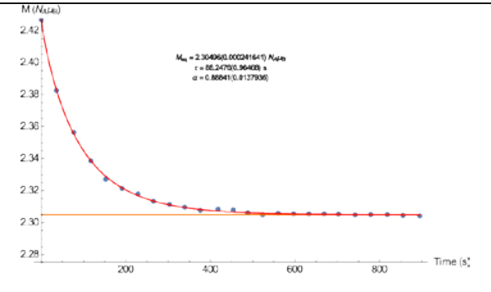
9555	2.408204	0.000231	86.9	1.2	0.877	0.017	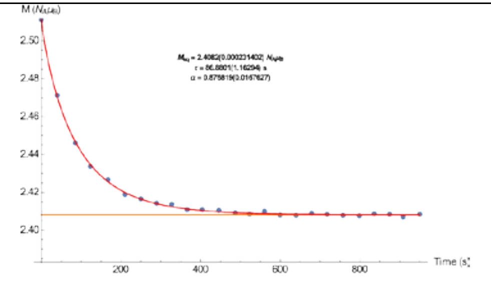

Supplementary Table 13. DC decay data for **1** measured at 9 K.

H_{ext} (Oe)	M_{eq} (μ_B mol^{-1})	ΔM_{eq} (μ_B mol^{-1})	τ (s)	$\Delta\tau$ (s)	α	$\Delta\alpha$	Figure
527	0.125627	0.000125	67.7	0.7	0.923	0.014	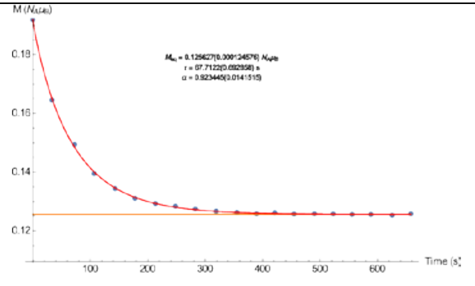
1031	0.250121	0.000159	76.1	0.5	0.893	0.008	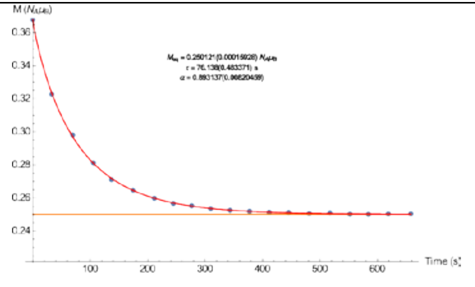
1532	0.373440	0.000109	80.3	0.3	0.944	0.006	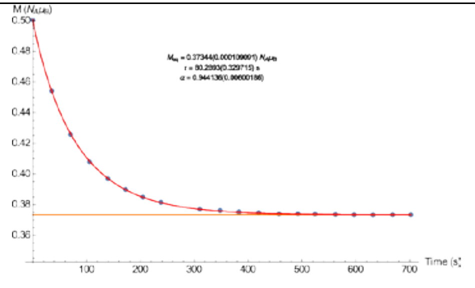
2033	0.496497	0.000131	78.7	0.4	0.936	0.007	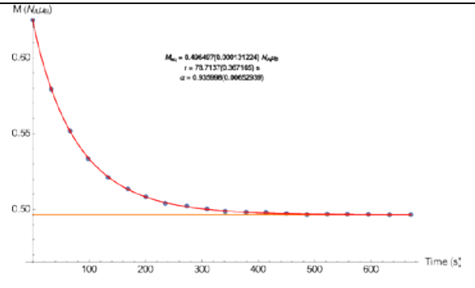
2534	0.618104	0.000109	73.5	0.3	0.918	0.006	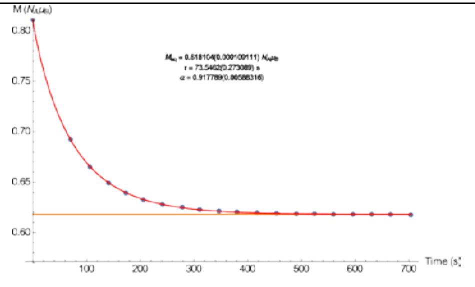
2835	0.690875	0.000120	75.6	0.4	0.918	0.007	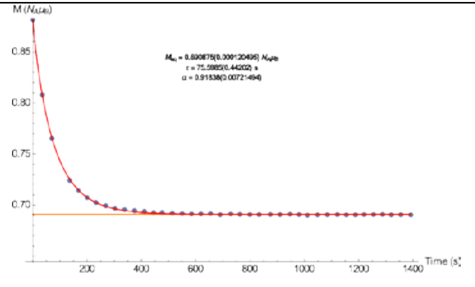

3037	0.739943	0.000075	75.4	0.3	0.960	0.006	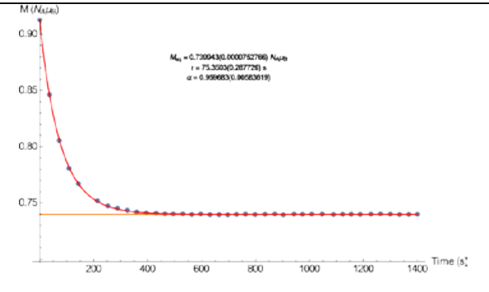
3237	0.787256	0.000079	75.0	0.3	0.927	0.005	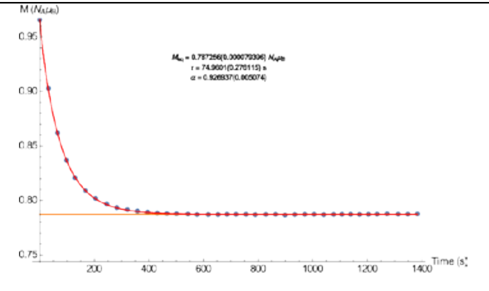
3437	0.834822	0.000073	70.3	0.3	0.867	0.005	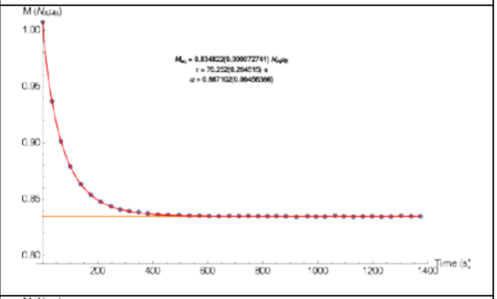
3639	0.883099	0.000091	67.6	0.3	0.833	0.006	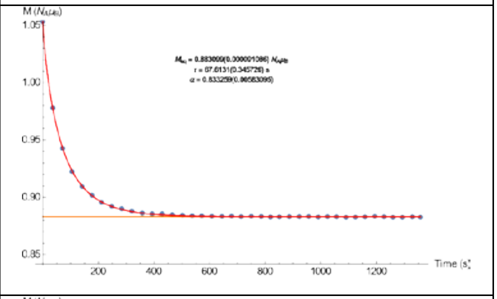
3839	0.931367	0.000106	71.4	0.4	0.908	0.008	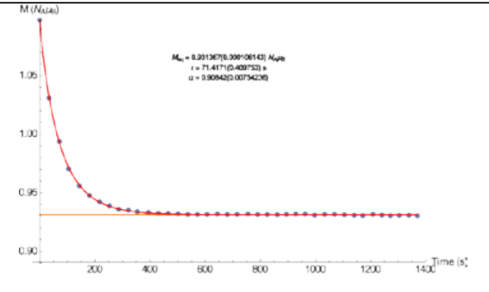
4040	0.978483	0.000083	75.3	0.3	0.982	0.007	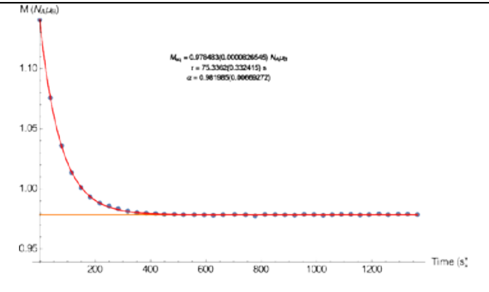

4239	1.024765	0.000101	75.2	0.4	0.946	0.008	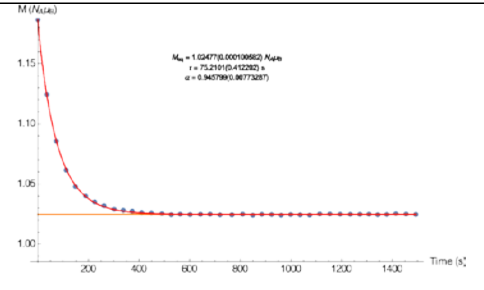
4441	1.071865	0.000110	74.3	0.5	0.928	0.009	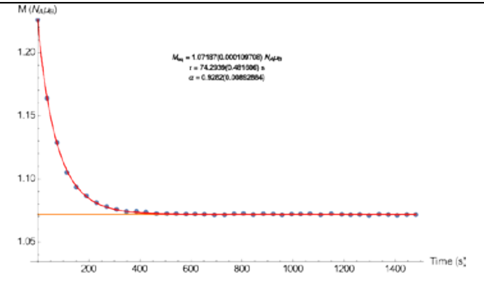
4642	1.119028	0.000152	70.5	0.7	0.877	0.012	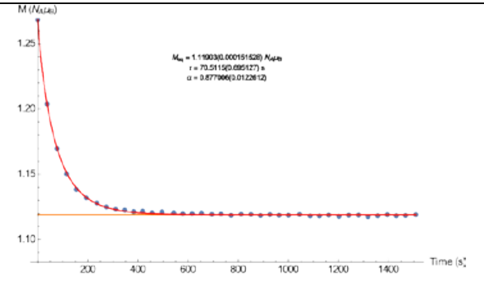
4841	1.165548	0.000103	70.6	0.5	0.960	0.010	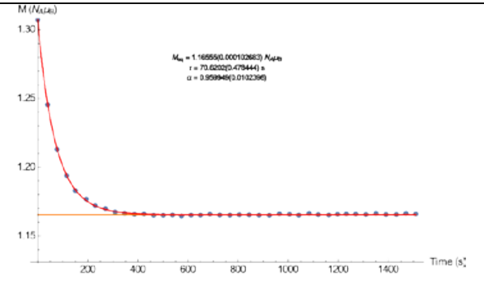
5043	1.211199	0.000118	70.2	0.6	0.922	0.011	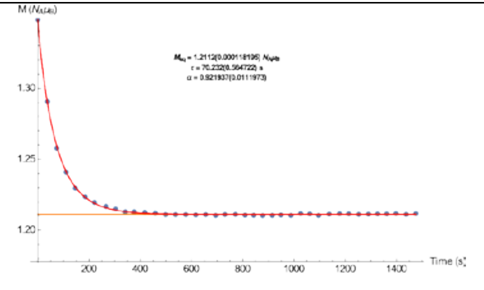
5243	1.256746	0.000134	73.3	0.7	0.937	0.013	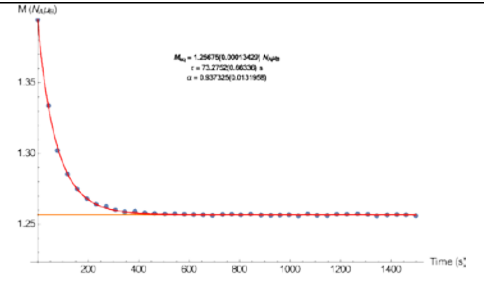

5443	1.303422	0.000153	71.5	0.7	0.941	0.015	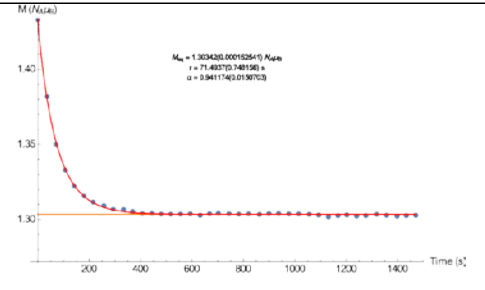
5644	1.348425	0.000099	68.6	0.5	0.910	0.010	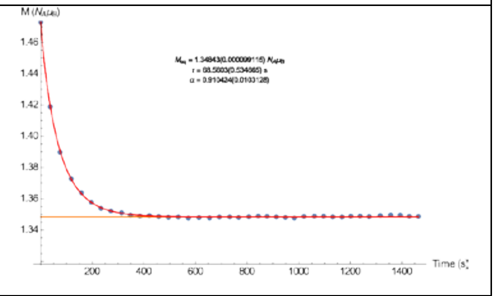
5843	1.393720	0.000128	66.3	0.7	0.890	0.014	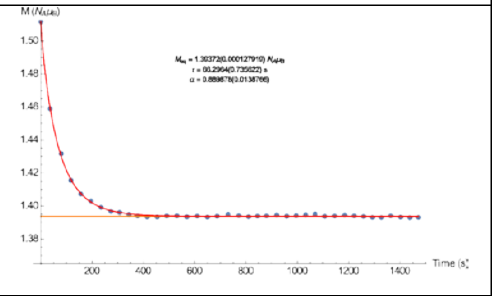
6045	1.438999	0.000120	67.8	0.7	0.940	0.014	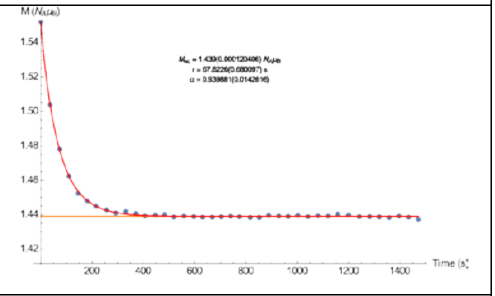
6245	1.482726	0.000146	74.4	0.9	1.016	0.020	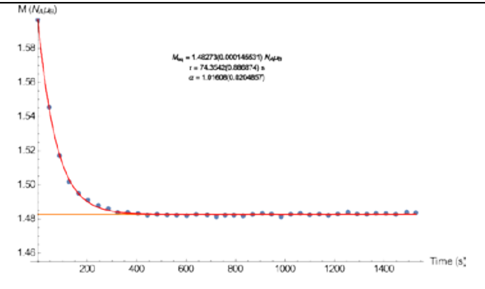
6445	1.526427	0.000141	68.9	0.9	0.930	0.018	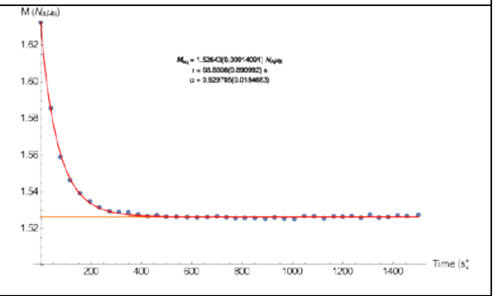

6646	1.570039	0.000116	66.4	0.8	0.874	0.014	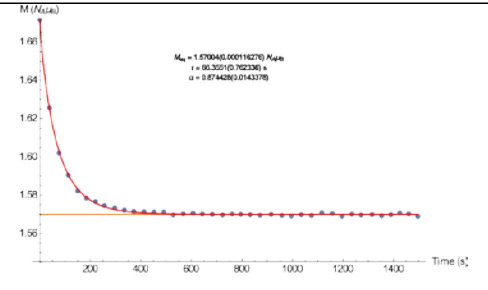
6847	1.613494	0.000105	69.7	0.7	0.892	0.013	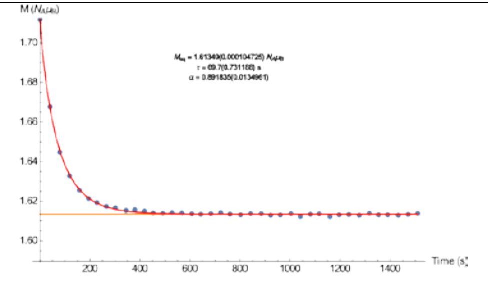
7048	1.657450	0.000189	68.6	1.5	0.837	0.025	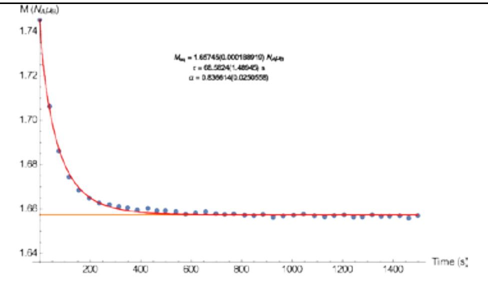
7550	1.765398	0.000195	63.4	1.0	0.891	0.020	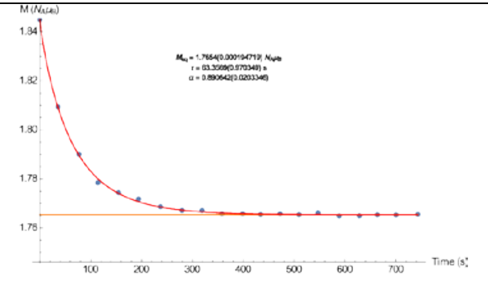
8051	1.868066	0.000240	66.6	1.4	0.910	0.029	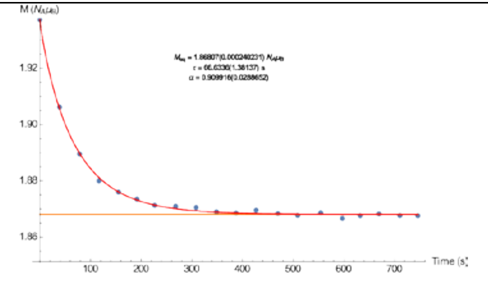
8551	1.971657	0.000264	66.8	1.7	0.981	0.041	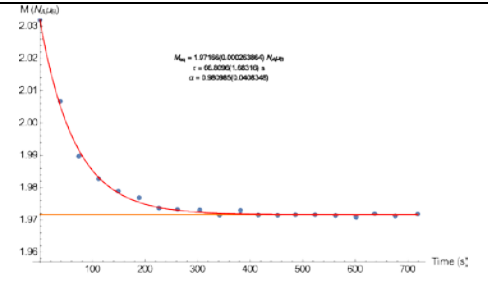

9054	2.070306	0.000163	66.4	1.2	0.889	0.023	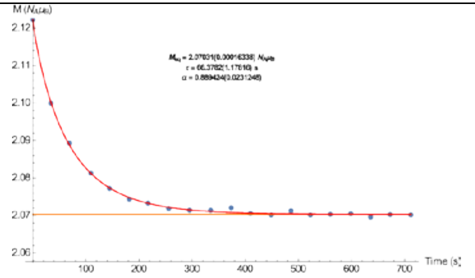
9555	2.168355	0.000135	67.1	1.3	0.981	0.030	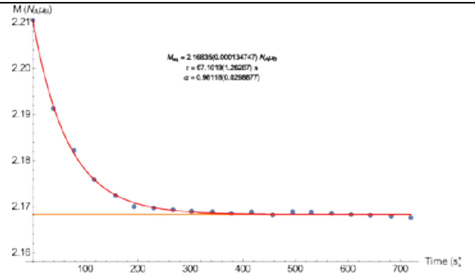

Supplementary Table 14. DC decay data for **1a** measured at 2 K.

H_{ext} (Oe)	M_{eq} (emu)	ΔM_{eq} (emu)	τ (s)	$\Delta\tau$ (s)	α	$\Delta\alpha$	Figure
21	0.001331 [a]		3246	107	0.449	0.010	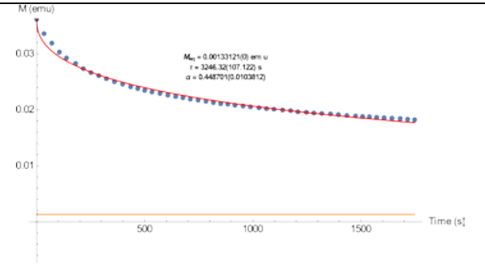
42	0.002600 [a]		2804	82	0.466	0.011	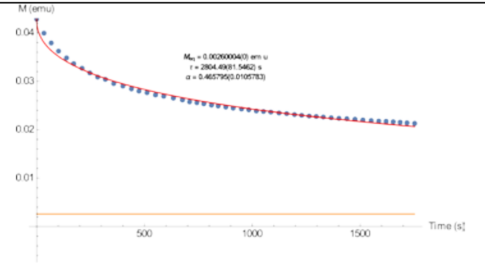
62	0.003878 [a]		4023	146	0.463	0.011	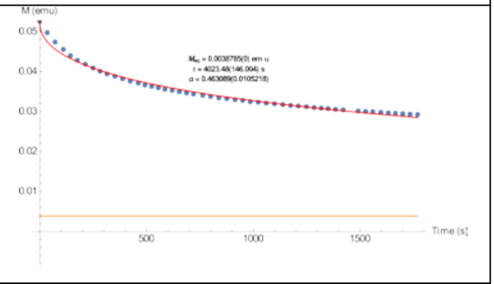
81	0.005066 [a]		7553	354	0.447	0.010	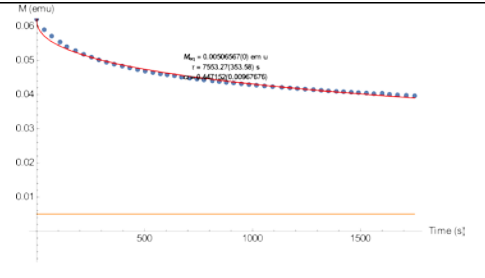
102	0.006331 [a]		13969	862	0.445	0.010	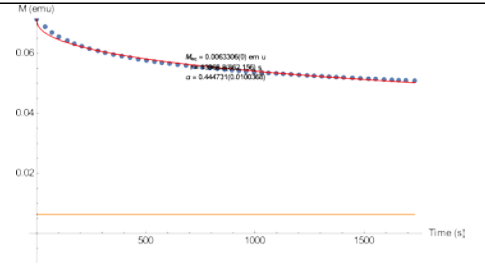
122	0.007602 [a]		43993	2395	0.369	0.006	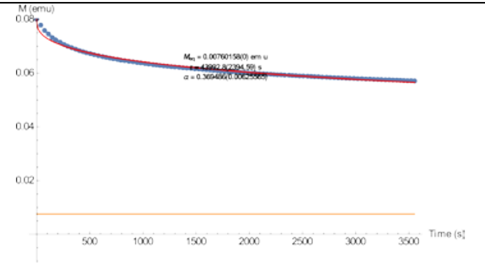

142	0.008879 [a]		68881	4122	0.367	0.006	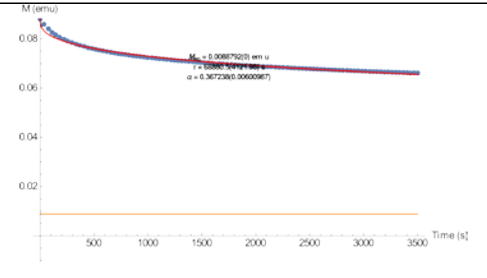
163	0.010150 [a]		97087	5825	0.365	0.005	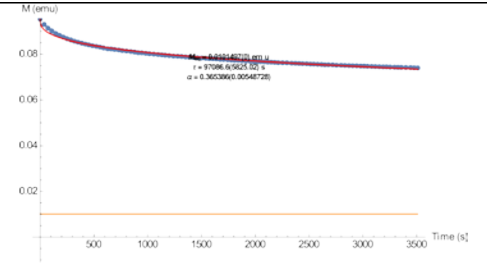
183	0.011410 [a]		120848	8026	0.367	0.006	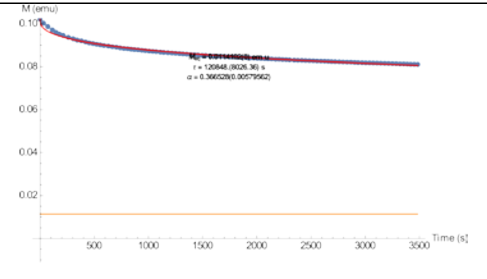
203	0.012634 [a]		133468	9899	0.353	0.006	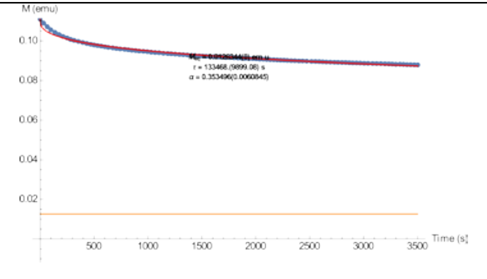
253	0.015686 [a]		470334	10565	0.305	0.002	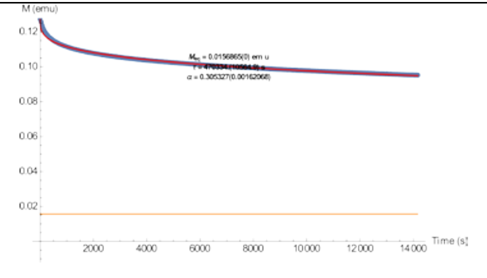
303	0.018828 [a]		564765	11402	0.324	0.001	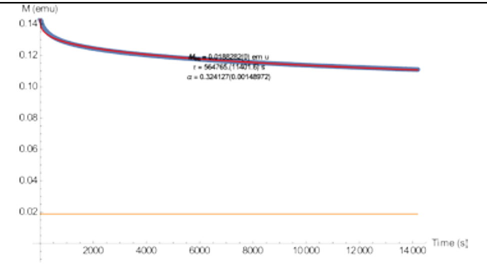
354	0.021976 [a]		626471	11834	0.341	0.001	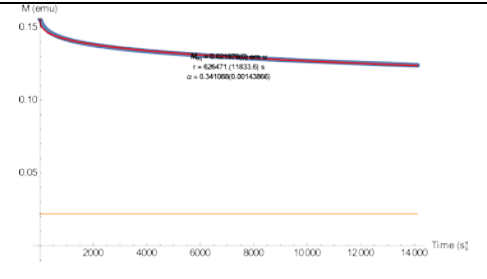

404	0.025066 [a]		647411	11577	0.353	0.001	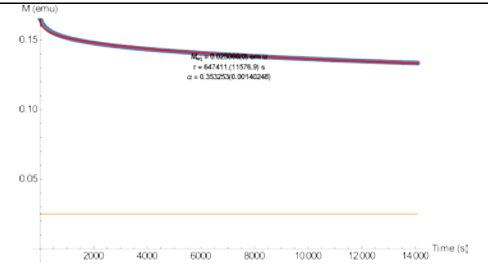
455	0.028182 [a]		669891	11064	0.357	0.001	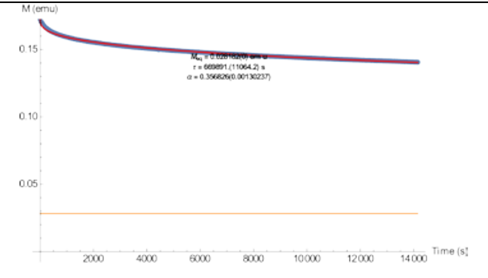
505	0.031209 [a]		628722	11079	0.370	0.001	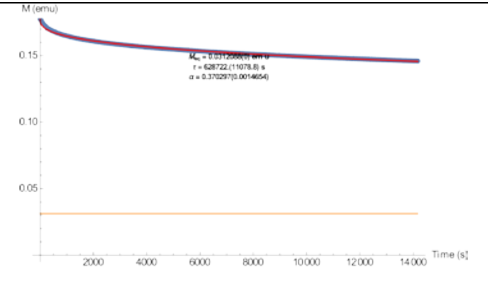
1008	0.061292 [a]		520418	4947	0.379	0.001	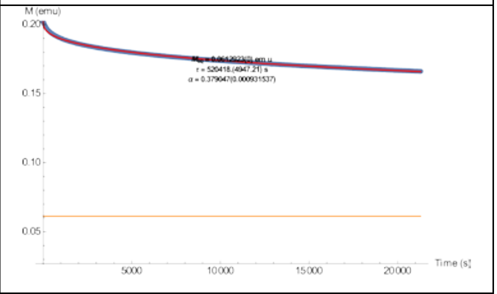
2015	0.115141 [a]		125243	447	0.459	0.001	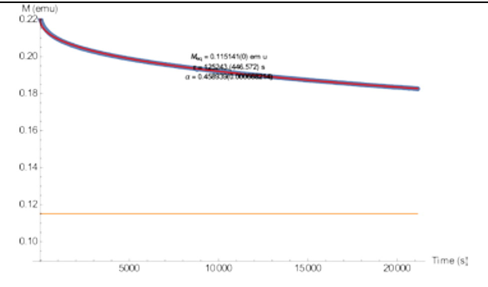
3021	0.157896 [a]		28591	42	0.588	0.001	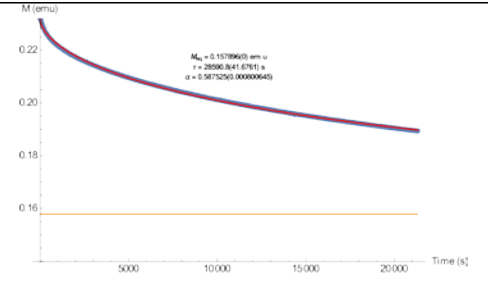

4028	0.189531 [a]		10277	7	0.719	0.001	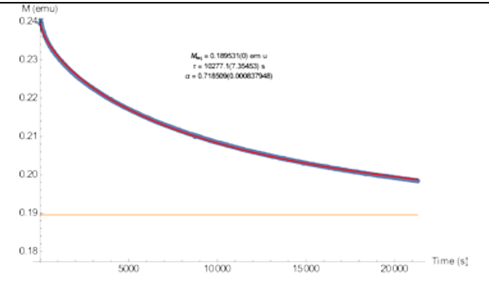
5033	0.208098	0.000055	6093	25	0.693	0.001	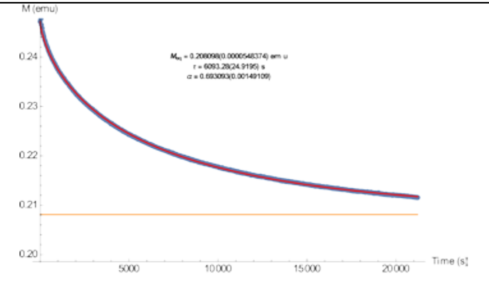
5534	0.220663 [a]		3284	8	0.790	0.003	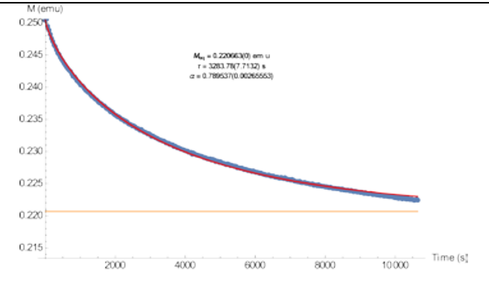
6038	0.225402	0.000089	3215	29	0.738	0.004	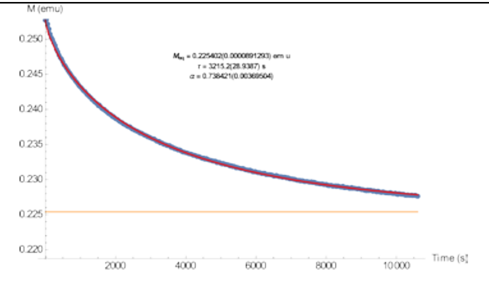
6541	0.231030	0.000072	2718	23	0.715	0.004	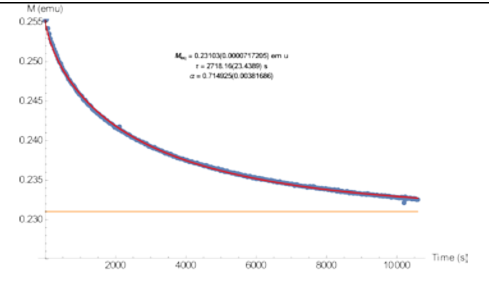
7045	0.236120	0.000059	2368	20	0.711	0.004	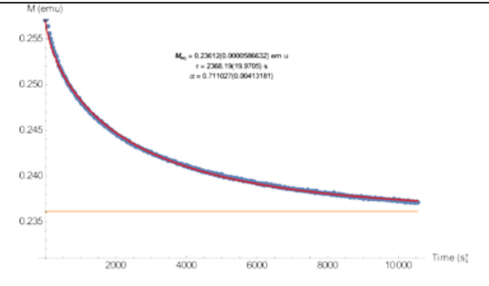

7547	0.240567	0.000054	2074	19	0.705	0.005	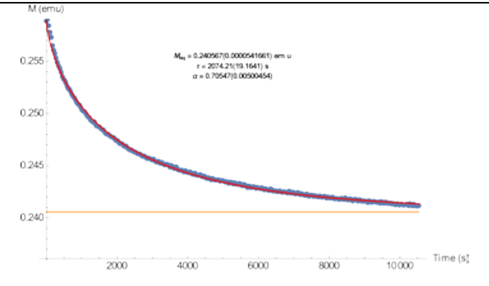
8050	0.244406	0.000042	1833	15	0.696	0.005	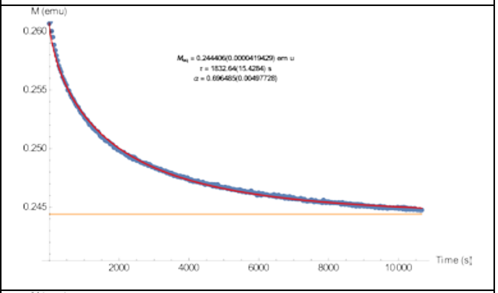
9055	0.252522	0.000137	1080	35	0.892	0.021	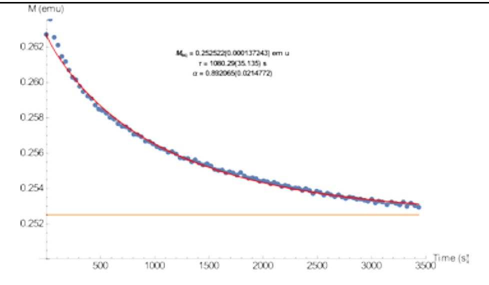
10060	0.256824	0.000099	963	29	0.856	0.021	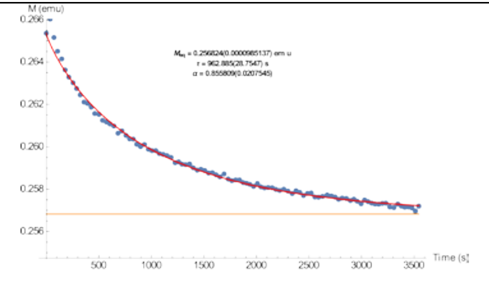
11065	0.260444	0.000070	816	22	0.886	0.024	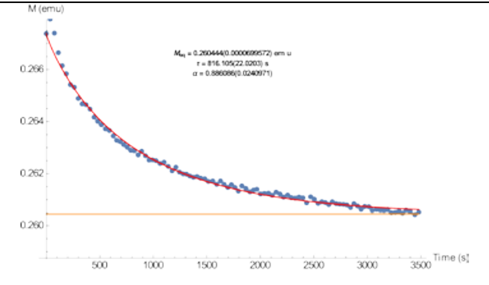
12071	0.263129	0.000059	736	21	0.790	0.020	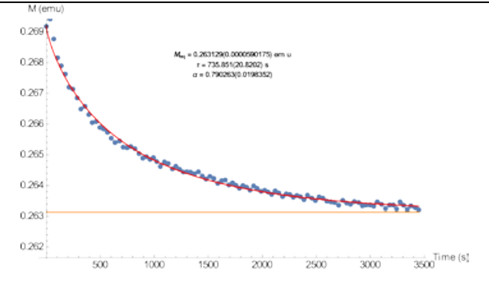

13077	0.265454	0.000041	726	18	0.873	0.023	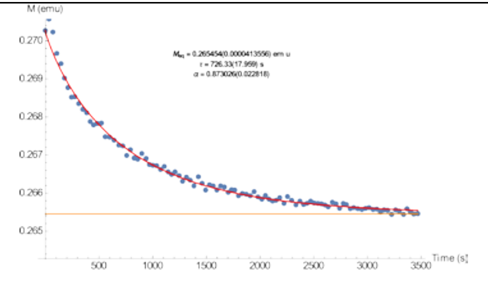
14080	0.267402	0.000035	658	19	0.988	0.039	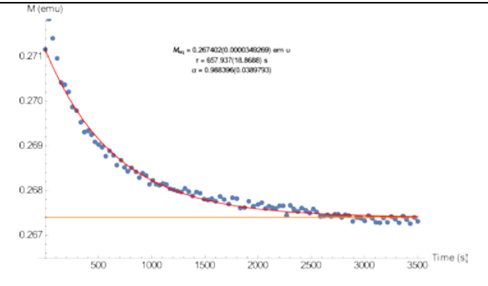
15086	0.268801	0.000033	473	14	0.675	0.019	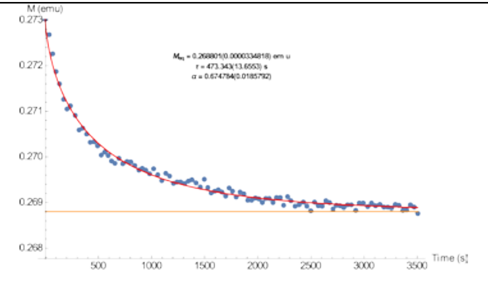

[a] M_{eq} fixed to theoretical value for $m_J = \pm 15/2$ doublet scaled to the experimental M_{sat} value at H_i = 50 kOe.

Supplementary Table 15. DC decay data for **1a** measured at 3 K.

H_{ext} (Oe)	M_{eq} (emu)	ΔM_{eq} (emu)	τ (s)	$\Delta\tau$ (s)	α	$\Delta\alpha$	Figure
21	0.000400 [a]		5643	105	0.406	0.006	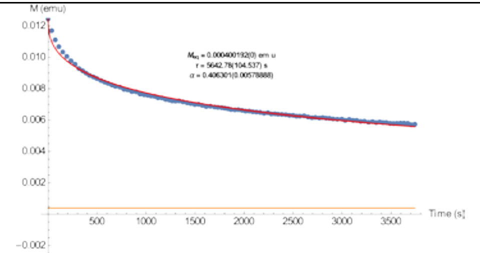
40	0.000788 [a]		4437	72	0.409	0.006	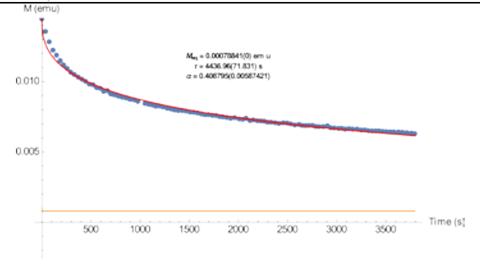
60	0.001187 [a]		5172	89	0.429	0.006	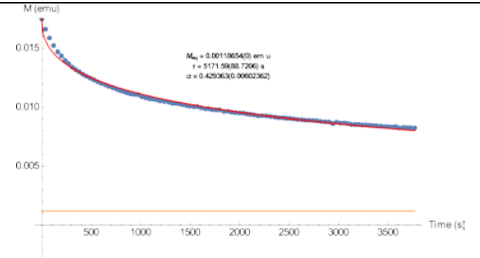
79	0.001550 [a]		7698	153	0.420	0.005	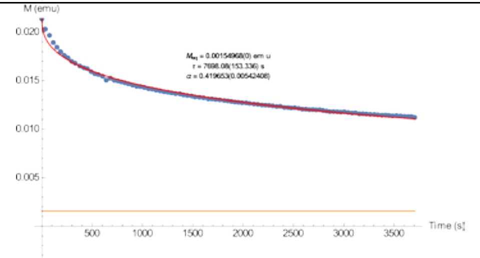
99	0.001948 [a]		13217	305	0.428	0.005	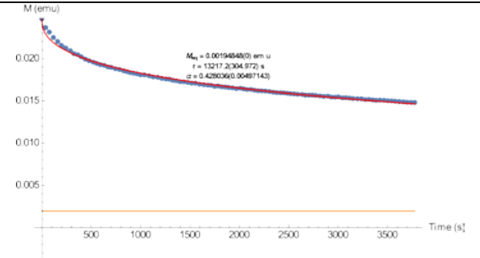
120	0.002348 [a]		21928	223	0.411	0.002	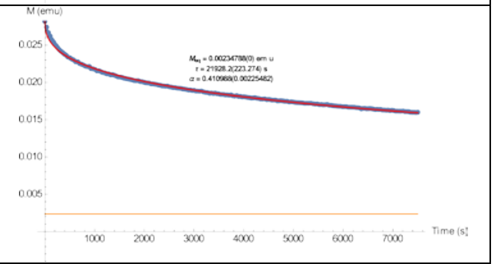
140	0.002750 [a]		28608	254	0.419	0.002	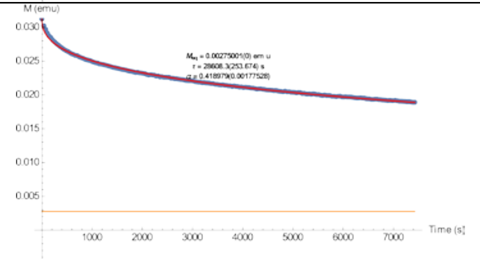

161	0.003146 [a]		33860	333	0.438	0.002	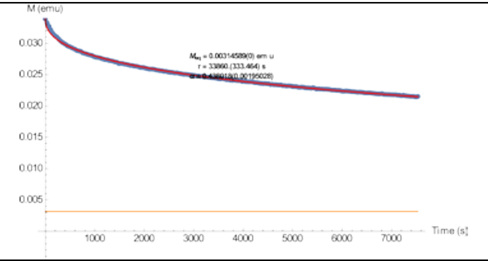
181	0.003558 [a]		36932	344	0.456	0.002	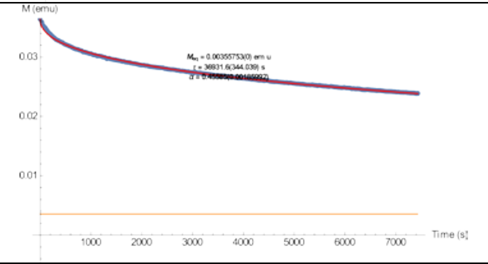
202	0.003954 [a]		39039	315	0.459	0.002	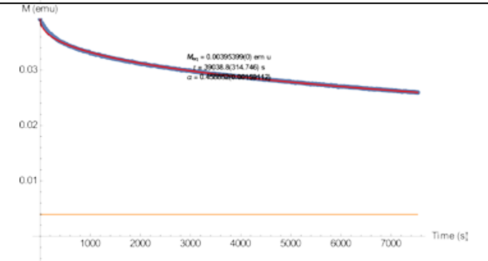
251	0.004941 [a]		37012	101	0.503	0.001	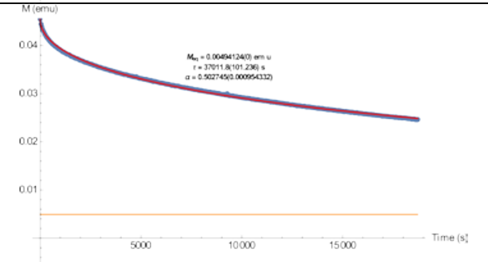
302	0.005922 [a]		38268	90	0.533	0.001	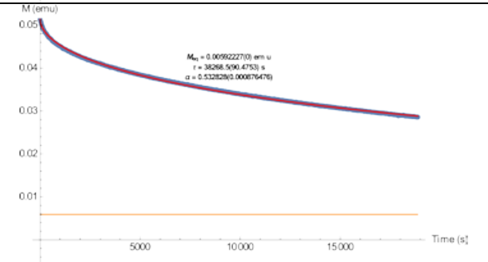
352	0.006898 [a]		39759	94	0.536	0.001	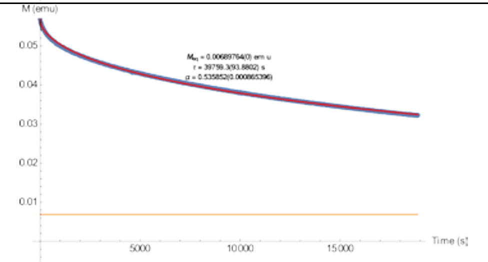
402	0.007860 [a]		40972	94	0.546	0.001	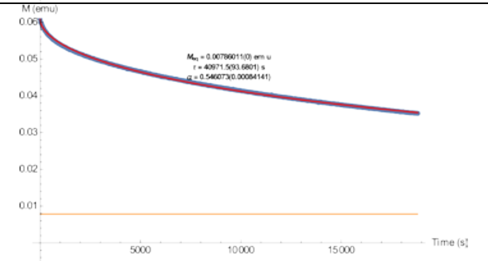

454	0.008888 [a]		40501	94	0.576	0.001	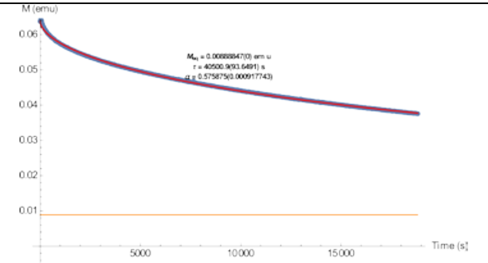
503	0.009781 [a]		39901	85	0.574	0.001	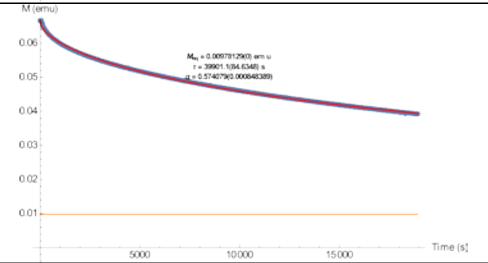
1006	0.019334 [a]		34225	46	0.575	0.001	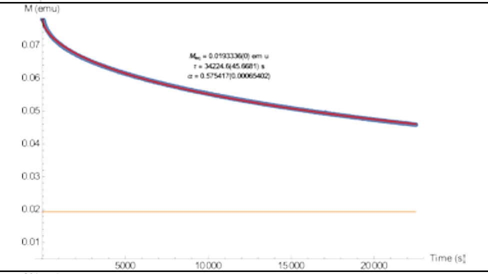
2012	0.037849 [a]		20020	20	0.597	0.001	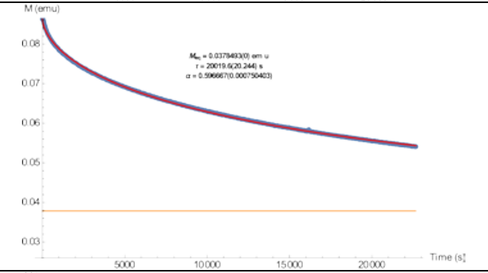
3019	0.053996 [a]		9651	12	0.687	0.001	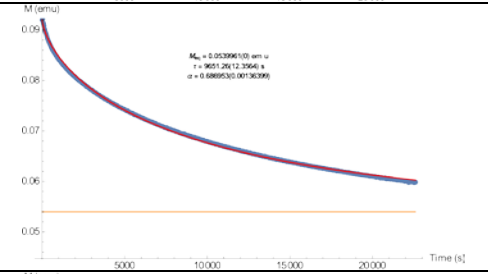
4026	0.063039	0.000030	6678	18	0.670	0.001	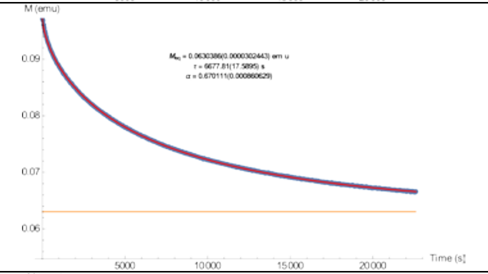
5030	0.074651	0.000028	3847	14	0.745	0.003	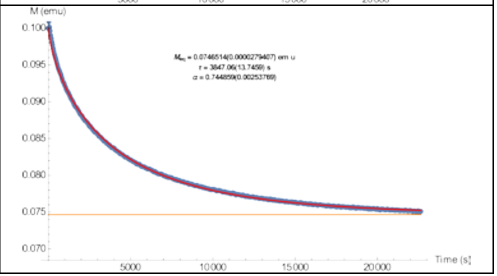

5533	0.080232	0.000059	2704	21	0.770	0.004	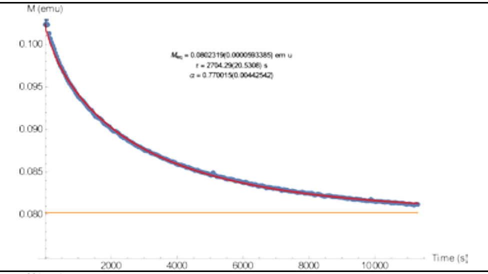
6037	0.084416	0.000042	2193	14	0.767	0.004	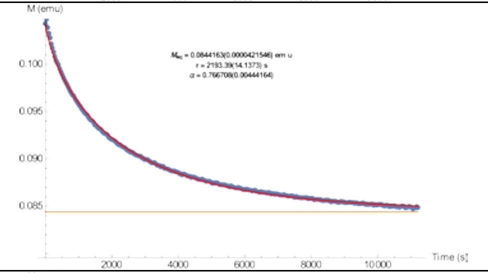
6540	0.088260	0.000038	1885	14	0.786	0.006	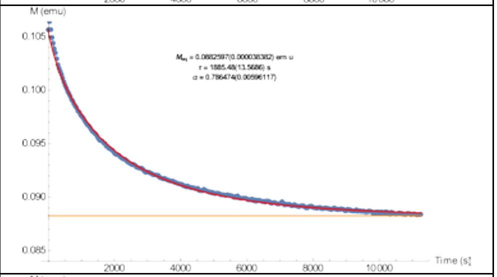
7043	0.091540	0.000035	1661	13	0.769	0.007	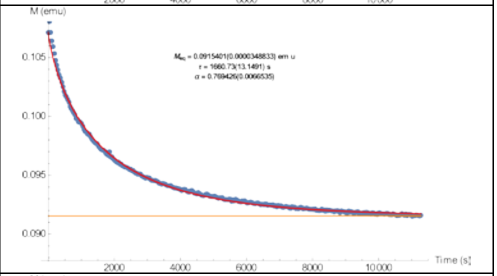
7545	0.094554	0.000024	1327	9	0.708	0.005	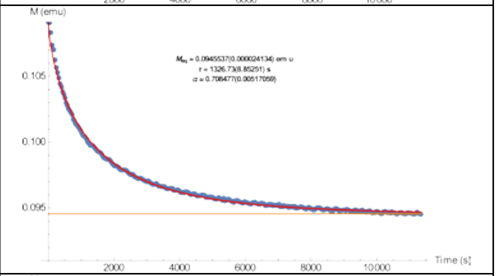
8048	0.097471	0.000021	1192	9	0.732	0.006	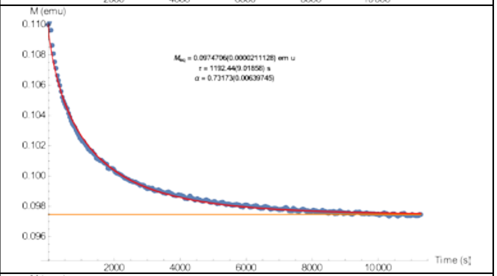
9052	0.102939	0.000045	738	11	0.722	0.009	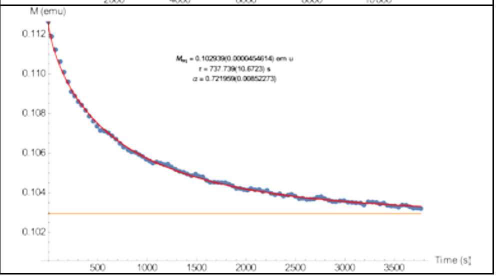

10059	0.107047	0.000035	635	10	0.735	0.010	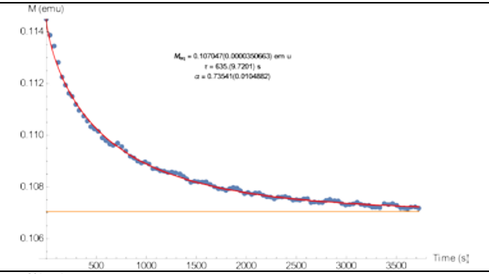
11063	0.110457	0.000036	535	11	0.695	0.014	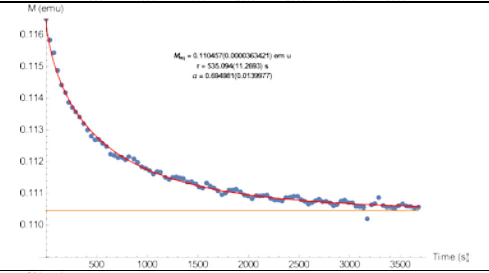
12069	0.113357	0.000031	394	9	0.628	0.015	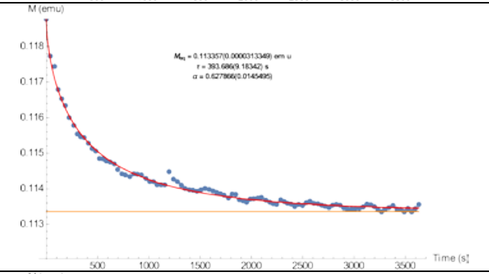
13076	0.115836	0.000024	418	10	0.697	0.019	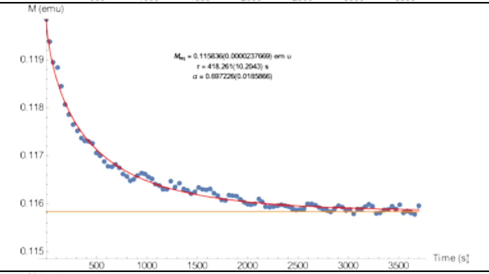
14079	0.117859	0.000021	360	10	0.639	0.017	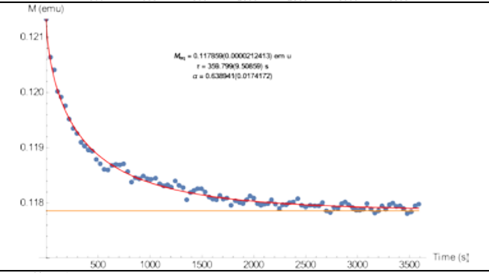
15085	0.119644	0.000020	305	11	0.649	0.025	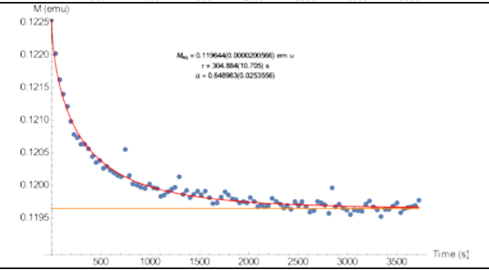

[a] M_{eq} fixed to theoretical value for $m_J = \pm 15/2$ doublet scaled to the experimental M_{sat} value at H_i = 50 kOe.

Supplementary Table 16. DC decay data for **1a** measured at 4 K.

H_{ext} (Oe)	M_{eq} (emu)	ΔM_{eq} (emu)	τ (s)	$\Delta\tau$ (s)	α	$\Delta\alpha$	Figure
18	0.000269 [a]		2122	9	0.524	0.003	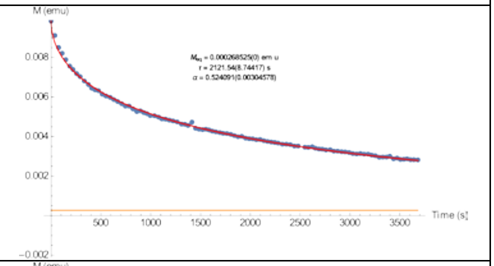
39	0.000574 [a]		1795	7	0.547	0.003	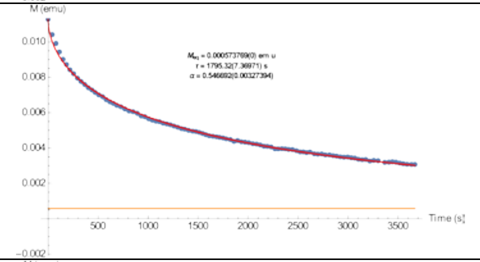
60	0.000872 [a]		1838	6	0.563	0.003	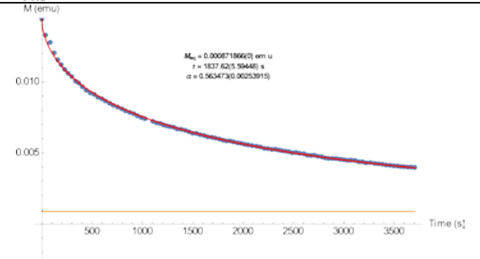
78	0.001149 [a]		2467	8	0.606	0.003	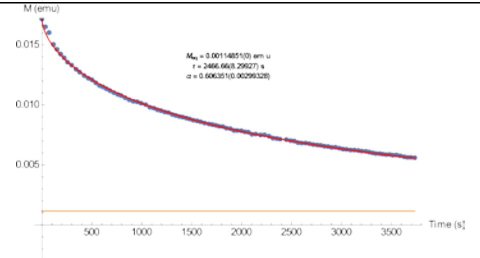
99	0.001448 [a]		3151	6	0.628	0.002	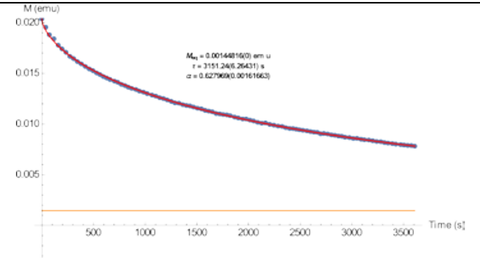
119	0.001749 [a]		3600	3	0.652	0.001	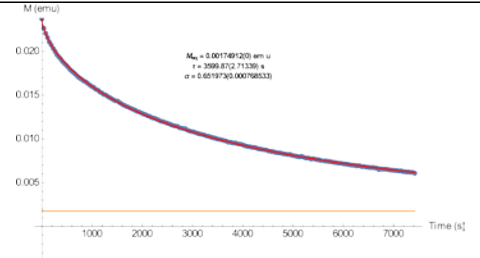
140	0.002057 [a]		3978	3	0.671	0.001	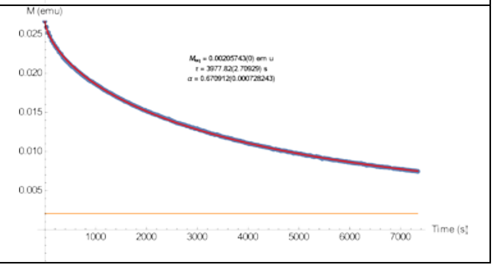

161	0.002355 [a]		4281	3	0.688	0.001	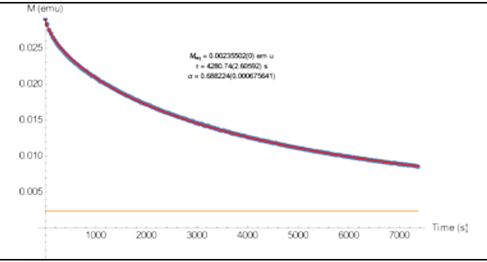
181	0.002652 [a]		4493	2	0.703	0.001	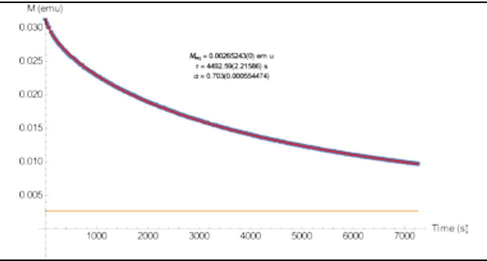
201	0.002956 [a]		4579	2	0.703	0.001	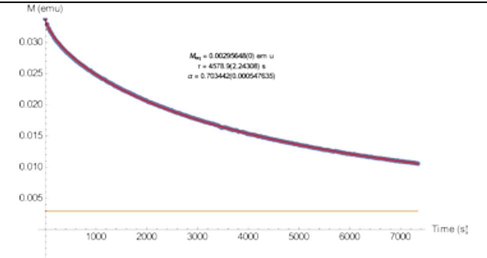
250	0.003677 [a]		4829	4	0.704	0.001	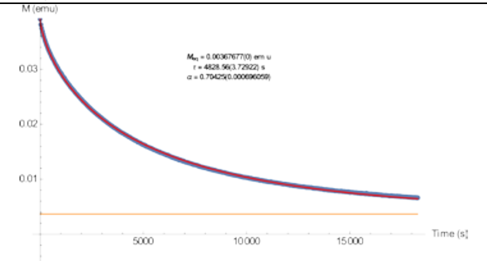
301	0.004410 [a]		5058	6	0.721	0.001	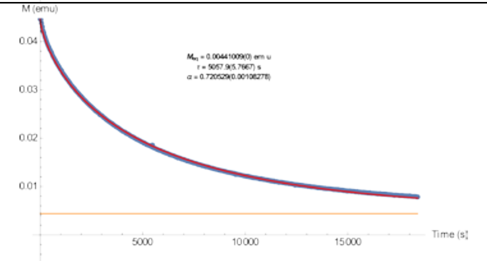
352	0.005154 [a]		5294	7	0.734	0.001	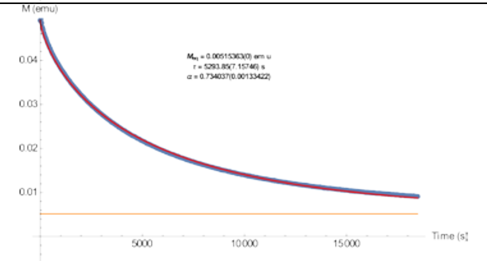
402	0.005897 [a]		5260	5	0.721	0.001	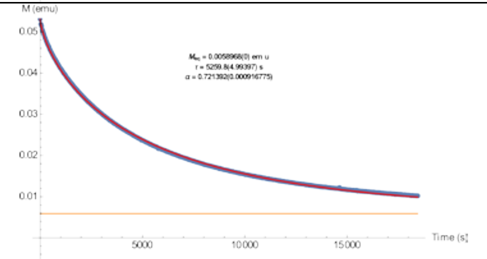

453	0.006660 [a]		5271	4	0.721	0.001	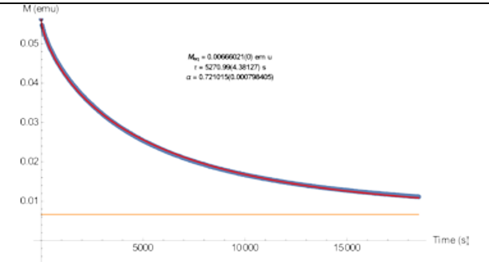
502	0.007356 [a]		5327	5	0.727	0.001	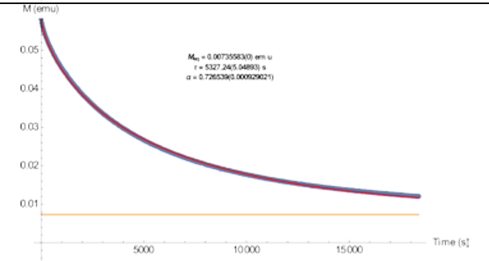
1005	0.014650 [a]		4983	3	0.729	0.001	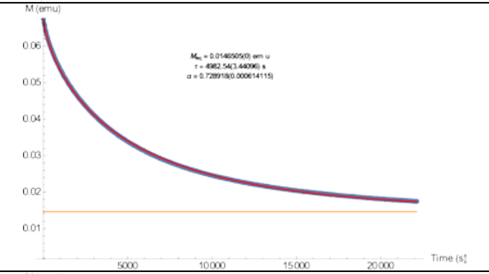
2012	0.027726	0.000016	4190	4	0.737	0.001	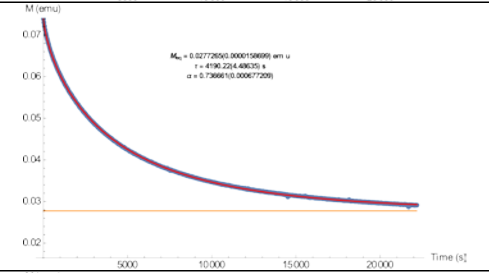
3019	0.039085	0.000013	3419	4	0.728	0.001	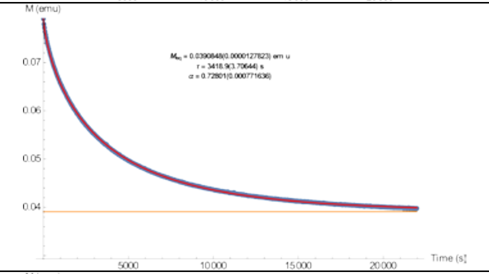
4025	0.049846	0.000015	2558	5	0.751	0.002	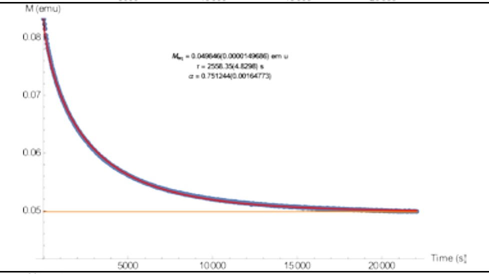
5030	0.059796	0.000012	1827	5	0.769	0.002	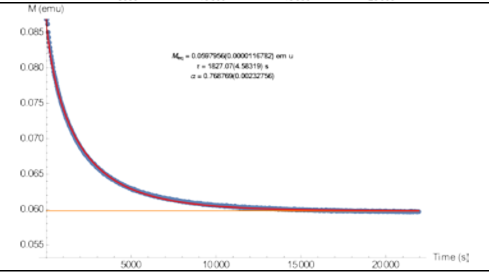

5533	0.064756	0.000029	1510	7	0.817	0.005	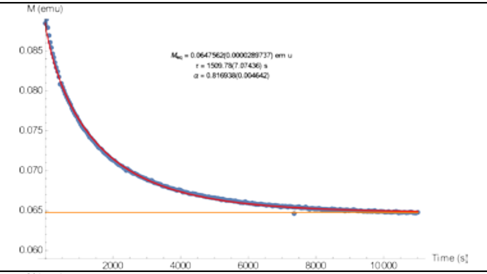
6036	0.068991	0.000023	1243	6	0.800	0.005	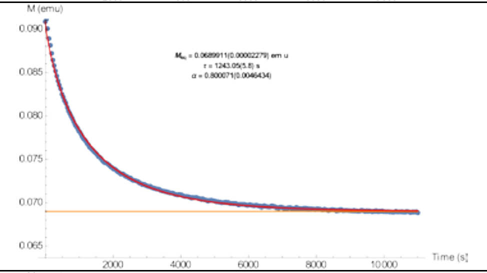
6539	0.072918	0.000021	1070	6	0.788	0.005	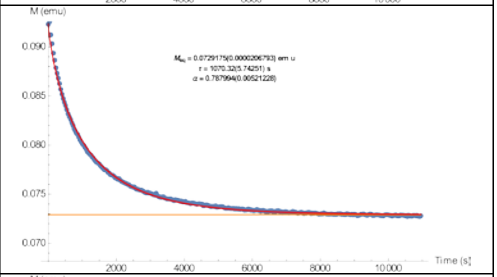
7042	0.076642	0.000019	951	6	0.805	0.007	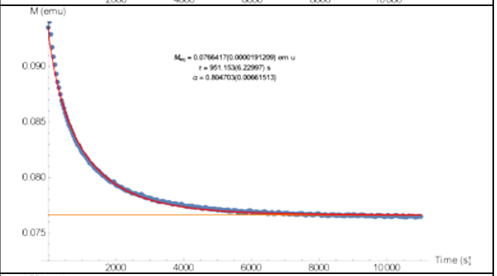
7544	0.080087	0.000016	821	6	0.792	0.007	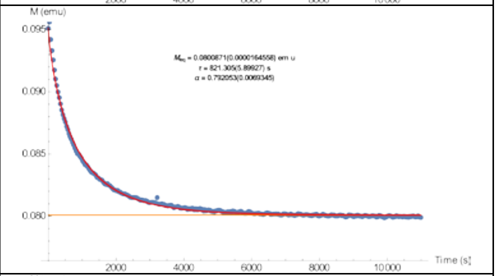
8048	0.083392	0.000014	723	6	0.817	0.008	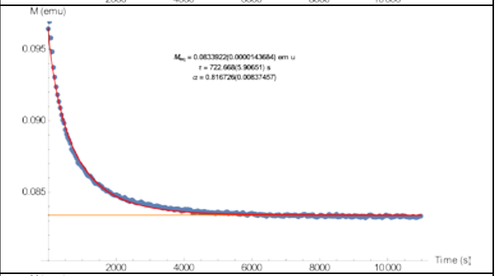
9053	0.089531	0.000021	458	4	0.751	0.007	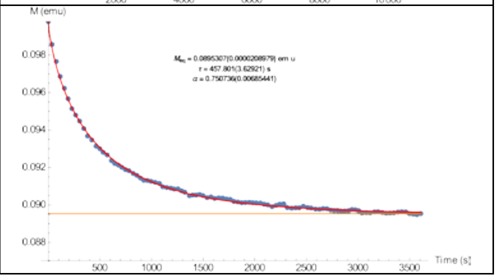

10059	0.094563	0.000018	364	4	0.690	0.008	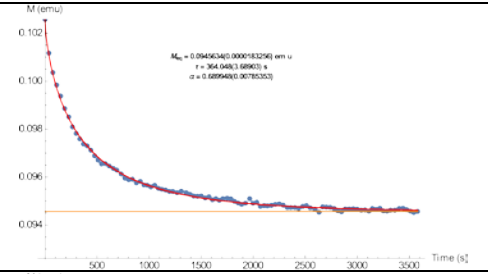
11064	0.098986	0.000012	350	3	0.739	0.009	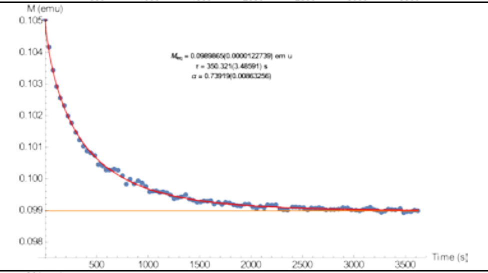
12069	0.102829	0.000012	324	5	0.763	0.013	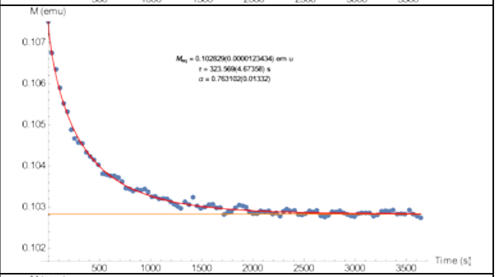
13076	0.106124	0.000013	280	5	0.675	0.014	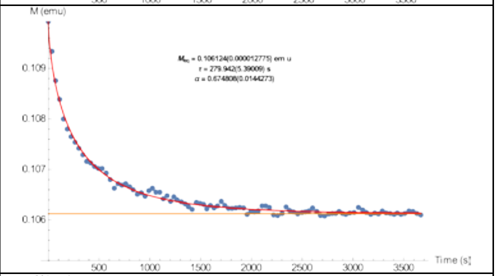
14079	0.109006	0.000014	244	7	0.630	0.019	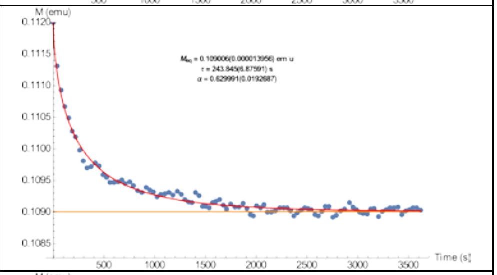
15086	0.111505	0.000014	266	10	0.698	0.028	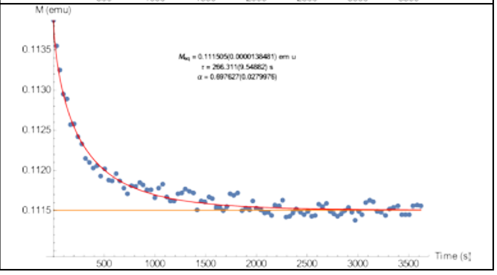

[a] M_{eq} fixed to theoretical value for $m_J = \pm 15/2$ doublet scaled to the experimental M_{sat} value at H_i = 50 kOe.

Supplementary Table 17. DC decay data for **1a** measured at 5 K.

H_{ext} (Oe)	M_{eq} (emu)	ΔM_{eq} (emu)	τ (s)	$\Delta\tau$ (s)	α	$\Delta\alpha$	Figure
19	0.000219 [a]		780	3	0.714	0.005	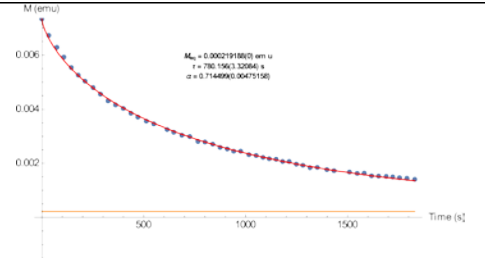
39	0.000458 [a]		648	2	0.694	0.003	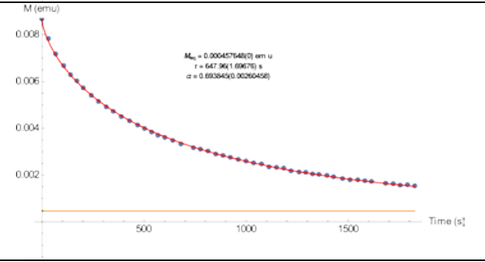
60	0.000695 [a]		690	2	0.748	0.004	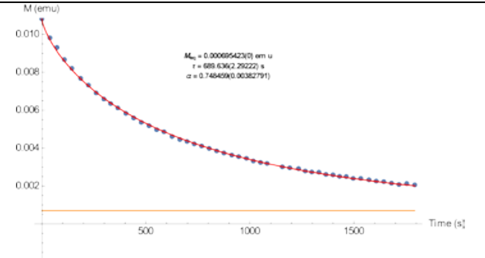
78	0.000909 [a]		779	2	0.752	0.003	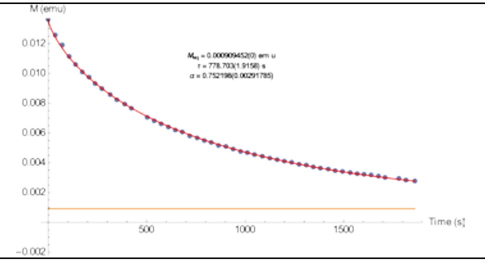
98	0.001146 [a]		914	3	0.779	0.004	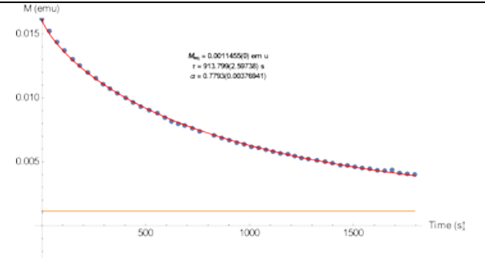
119	0.001388 [a]		1009	2	0.765	0.002	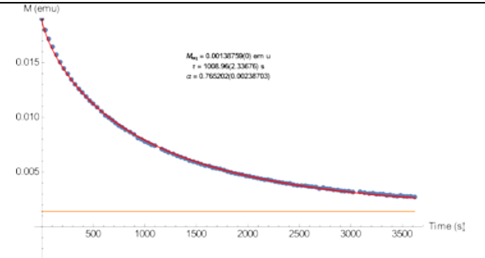
139	0.001623 [a]		1080	2	0.771	0.002	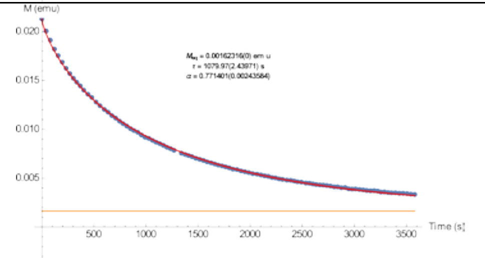

160	0.001860 [a]		1138	3	0.780	0.003	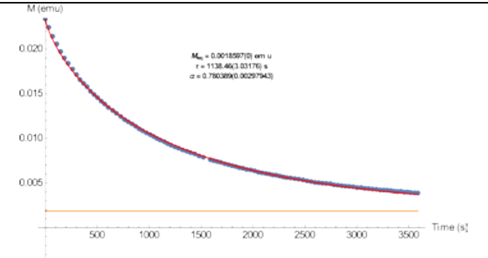
180	0.002099 [a]		1174	2	0.782	0.002	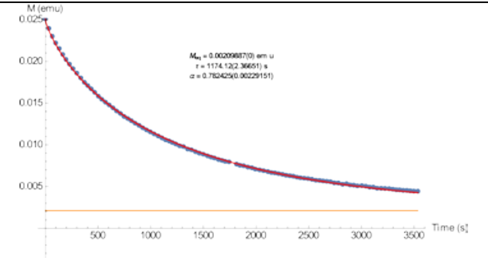
201	0.002338 [a]		1199	3	0.787	0.003	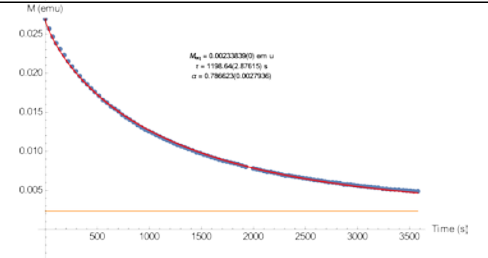
250	0.002902	0.000014	1257	3	0.776	0.003	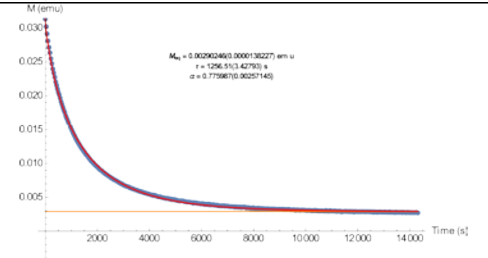
302	0.003501	0.000016	1284	3	0.777	0.002	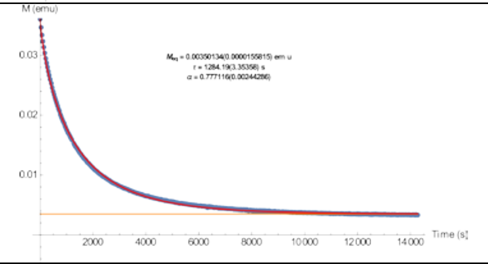
353	0.004060	0.000016	1298	3	0.767	0.002	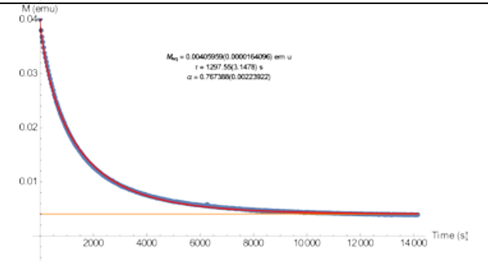
402	0.004689	0.000024	1356	4	0.801	0.003	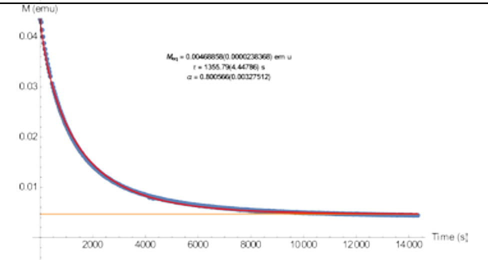

452	0.005250	0.000025	1361	4	0.795	0.003	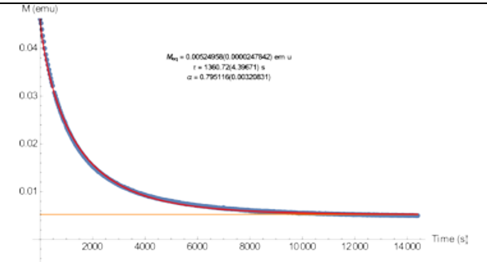
502	0.005837	0.000027	1377	5	0.802	0.003	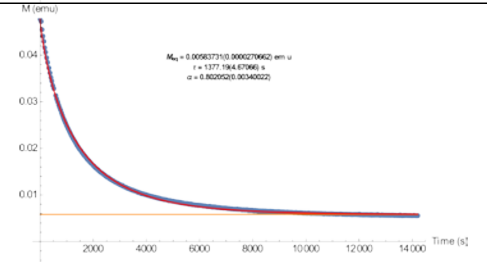
1006	0.010877	0.000019	1403	4	0.781	0.003	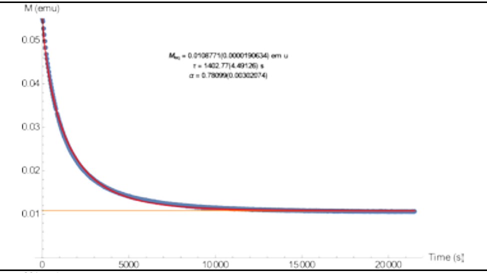
2012	0.021198	0.000013	1267	3	0.750	0.002	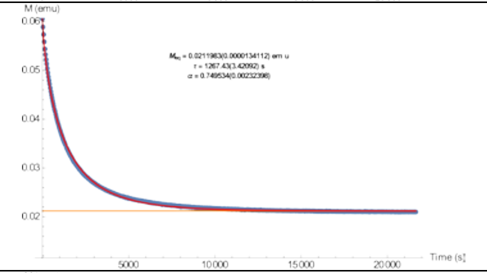
3018	0.031123	0.000010	1135	3	0.749	0.002	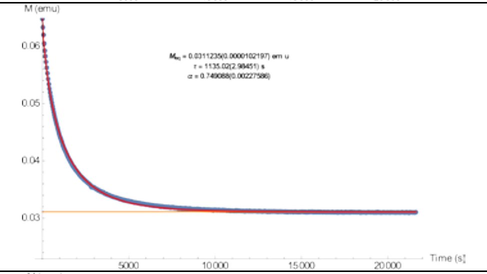
4025	0.040625	0.000007	957	2	0.753	0.002	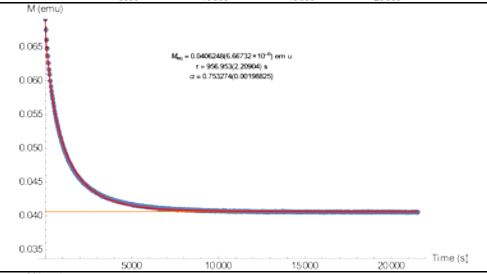
5031	0.049547	0.000009	827	3	0.808	0.004	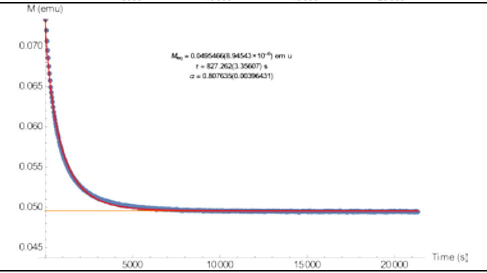

5532	0.053829	0.000016	730	4	0.835	0.006	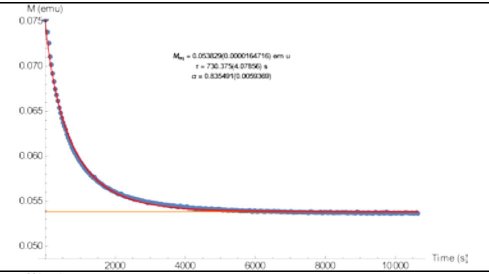
6036	0.057721	0.000013	639	3	0.810	0.005	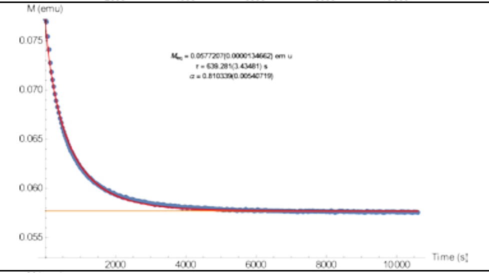
6539	0.061482	0.000012	574	3	0.833	0.006	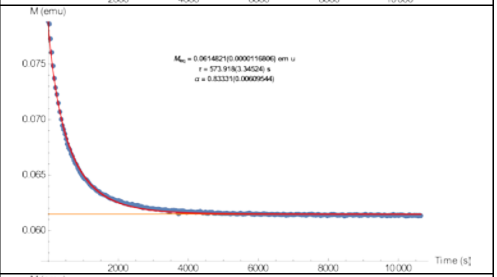
7041	0.065086	0.000011	512	3	0.837	0.007	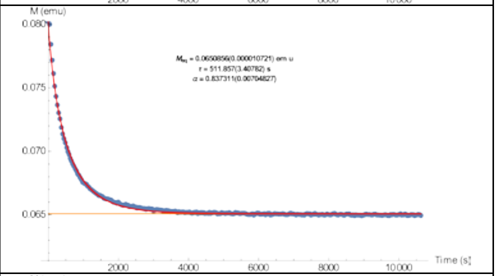
7544	0.068588	0.000009	449	3	0.849	0.007	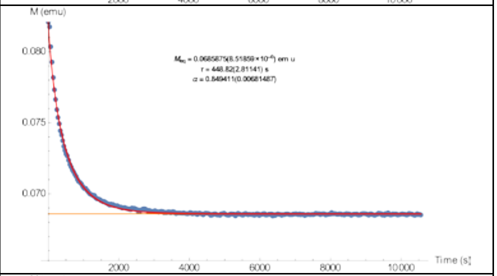
8048	0.071978	0.000009	435	3	0.900	0.009	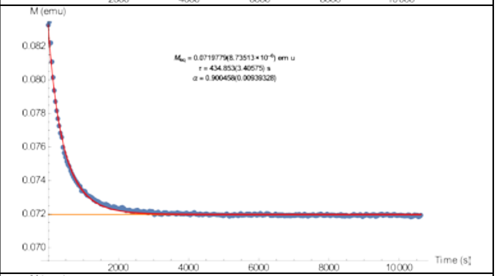
9053	0.078113	0.000012	310	3	0.799	0.009	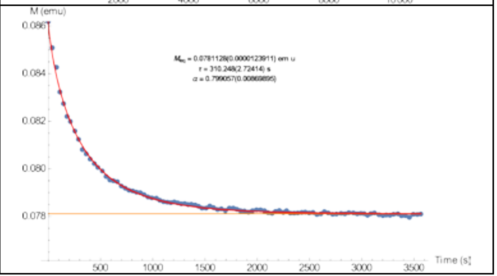

10059	0.083537	0.000009	270	3	0.789	0.009	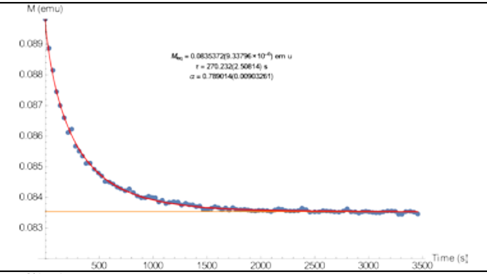
11064	0.088358	0.000008	225	3	0.782	0.012	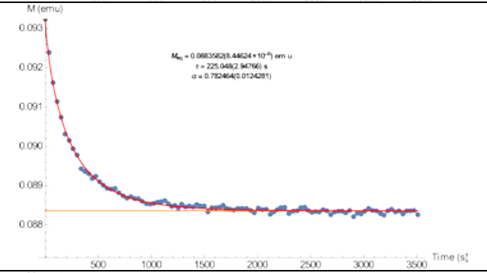
12069	0.092677	0.000008	179	3	0.729	0.015	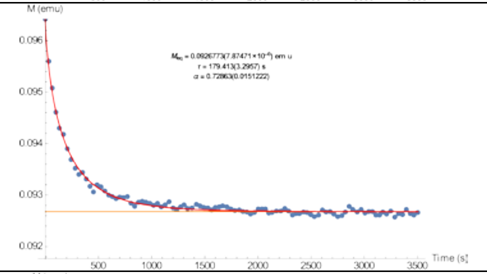
13076	0.096495	0.000011	195	6	0.698	0.024	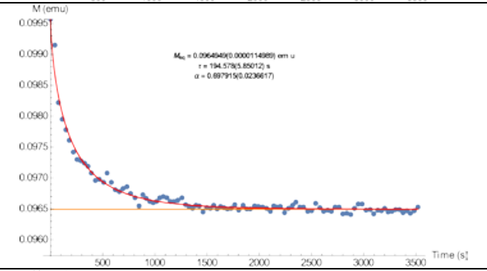
14079	0.099884	0.000007	167	5	0.712	0.022	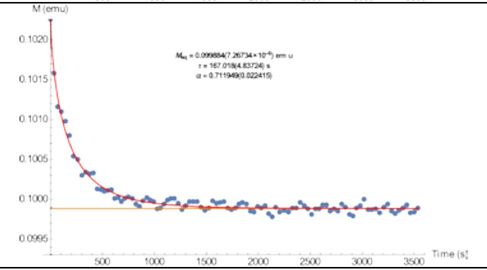
15085	0.102864	0.000010	191	7	0.615	0.024	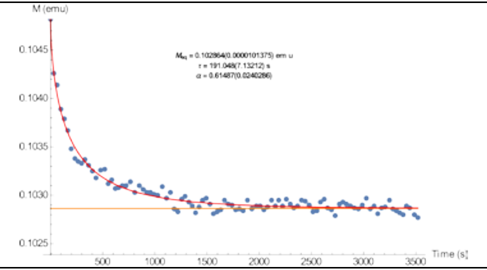

[a] M_{eq} fixed to theoretical value for $m_J = \pm 15/2$ doublet scaled to the experimental M_{sat} value at $H_i = 50$ kOe.

Supplementary Table 18. DC decay data for **1a** measured at 6 K.

H_{ext} (Oe)	M_{eq} (emu)	ΔM_{eq} (emu)	τ (s)	$\Delta\tau$ (s)	α	$\Delta\alpha$	Figure
21	0.000169	0.000011	324	3	0.785	0.007	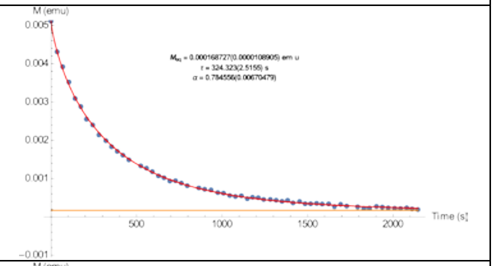
41	0.000330	0.000011	289	2	0.779	0.007	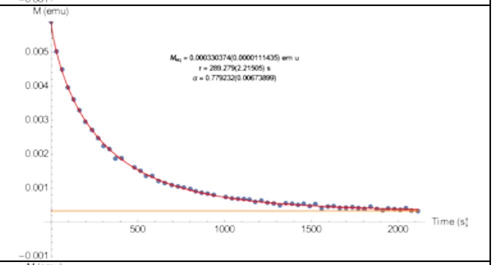
62	0.000549	0.000012	299	2	0.833	0.006	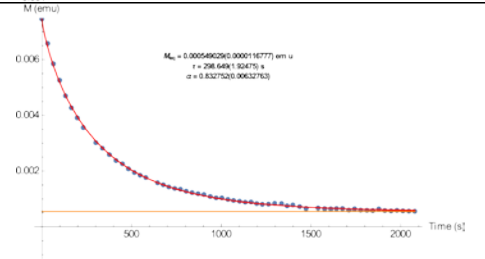
80	0.000785	0.000021	346	3	0.879	0.010	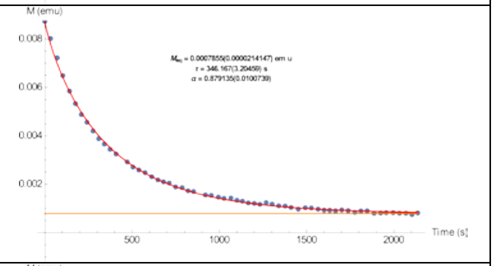
101	0.000975 [a]		357	1	0.827	0.004	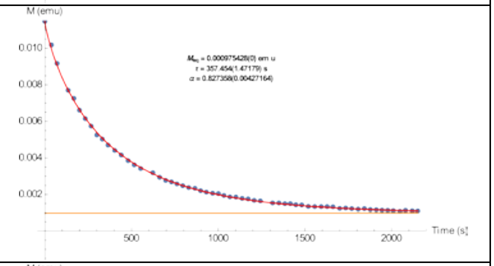
121	0.001175 [a]		387	2	0.832	0.005	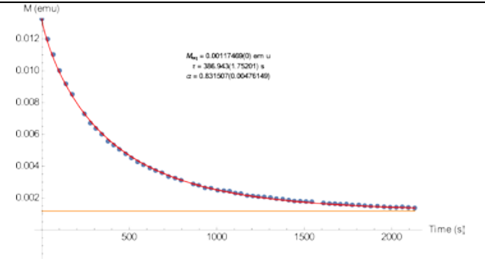
142	0.001372 [a]		408	2	0.830	0.004	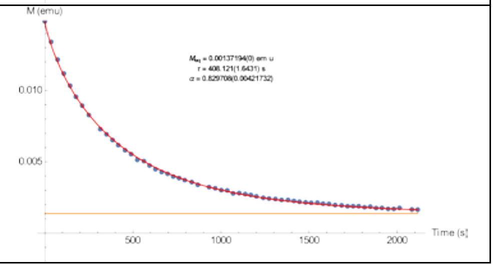

162	0.001569 [a]		424	2	0.831	0.005	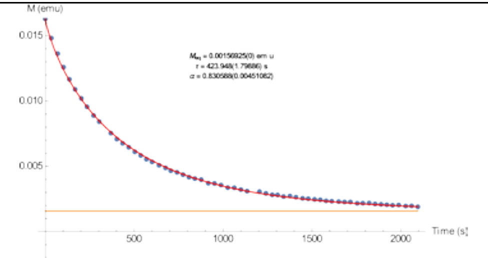
182	0.001768 [a]		431	2	0.830	0.005	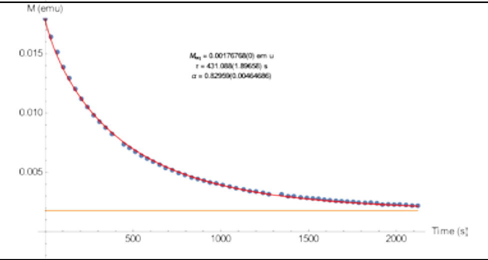
203	0.001965 [a]		451	2	0.845	0.005	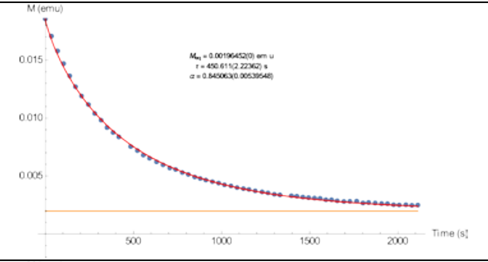
252	0.002418	0.000018	447	2	0.821	0.004	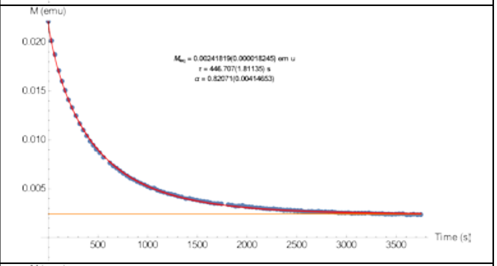
304	0.002965	0.000025	460	2	0.842	0.005	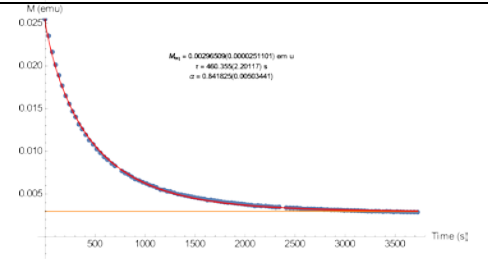
354	0.003453	0.000028	471	2	0.846	0.005	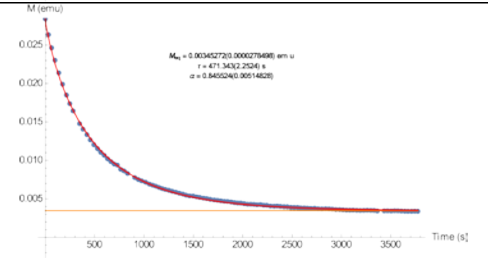
404	0.003901	0.000029	478	2	0.839	0.005	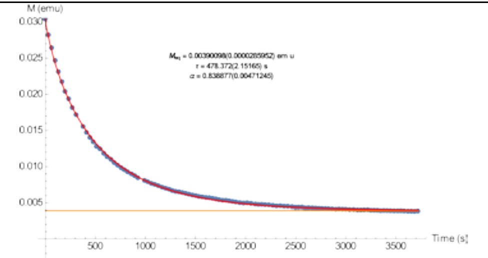

455	0.004472	0.000040	491	3	0.874	0.006	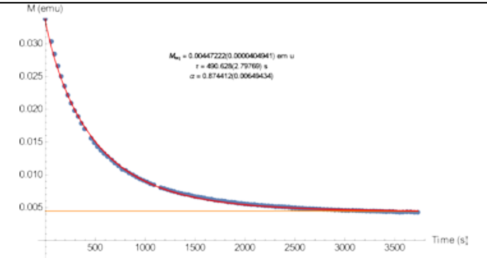
504	0.004933	0.000044	491	3	0.873	0.007	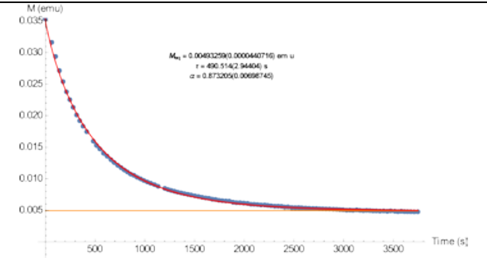
1007	0.009007	0.000023	509	3	0.836	0.006	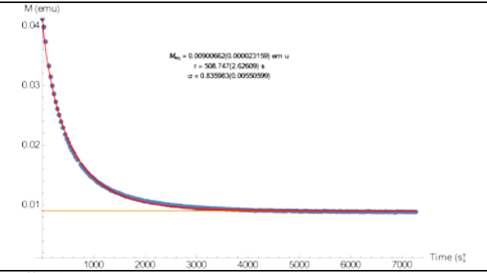
2014	0.017720	0.000021	490	3	0.835	0.006	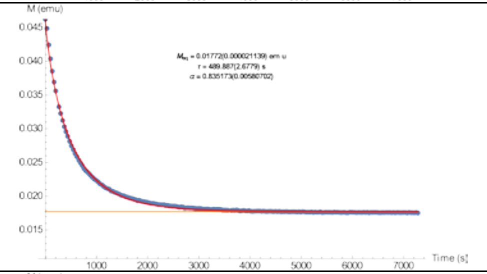
3020	0.026165	0.000012	449	2	0.796	0.004	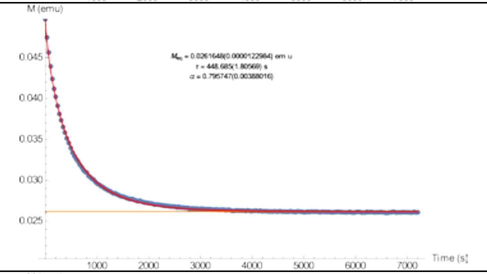
4026	0.034333	0.000010	414	2	0.800	0.004	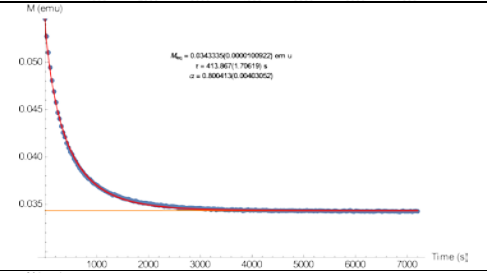
5032	0.042130	0.000008	366	2	0.802	0.004	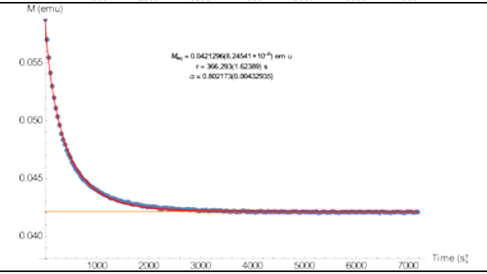

5533	0.045956	0.000015	341	2	0.824	0.006	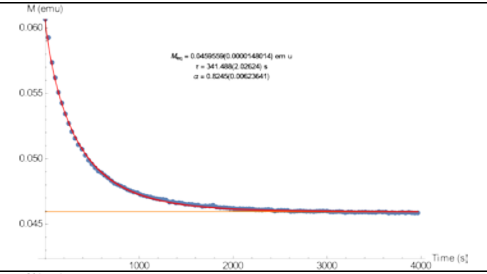
6037	0.049626	0.000021	334	3	0.904	0.011	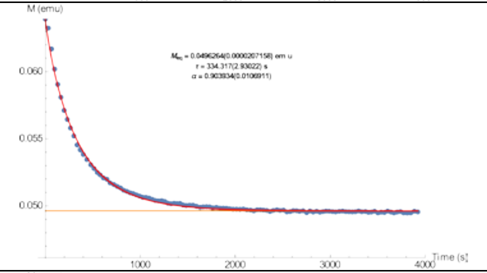
6540	0.053106	0.000019	318	3	0.914	0.012	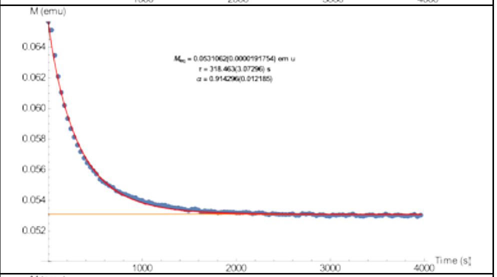
7044	0.056481	0.000016	284	3	0.899	0.012	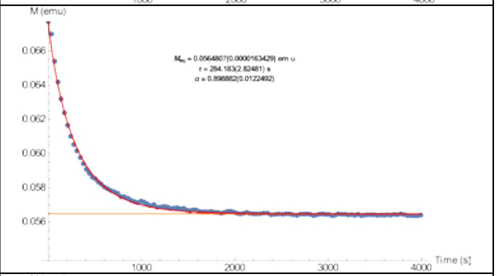
7546	0.059731	0.000016	272	3	0.928	0.015	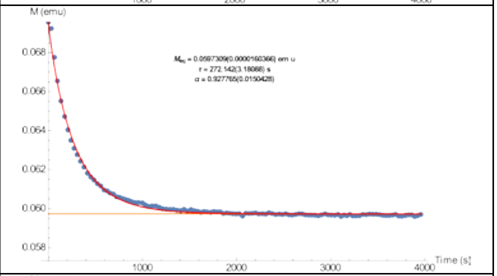
8050	0.062866	0.000015	268	3	0.993	0.019	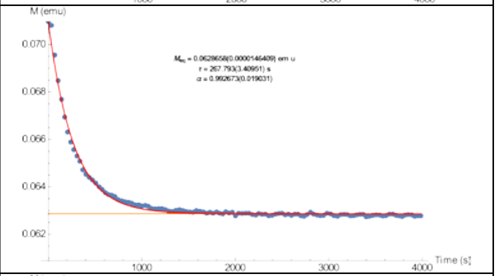
9055	0.068748	0.000015	197	2	0.832	0.013	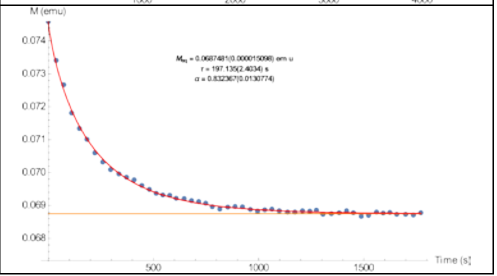

10061	0.074170	0.000014	164	3	0.827	0.019	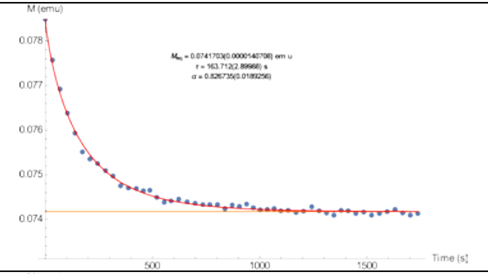
11066	0.079055	0.000011	171	3	0.805	0.018	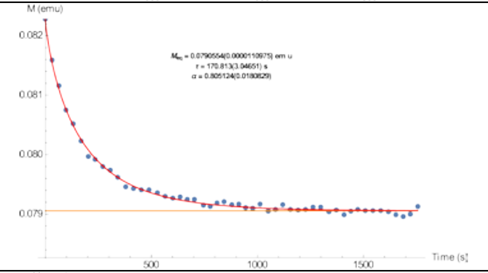
12071	0.083541	0.000012	147	5	0.888	0.038	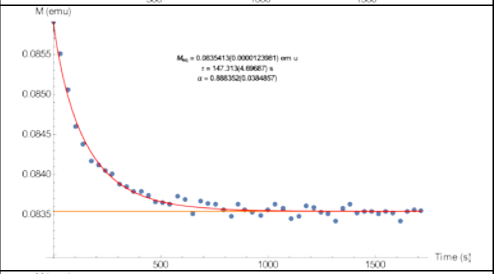
13077	0.087631	0.000013	124	5	0.707	0.036	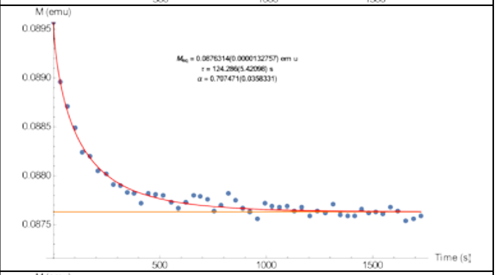
14080	0.091274	0.000017	183	10	0.879	0.067	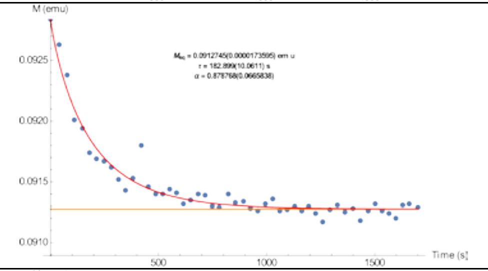
15086	0.094607	0.000009	146	6	0.898	0.052	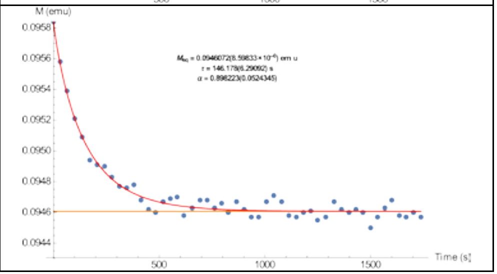

[a] M_{eq} fixed to theoretical value for $m_J = \pm 15/2$ doublet scaled to the experimental M_{sat} value at H_i = 50 kOe.

Supplementary Table 19. DC decay data for **1a** measured at 7 K.

***H*$_{ext}$ (Oe)**	***M*$_{eq}$ (emu)**	***ΔM*$_{eq}$ (emu)**	***τ* (s)**	***Δτ* (s)**	***α***	***Δα***	**Figure**
20	0.000103	0.000012	202	4	0.937	0.026	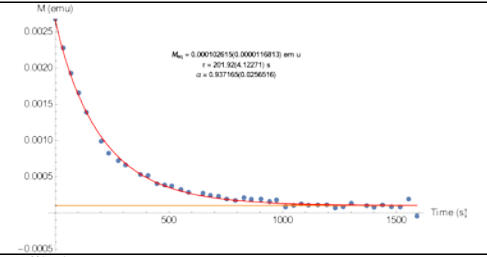
40	0.000247	0.000010	162	3	0.886	0.020	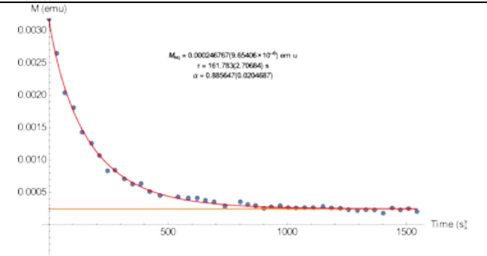
61	0.000399	0.000007	161	2	0.864	0.011	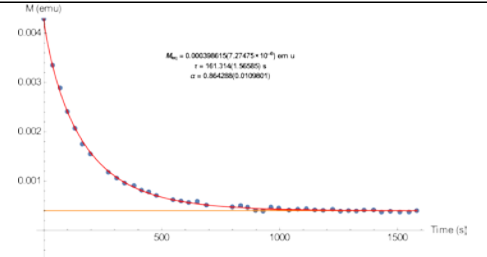
80	0.000566	0.000011	169	2	0.866	0.013	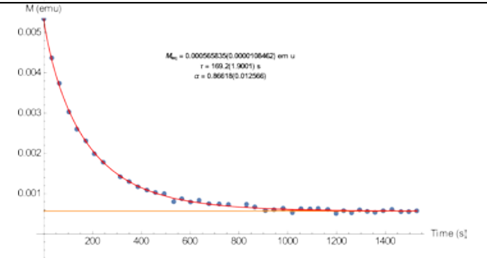
100	0.000734	0.000012	180	2	0.853	0.011	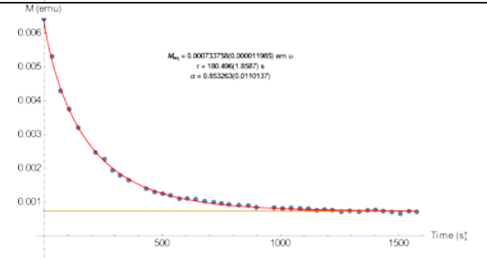
120	0.000915	0.000039	192	4	0.876	0.020	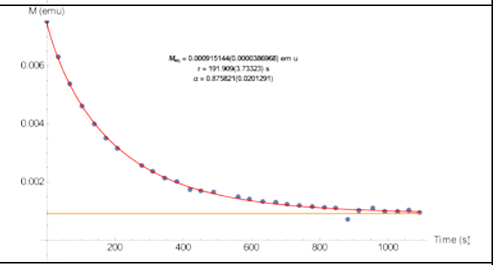
141	0.001166	0.000023	196	2	0.893	0.012	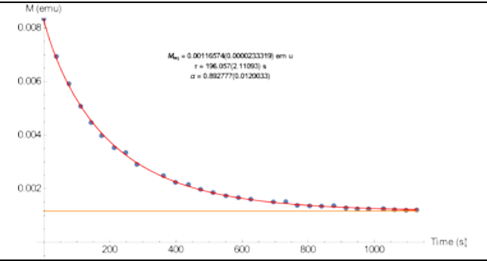

161	0.001317	0.000036	199	3	0.864	0.014	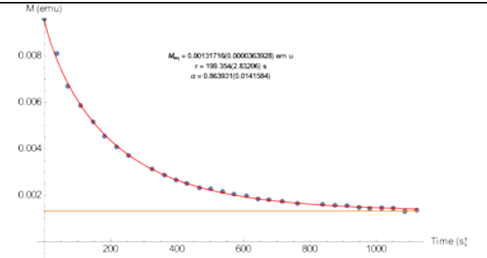
181	0.001535	0.000028	212	2	0.920	0.011	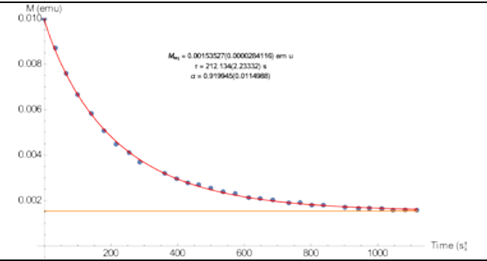
202	0.001696	0.000028	208	2	0.888	0.009	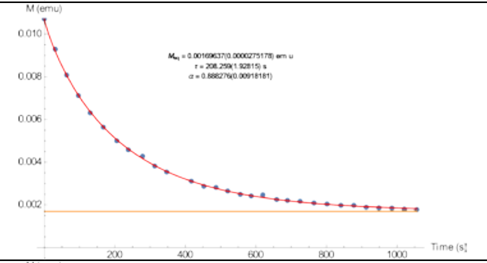
251	0.001873	0.000009	221	2	0.826	0.007	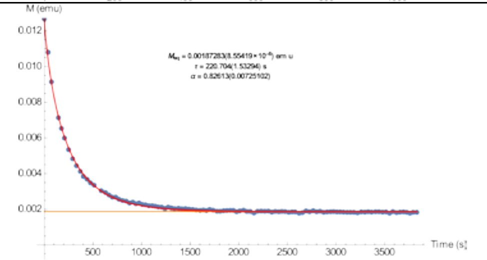
302	0.002274	0.000008	222	1	0.833	0.006	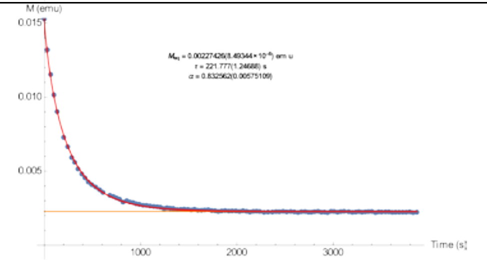
353	0.002667	0.000010	228	1	0.846	0.006	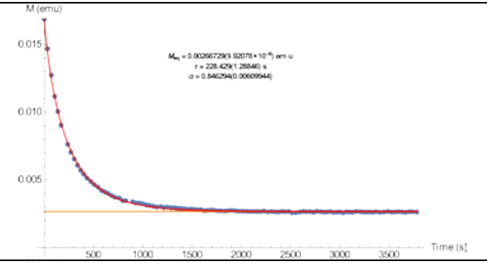
402	0.003059	0.000023	231	3	0.841	0.014	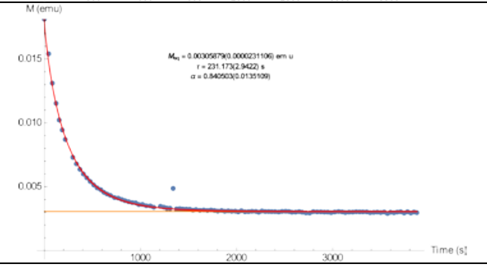

453	0.003427	0.000009	228	1	0.819	0.005	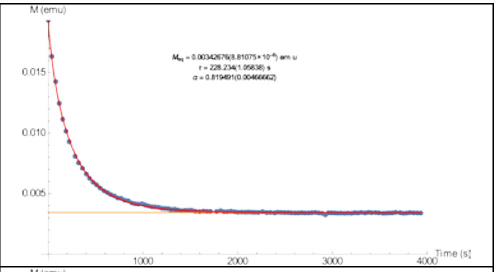
503	0.003793	0.000009	230	1	0.818	0.005	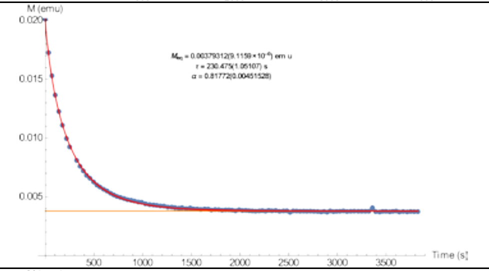
1006	0.007583	0.000005	241	1	0.825	0.004	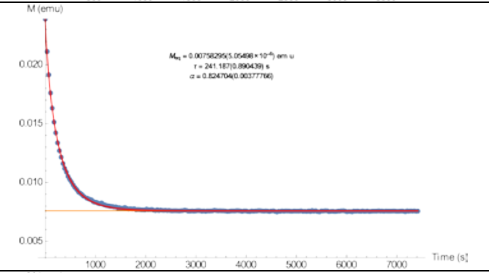
2012	0.015115	0.000004	231	1	0.817	0.003	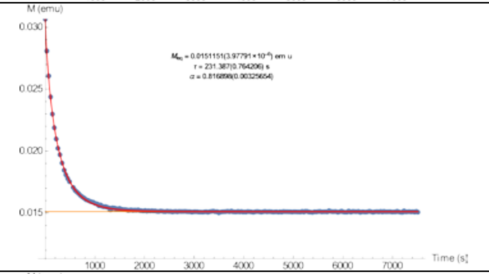
3019	0.022502	0.000005	224	1	0.814	0.004	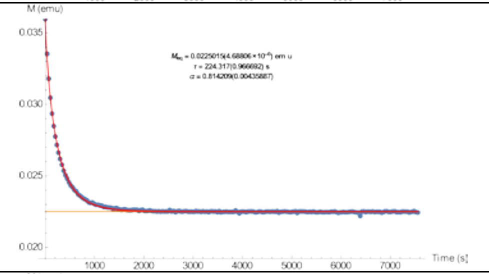
4025	0.029692	0.000005	212	1	0.833	0.006	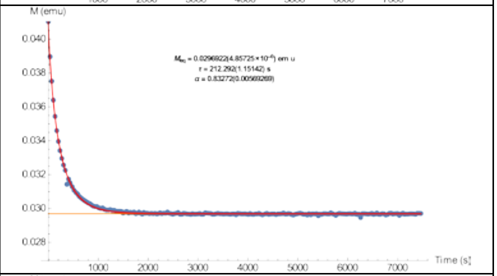
5031	0.036641	0.000003	198	1	0.817	0.005	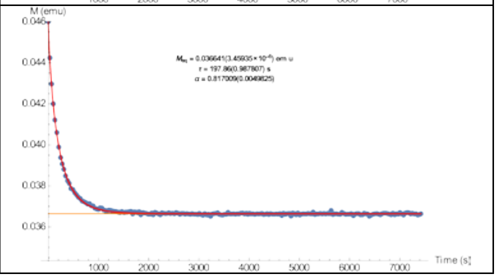

5533	0.040017	0.000006	193	1	0.828	0.006	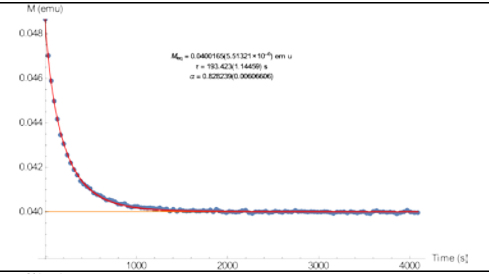
6037	0.043294	0.000006	188	1	0.852	0.008	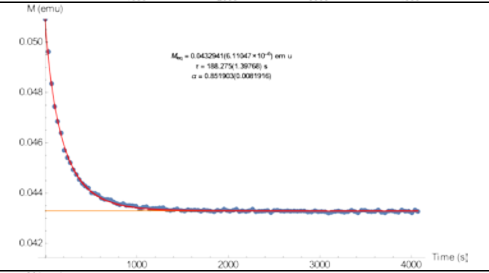
6539	0.046490	0.000006	168	2	0.814	0.010	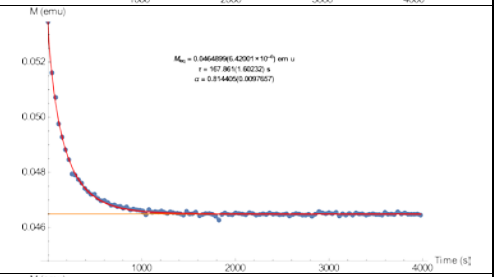
7043	0.049608	0.000005	164	1	0.837	0.009	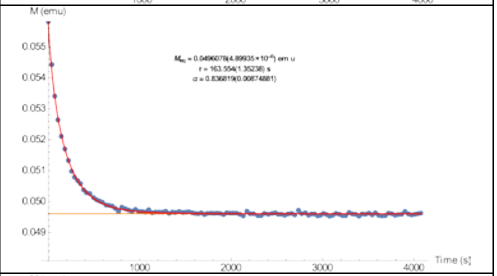
7545	0.052577	0.000006	168	2	0.868	0.012	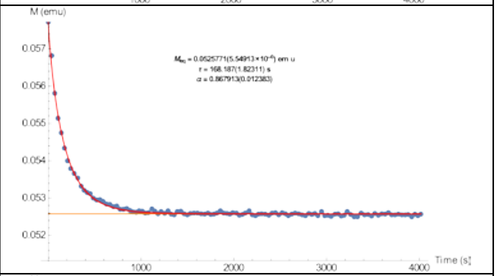
8048	0.055470	0.000004	160	2	0.785	0.011	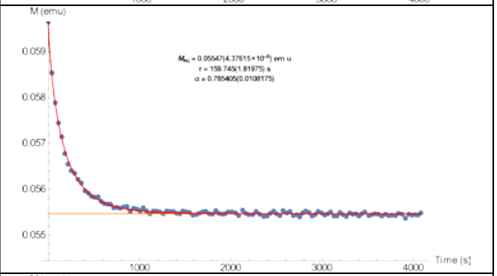
9053	0.061014	0.000010	142	3	0.779	0.018	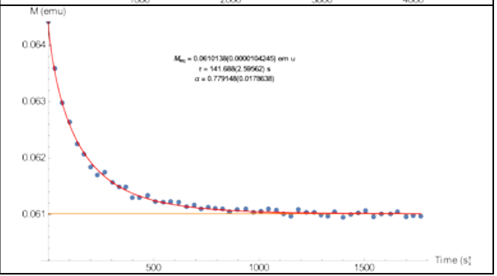

10059	0.066183	0.000009	122	3	0.760	0.025	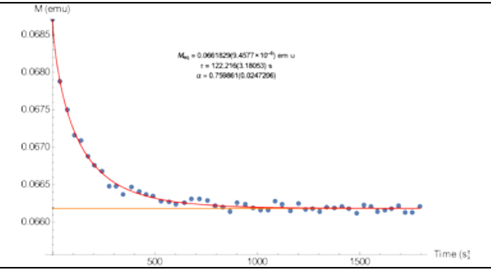
11063	0.071009	0.000009	128	4	0.852	0.035	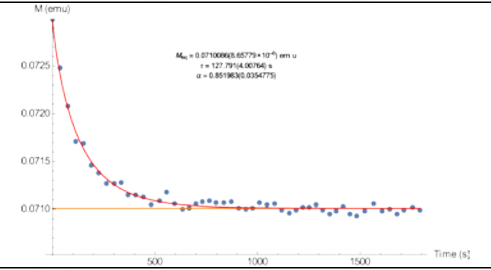
12069	0.075491	0.000009	93	4	0.685	0.034	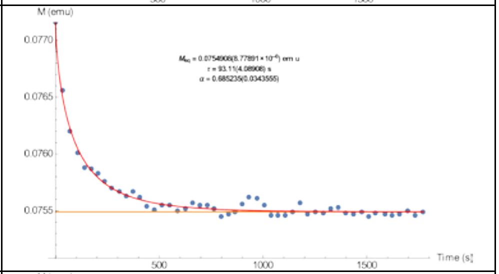
13075	0.079571	0.000019	116	8	0.578	0.042	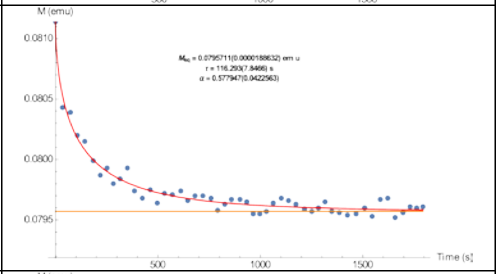
14078	0.083419	0.000009	157	10	0.879	0.076	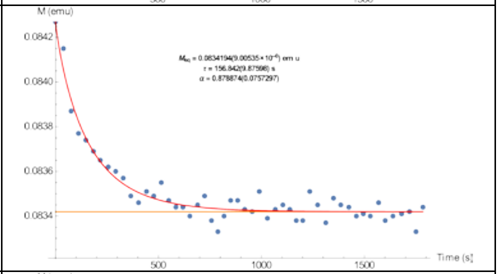
15084	0.086880	0.000022	149	12	0.569	0.047	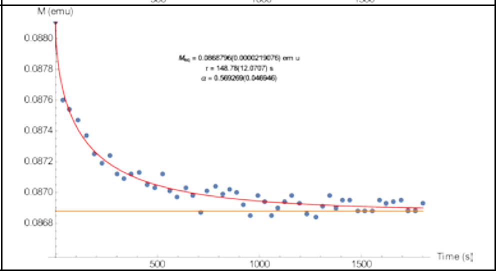

Supplementary Table 20. DC decay data for **1a** measured at 8 K.

***H*$_{ext}$ (Oe)**	***M*$_{eq}$ (emu)**	***ΔM*$_{eq}$ (emu)**	***τ* *(s)***	***Δτ* *(s)***	***α***	***Δα***	**Figure**
21	0.000042	0.000015	144	7	0.878	0.050	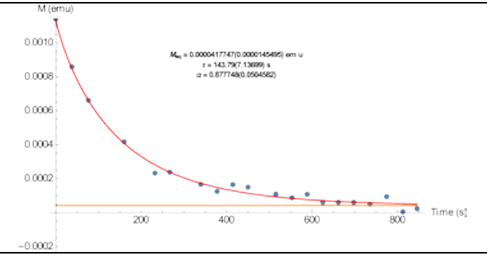
41	0.000191	0.000019	116	6	0.876	0.058	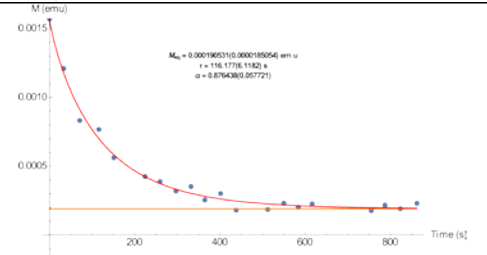
62	0.000348	0.000008	121	3	1.062	0.034	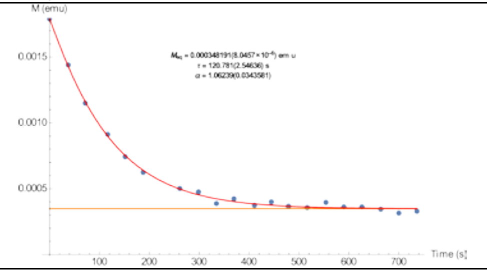
80	0.000454	0.000019	104	4	0.828	0.039	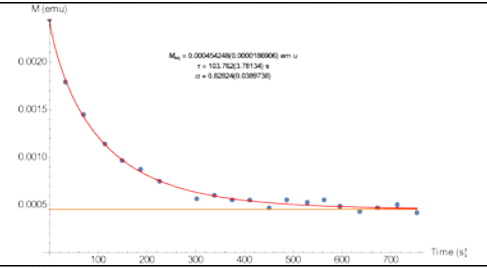
100	0.000583	0.000031	114	5	0.841	0.053	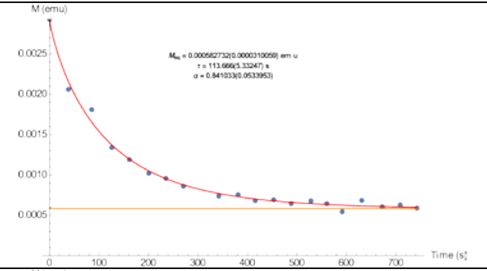
121	0.000737	0.000017	121	4	0.916	0.041	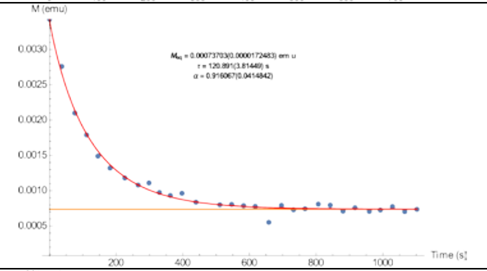
141	0.000882	0.000014	120	3	0.889	0.026	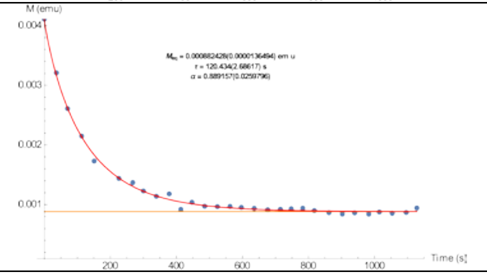

162	0.001035	0.000012	127	2	0.901	0.019	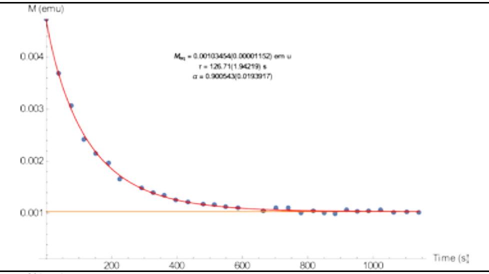
182	0.001177	0.000014	123	2	0.884	0.023	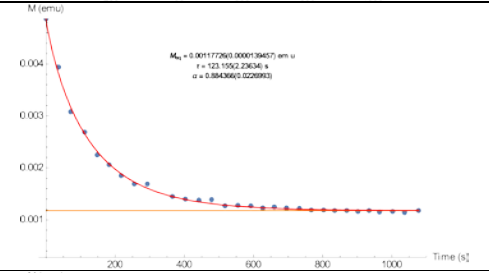
203	0.001266	0.000013	108	2	0.735	0.014	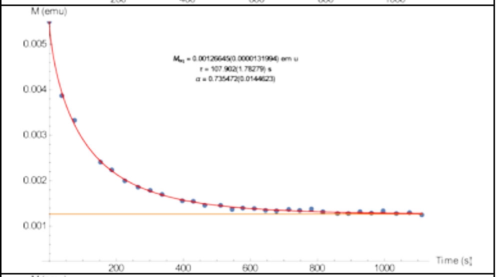
252	0.001628	0.000006	131	1	0.869	0.011	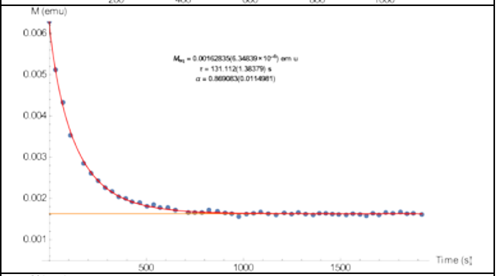
303	0.001977	0.000007	139	1	0.893	0.012	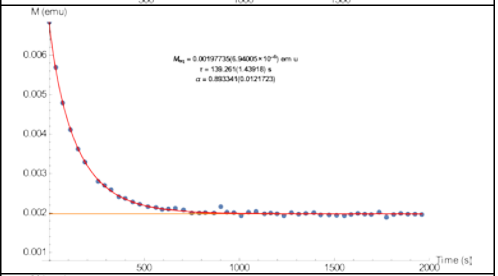
354	0.002335	0.000011	134	2	0.894	0.016	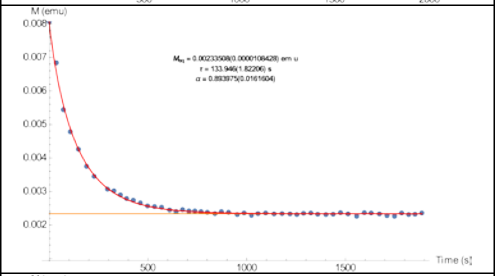
404	0.002655	0.000008	127	1	0.835	0.010	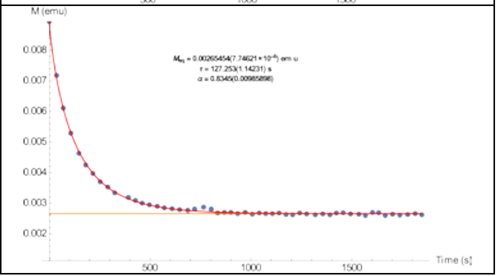

455	0.003021	0.000012	129	2	0.867	0.015	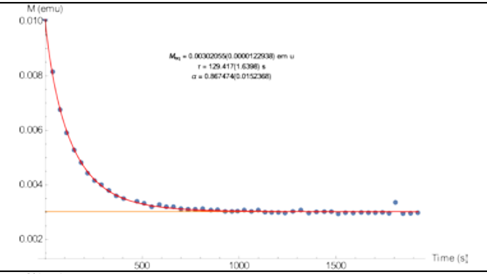
504	0.003318	0.000006	128	1	0.810	0.006	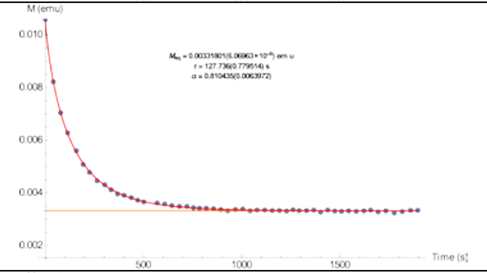
1007	0.006656	0.000004	136	1	0.827	0.006	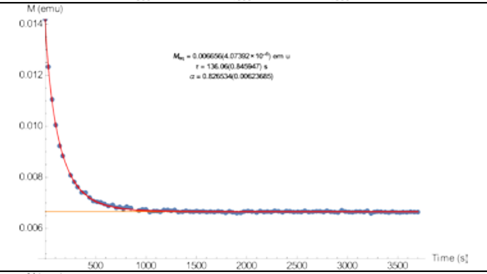
2014	0.013304	0.000004	136	1	0.848	0.006	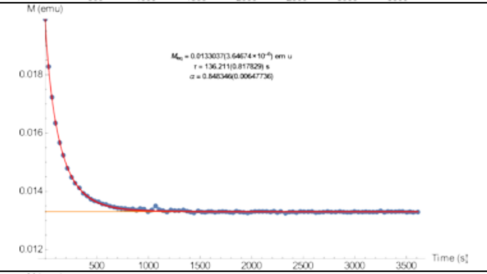
3020	0.019822	0.000005	144	1	0.880	0.012	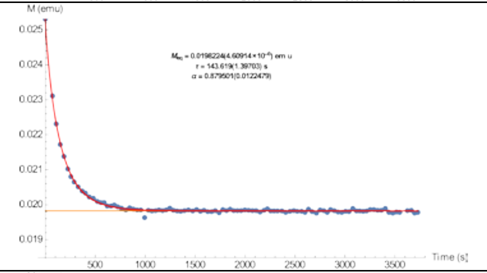
4026	0.026213	0.000005	136	1	0.859	0.012	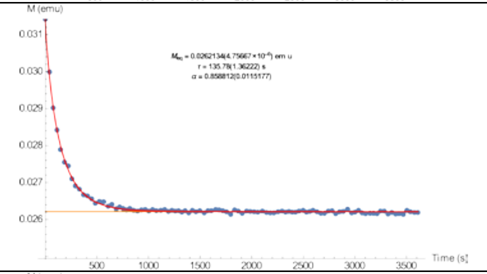
5032	0.032390	0.000005	131	2	0.816	0.013	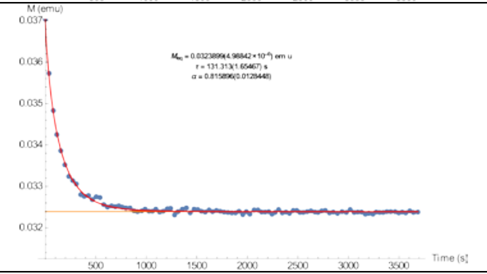

5533	0.035414	0.000007	130	2	0.898	0.019	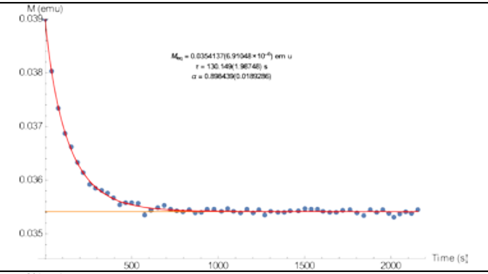
6037	0.038364	0.000008	130	2	0.849	0.020	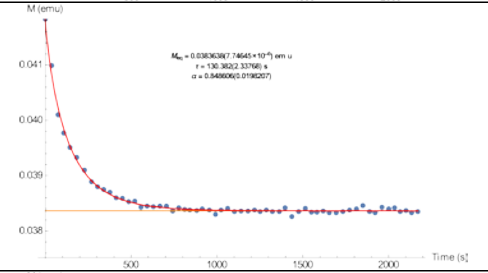
6540	0.041270	0.000008	122	2	0.835	0.022	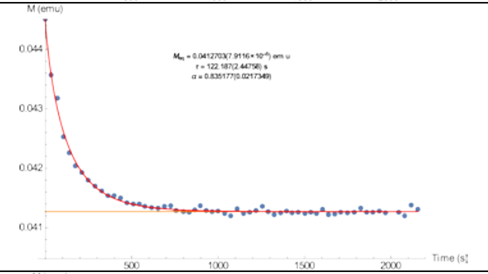
7043	0.044114	0.000006	118	2	0.778	0.017	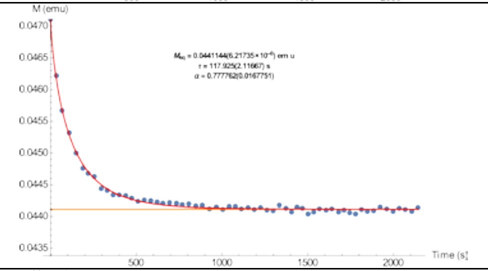
7546	0.046917	0.000010	97	4	0.765	0.034	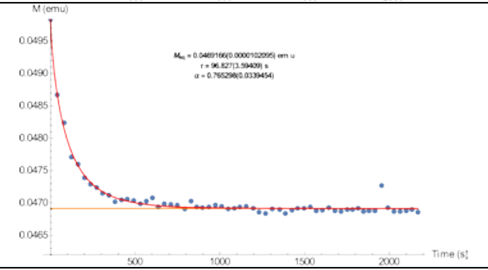
8050	0.049655	0.000006	112	3	0.909	0.031	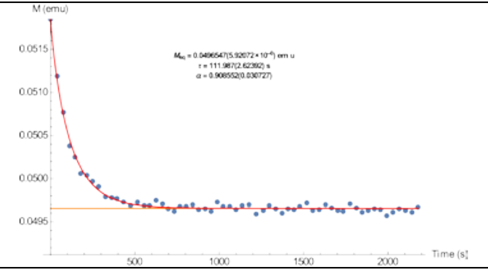
9055	0.054871	0.000016	147	9	0.980	0.083	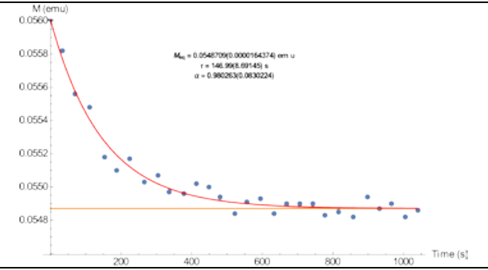

Supplementary Table 21. DC decay data for **1a** measured at 9 K.

H_{ext} (Oe)	M_{eq} (emu)	ΔM_{eq} (emu)	τ (s)	$\Delta\tau$ (s)	α	$\Delta\alpha$	Figure
80	0.000400	0.000006	98	4	1.098	0.070	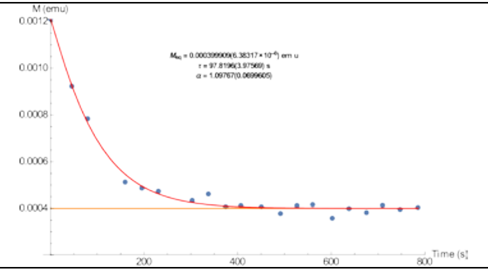
100	0.000516	0.000012	85	5	0.828	0.071	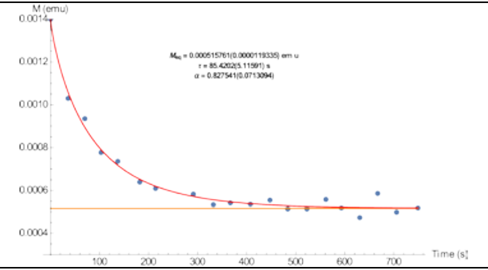
120	0.000650	0.000009	91	5	0.963	0.082	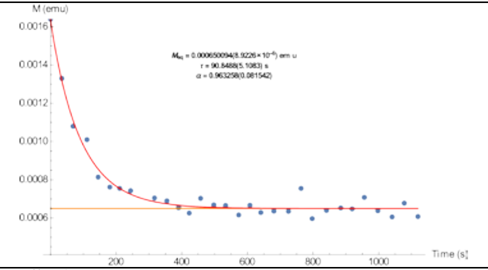
141	0.000778	0.000010	78	5	0.859	0.078	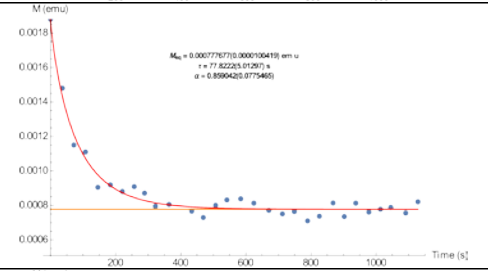
161	0.000904	0.000009	83	4	0.959	0.072	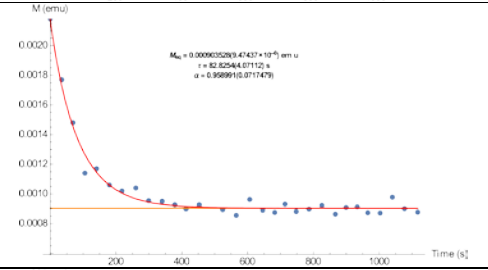
181	0.001021	0.000007	120	6	1.094	0.079	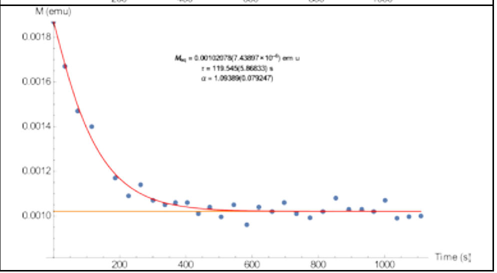

202	0.001147	0.000006	81	2	0.904	0.033	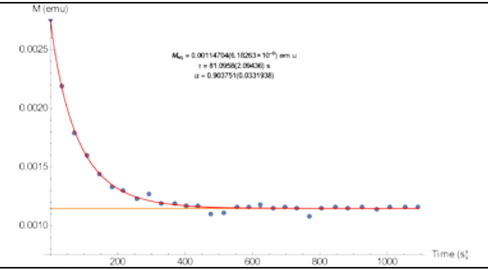
251	0.001436	0.000006	81	3	0.788	0.035	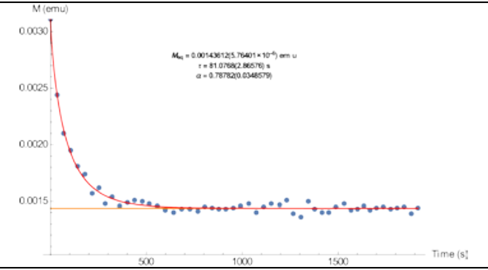
303	0.001756	0.000005	74	3	0.757	0.041	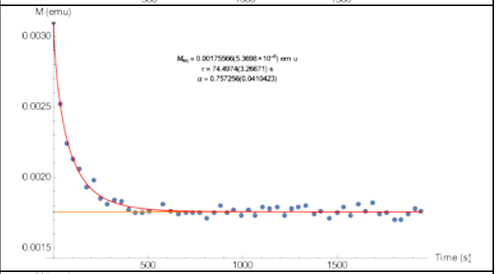
354	0.002059	0.000007	96	3	1.080	0.061	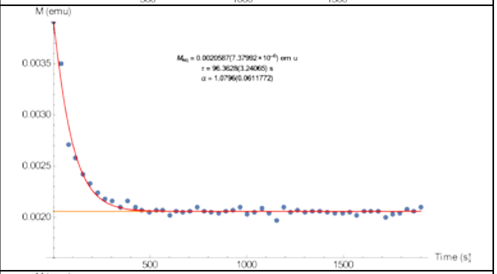
403	0.002361	0.000005	89	2	0.913	0.030	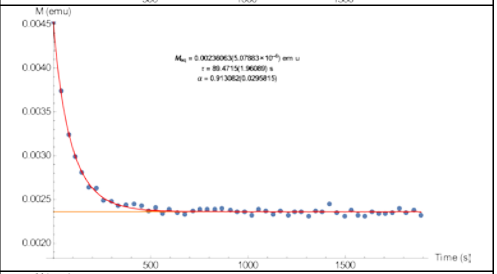
454	0.002662	0.000007	98	3	0.923	0.035	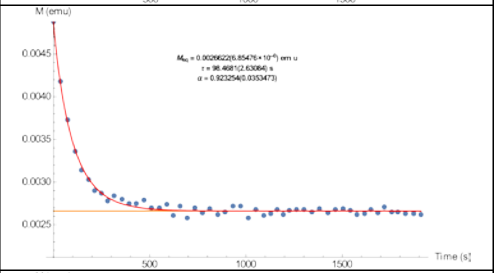
504	0.002961	0.000005	101	2	0.948	0.029	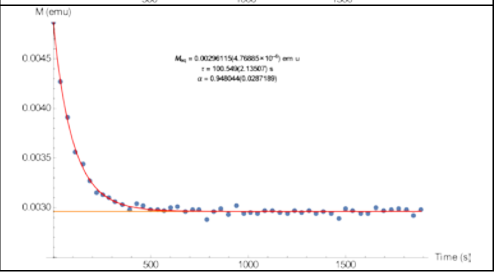

1007	0.005941	0.000003	91	2	0.869	0.023	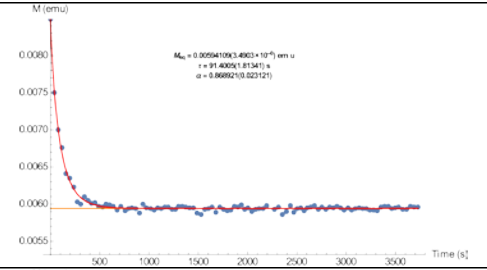
2014	0.011885	0.000004	79	2	0.772	0.023	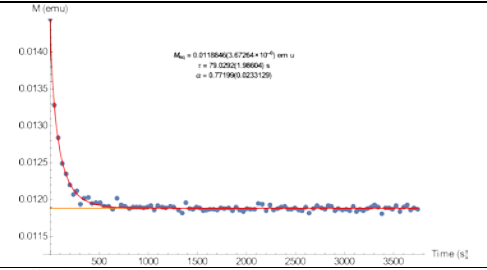
3020	0.017738	0.000003	93	2	0.842	0.025	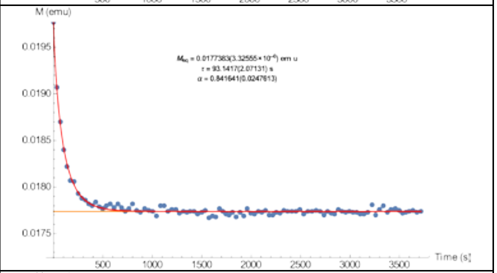
4026	0.023468	0.000003	85	2	0.839	0.028	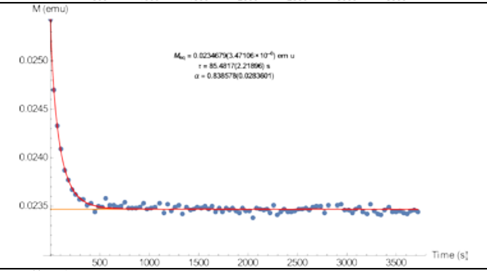
5031	0.029067	0.000004	99	3	0.937	0.044	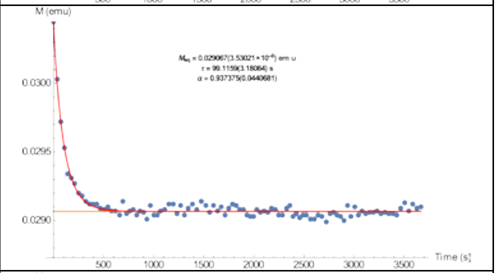
5533	0.031787	0.000006	100	4	0.716	0.034	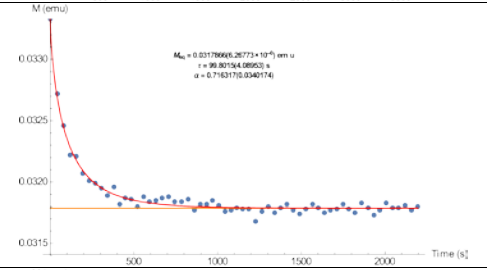
6036	0.034504	0.000007	81	4	0.751	0.045	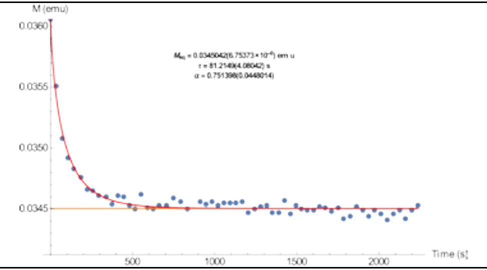

6539	0.037141	0.000006	109	5	0.894	0.059	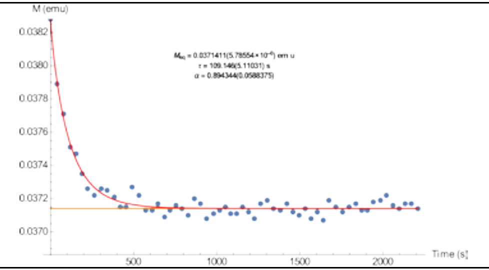
7042	0.039745	0.000010	120	8	0.787	0.065	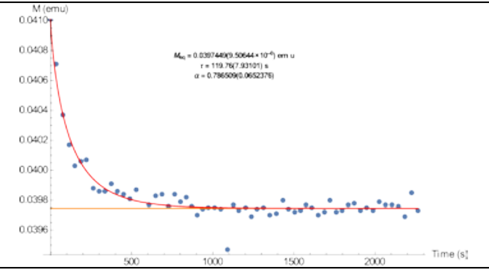
7545	0.042293	0.000010	129	8	0.637	0.041	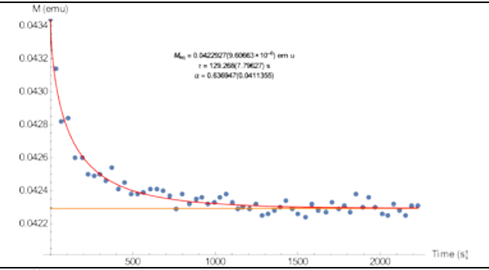
8048	0.044817	0.000008	107	7	0.814	0.068	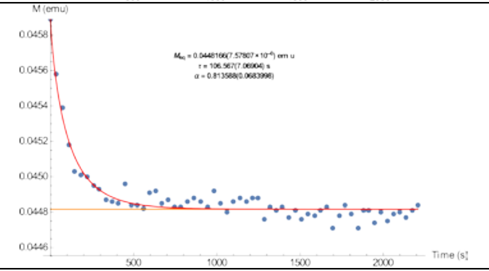

Supplementary Table 22. DC decay data for **1b** measured at 2 K.

H_{ext} (Oe)	M_{eq} (emu)	τ (s)	$\Delta\tau$ (s)	α	$\Delta\alpha$	Figure
22	0.000622 [a]	4046	108	0.374	0.009	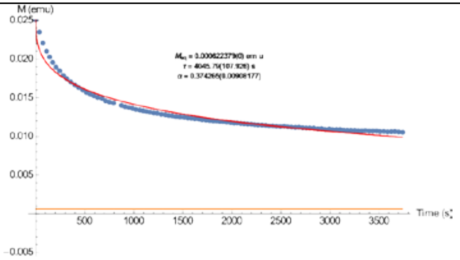
42	0.001209 [a]	15283	543	0.457	0.008	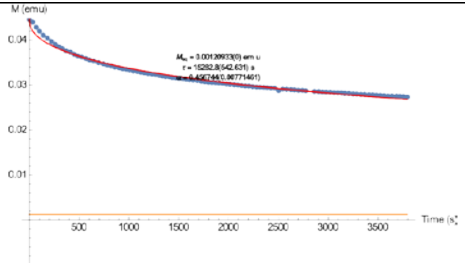
62	0.001792 [a]	77509	6150	0.371	0.008	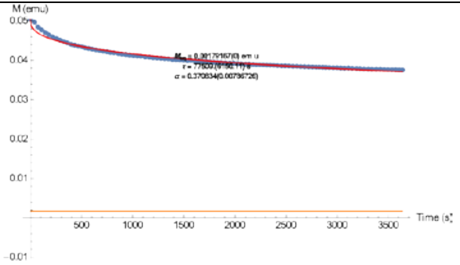
81	0.002343 [a]	180048	20961	0.360	0.009	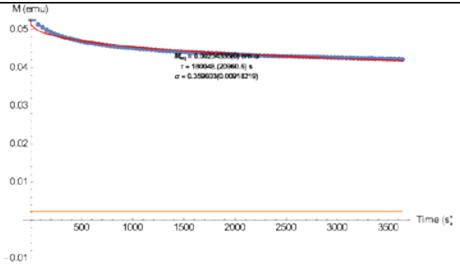
101	0.002925 [a]	469877	63419	0.322	0.008	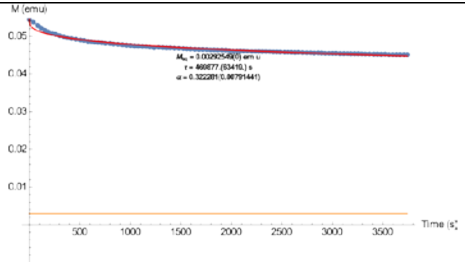
122	0.003518 [a]	710650	107683	0.312	0.008	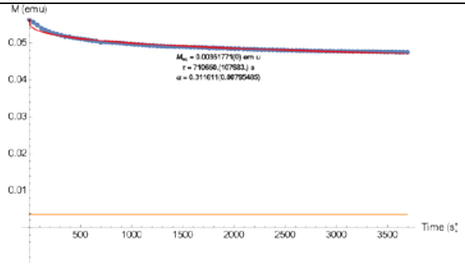

142	0.004107 [a]	468561	60948	0.339	0.008	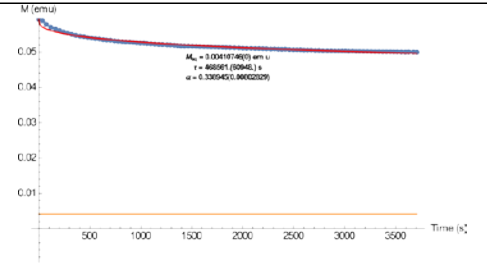
163	0.004698 [a]	620324	79155	0.318	0.007	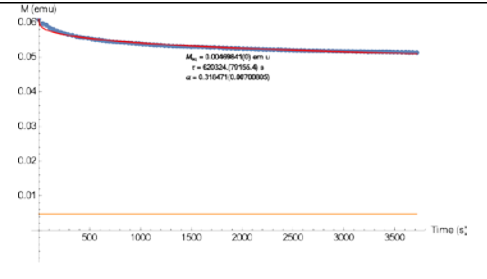
183	0.005283 [a]	574013	72727	0.337	0.007	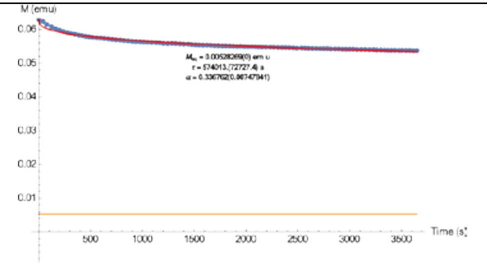
203	0.005872 [a]	3434063	159603	0.280	0.002	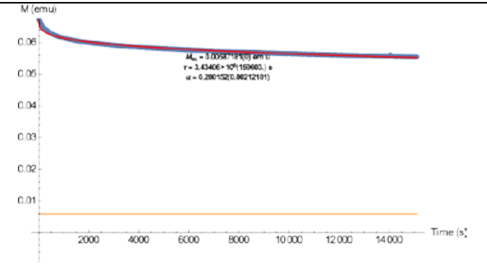
222	0.006412 [a]	4347192	174393	0.276	0.002	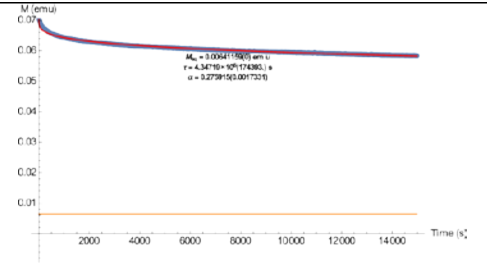
243	0.006997 [a]	3994706	162834	0.300	0.002	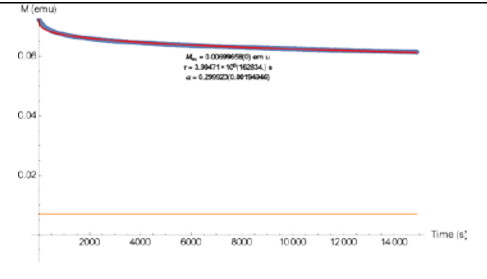
263	0.007582 [a]	4857589	164970	0.310	0.002	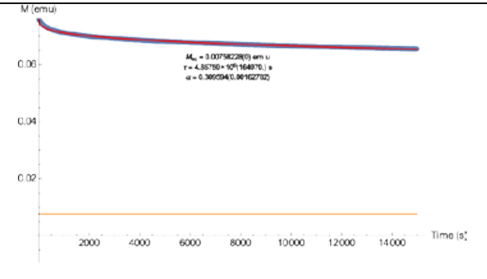

283	0.008173 [a]	4926110	146549	0.333	0.002	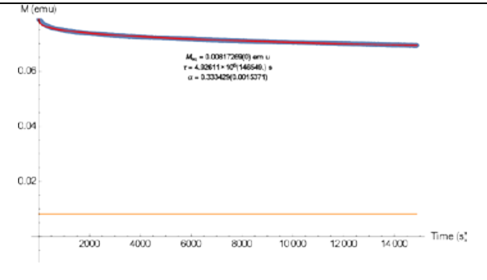
304	0.008746 [a]	4353599	126107	0.363	0.002	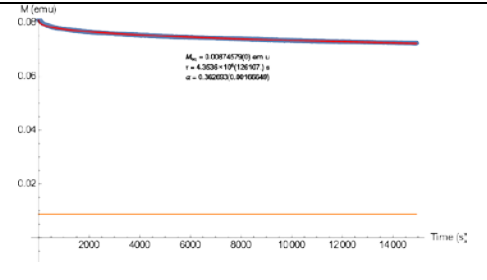
324	0.009337 [a]	5716525	110125	0.351	0.001	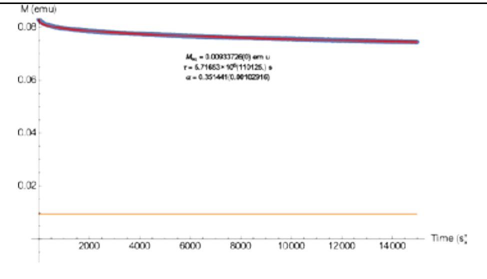
344	0.009928 [a]	4442744	94781	0.382	0.001	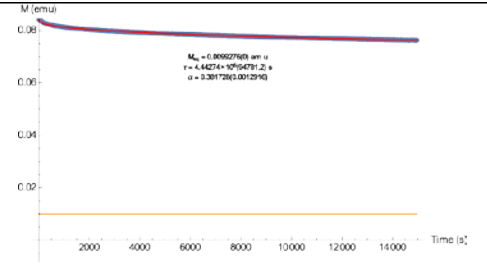
364	0.010472 [a]	2930215	77617	0.429	0.002	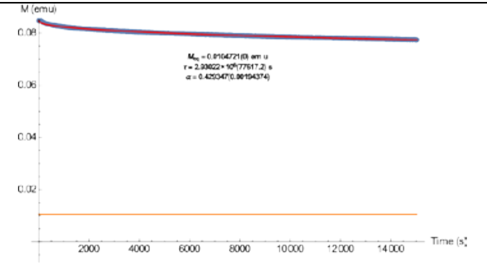
384	0.011057 [a]	2375768	63404	0.457	0.002	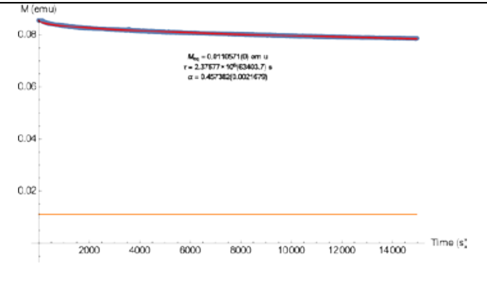
404	0.011628 [a]	2640075	65912	0.448	0.002	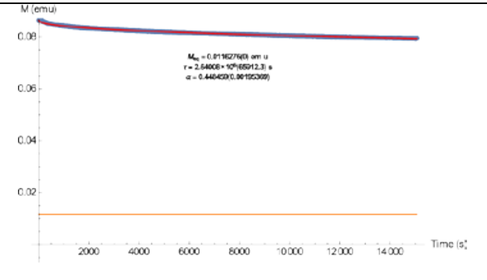

425	0.012225 [a]	5144209	60292	0.383	0.001	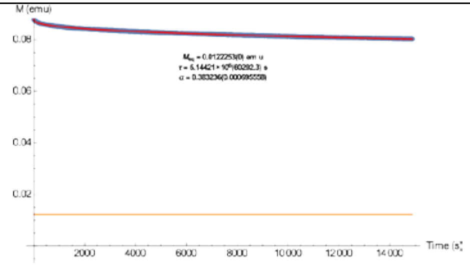
446	0.012817 [a]	3443722	61222	0.422	0.001	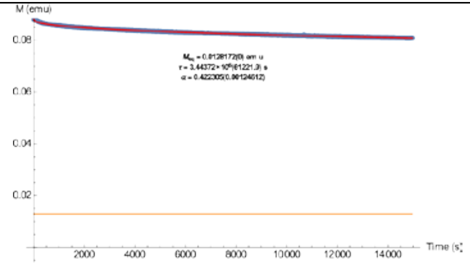
466	0.013393 [a]	4186225	53151	0.404	0.001	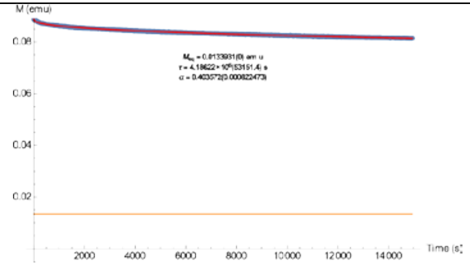
486	0.013945 [a]	2049238	52304	0.482	0.002	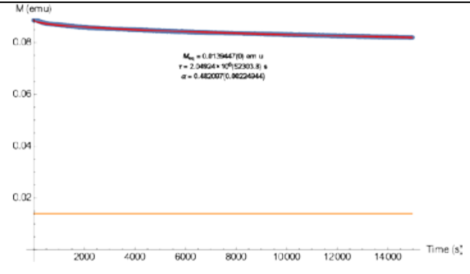
1008	0.028273 [a]	2602882	53509	0.440	0.001	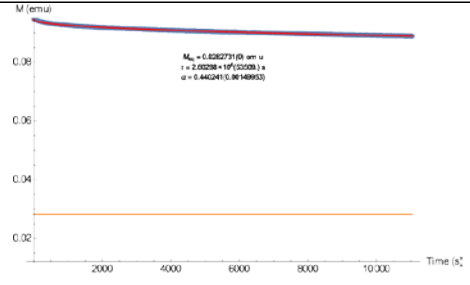
2014	0.053047 [a]	270591	2918	0.539	0.002	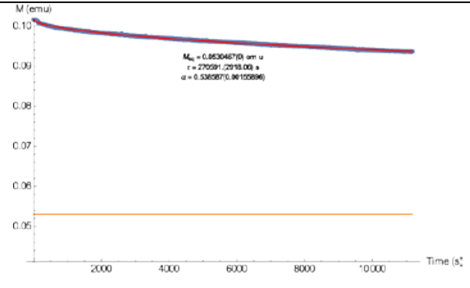
3021	0.072608 [a]	36565	314	0.681	0.003	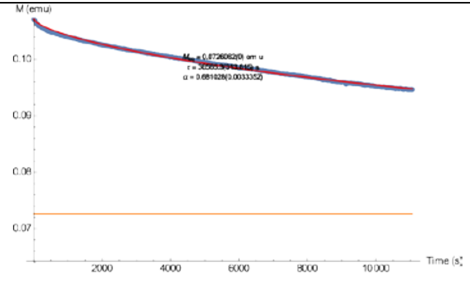

4027	0.087090 [a]	12383	14	0.691	0.001	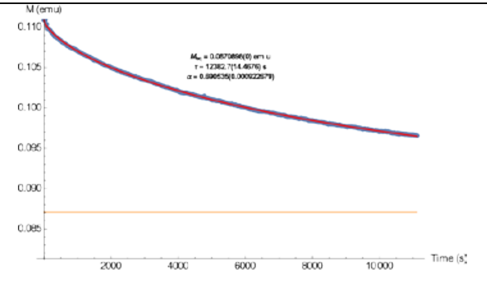
5034	0.097433 [a]	6678	23	0.651	0.004	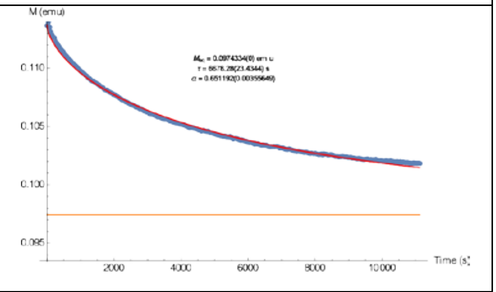
6038	0.104876 [a]	4539	29	0.575	0.005	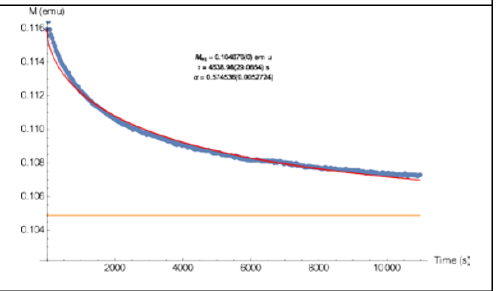
7045	0.110058 [a]	3358	31	0.491	0.006	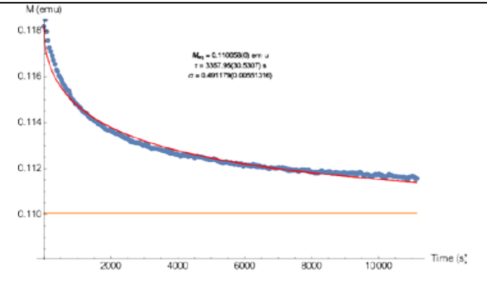
8050	0.114001 [a]	2399	26	0.478	0.005	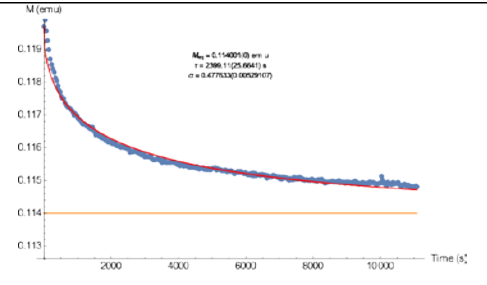
9056	0.116576 [a]	2414	33	0.501	0.009	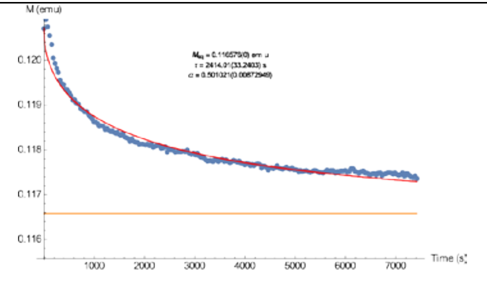

10061	0.118583 [a]	1914	34	0.443	0.009	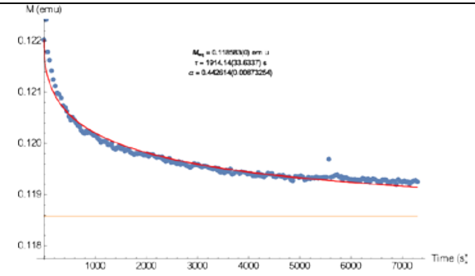
12072	0.121255 [a]	986	22	0.359	0.006	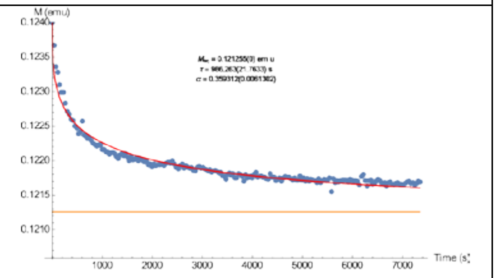
13078	0.122303 [a]	799	25	0.417	0.009	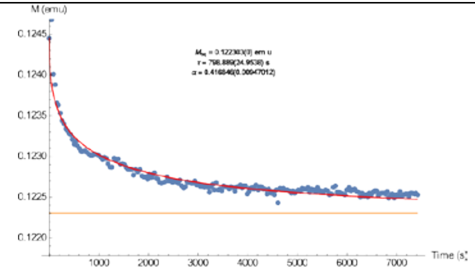

[a] M_{eq} fixed to theoretical value for m_J = ±15/2 doublet scaled to the experimental M_{sat} value at H_i = 50 kOe.

Supplementary Table 23. DC decay data for ~4% ^{164}Dy@**2** measured at 2 K.

H_{ext} (Oe)	M_{eq} (μ_B mol^{-1})	ΔM_{eq} (μ_B mol^{-1})	τ (s)	$\Delta\tau$ (s)	α	$\Delta\alpha$	Figure
10	0.000135 [a]		9165	79	0.335	0.003	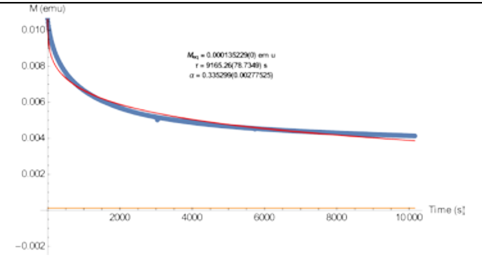
19	0.000257 [a]		16761	227	0.248	0.002	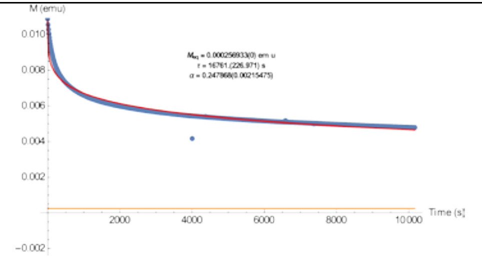
29	0.000392 [a]		109031	1859	0.232	0.001	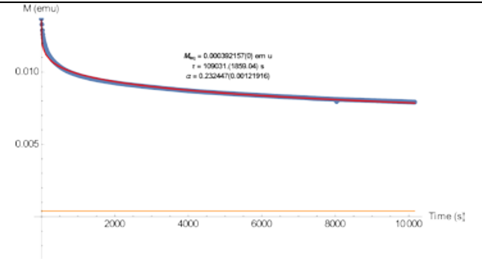
53	0.000717 [a]		2114309	60502	0.258	0.001	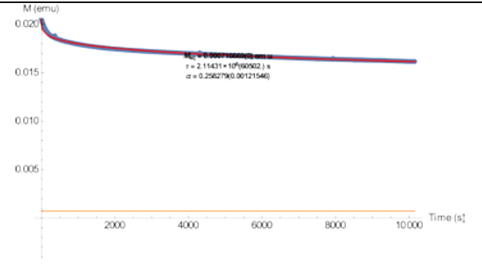
66	0.000892 [a]		2569436	74379	0.269	0.001	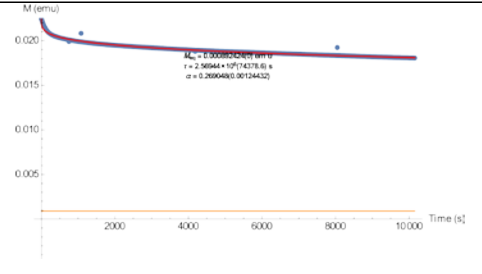
80	0.001082 [a]		3994133	113644	0.265	0.001	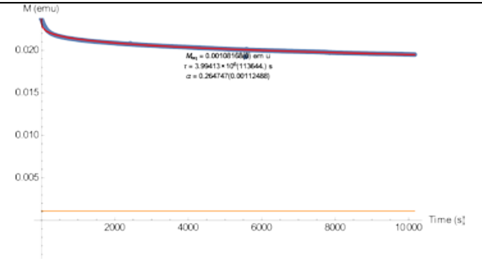

100	0.001352 [a]		2940545	91897	0.280	0.001	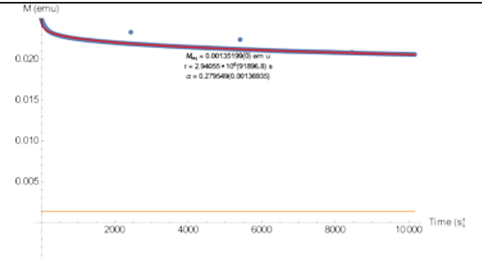
120	0.001622 [a]		872683	14872	0.339	0.001	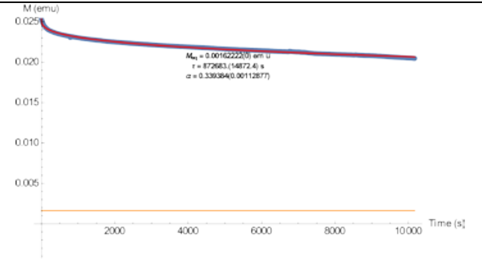
150	0.002027 [a]		783643	15283	0.320	0.001	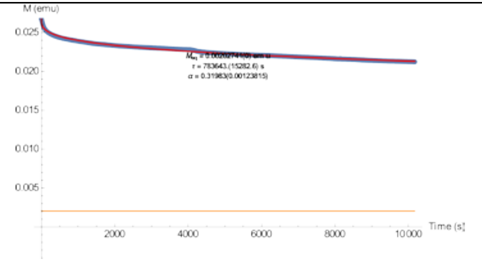
190	0.002567 [a]		624828	9386	0.332	0.001	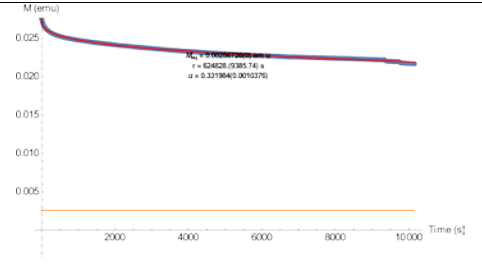
230	0.003107 [a]		1399367	29831	0.284	0.001	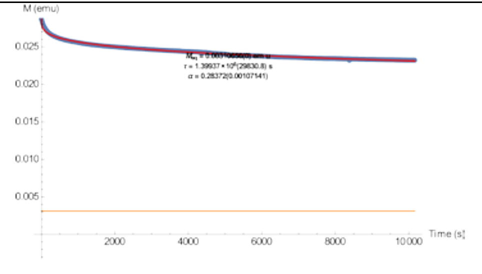
280	0.003780 [a]		2149252	75911	0.250	0.001	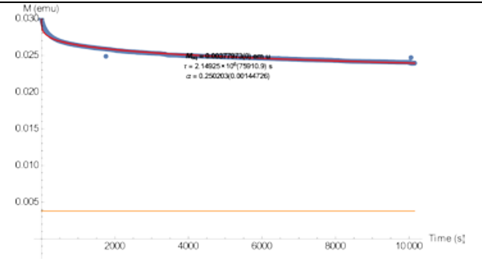
350	0.004720 [a]		1972544	70341	0.238	0.001	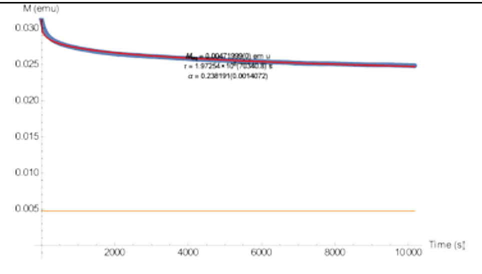

430	0.005791 [a]		1643905	64873	0.239	0.002	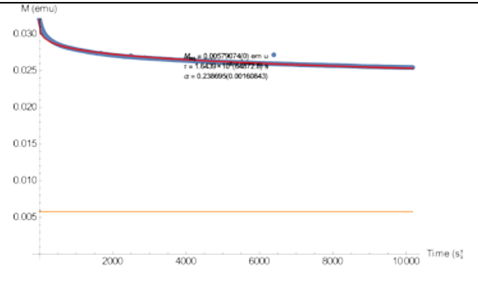
530	0.007122 [a]		1121432	41138	0.241	0.002	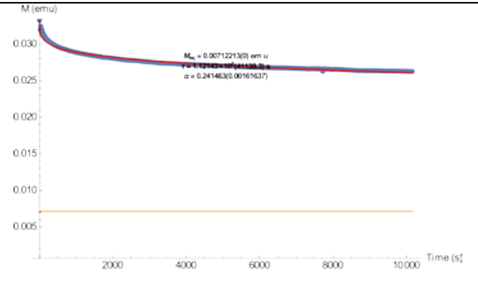
650	0.008707 [a]		704509	19508	0.263	0.001	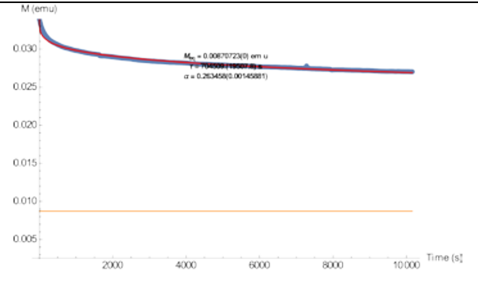
800	0.010665 [a]		684214	21708	0.251	0.002	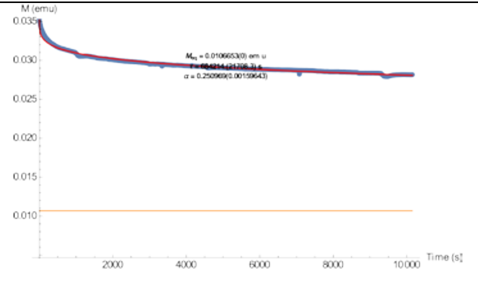
1000	0.013227 [a]		473699	22115	0.239	0.002	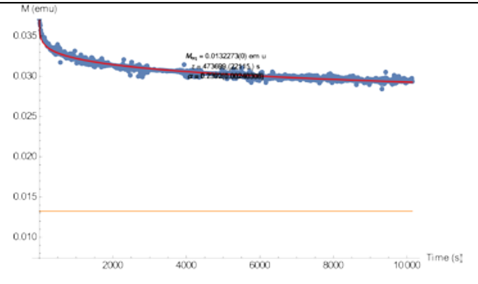
1200	0.015724 [a]		374071	12774	0.274	0.002	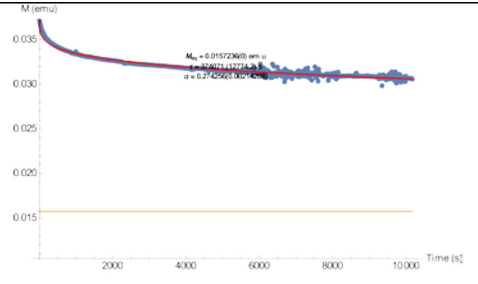

1500	0.019324 [a]		192345	3131	0.288	0.001	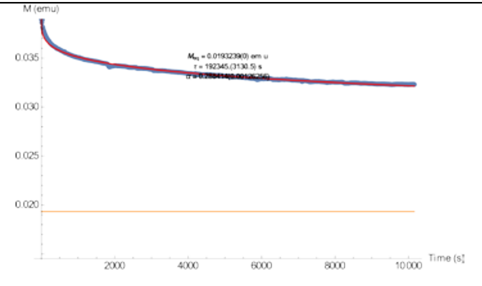
1900	0.023817 [a]		115574	1961	0.266	0.001	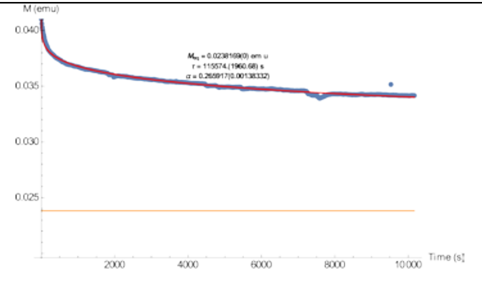
2300	0.027924 [a]		36068	402	0.294	0.002	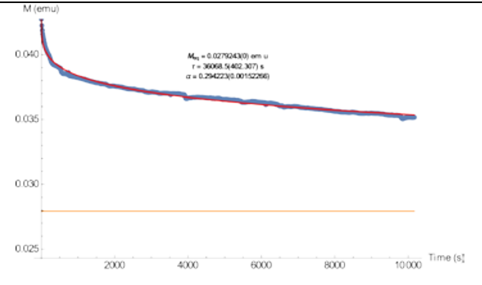
4300	0.035143	0.000294	9013	771	0.440	0.005	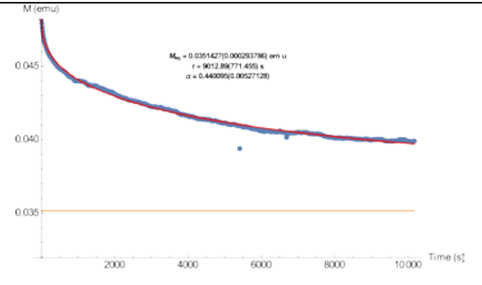
5300	0.039016	0.000277	14515	1223	0.462	0.004	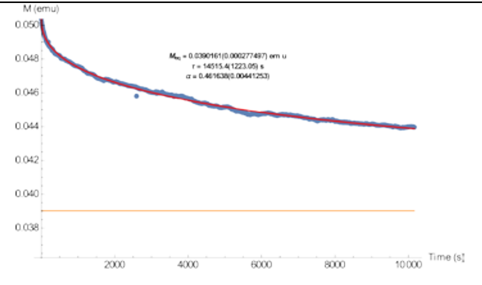
6500	0.048000	0.000032	2080	56	0.524	0.006	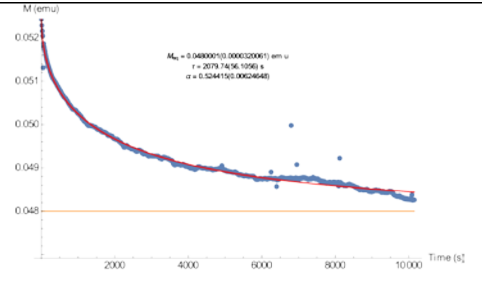

8000	0.051534	0.000020	1822	51	0.492	0.006	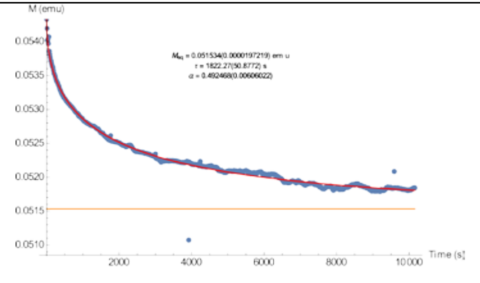
12200	0.056293	0.000005	505	12	0.371	0.006	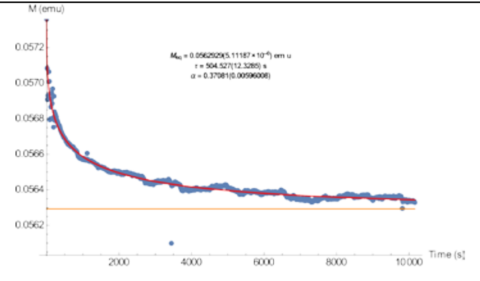

[a] M_{eq} fixed to theoretical value for m_J = ±15/2 doublet scaled to the experimental M_{sat} value at H_i = 50 kOe.

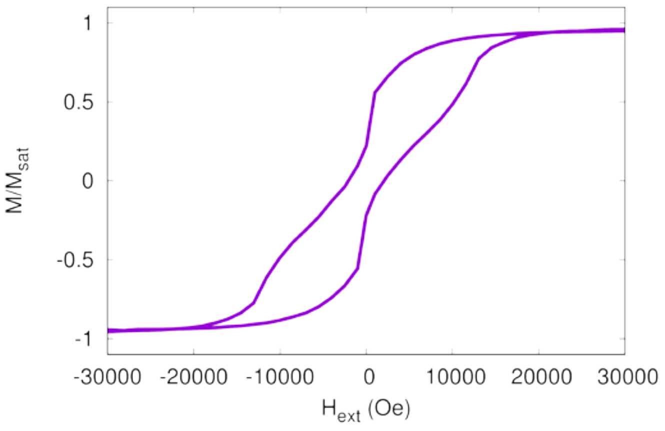

Supplementary Figure 13. Magnetic hysteresis for ~4% ^{164}Dy@**2** at 2 K with a sweep rate of 56(6) Oe s^{-1}.

Magnetic Modelling

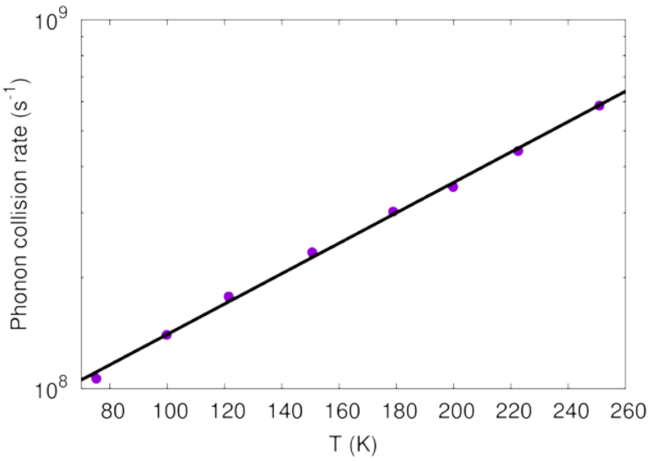

Supplementary Figure 14. Phonon collision rate for silicon (note the log-lin scale). The phonon mean free path has been measured for silicon as a function of temperature[1]. Under the approximation of an isotropic mean speed of sound in the material of *ca.* 7000 m s^{-1} (refs. 2 and 3), we can convert the phonon mean free path to the phonon collision rate with $r = \frac{L}{c}$, where *L* is the phonon mean free path and *c* is the speed of sound in silicon. Points are experimental data for silicon; solid line is best fit to $r = Be^{jT}$ with *B* = 5.44×10^{7} s^{-1} and *j* = 0.00948 K^{-1}.

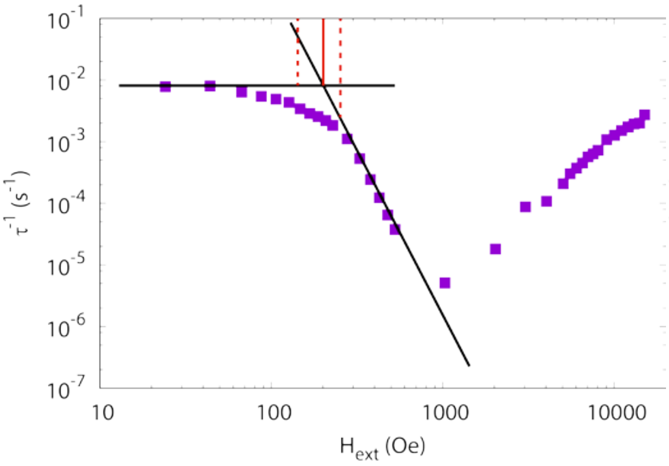

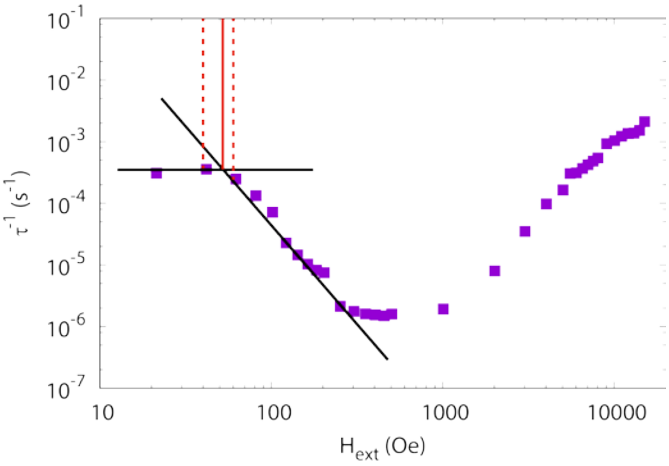

Supplementary Figure 15. left) Magnetic relaxation rate measured by DC magnetometry for **1** at 2 K (note the log-log scale). right) Magnetic relaxation rate measured by DC magnetometry for **1a** at 2 K (note the log-log scale). For both plots: black lines are sketches identifying the field-independent and field-dependent regions in the QTM regime, solid red lines are the intersection between the two regions at approximately $\mu_{\mathbf{1}}$ = 200 and $\mu_{\mathbf{1a}}$ = 50 Oe, and dashed red lines show $\mu \pm \sigma$ for $\sigma_{\mathbf{1}}$ = 50 and $\sigma_{\mathbf{1a}}$ = 10 Oe.

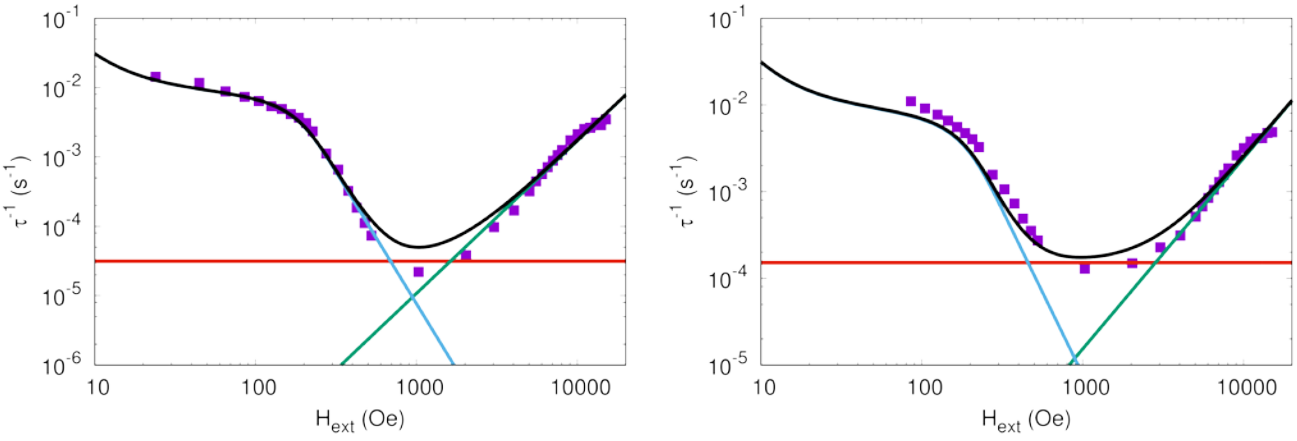

Supplementary Figure 16. left) Magnetic relaxation rate measured by DC magnetometry for **1** at 3 K (note the log-log scale). right) Magnetic relaxation rate measured by DC magnetometry for **1** at 4 K (note the log-log scale). For both plots: black line is a fit with Equation (4) using the parameters in Table 1 in the main text; red line is the Raman component alone, green line is the Direct component alone, blue line is the QTM component alone, Orbach component is not visible on this scale. Error bars are within the data points.

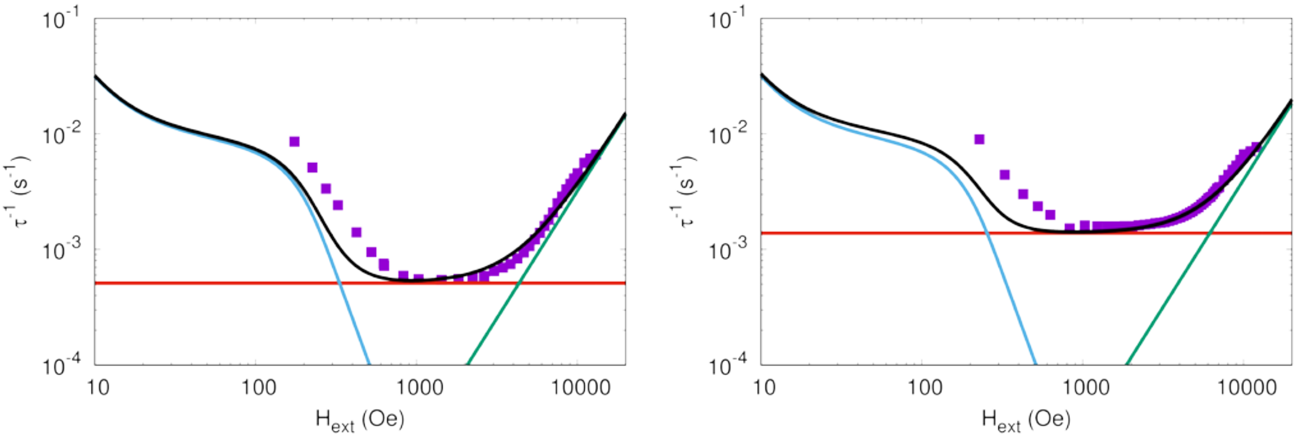

Supplementary Figure 17. left) Magnetic relaxation rate measured by DC magnetometry for **1** at 5 K (note the log-log scale). right) Magnetic relaxation rate measured by DC magnetometry for **1** at 6 K (note the log-log scale). For both plots: black line is a fit with Equation (4) using the parameters in Table 1 in the main text; red line is the Raman component alone, green line is the Direct component alone, blue line is the QTM component alone, Orbach component is not visible on this scale. Error bars are within the data points.

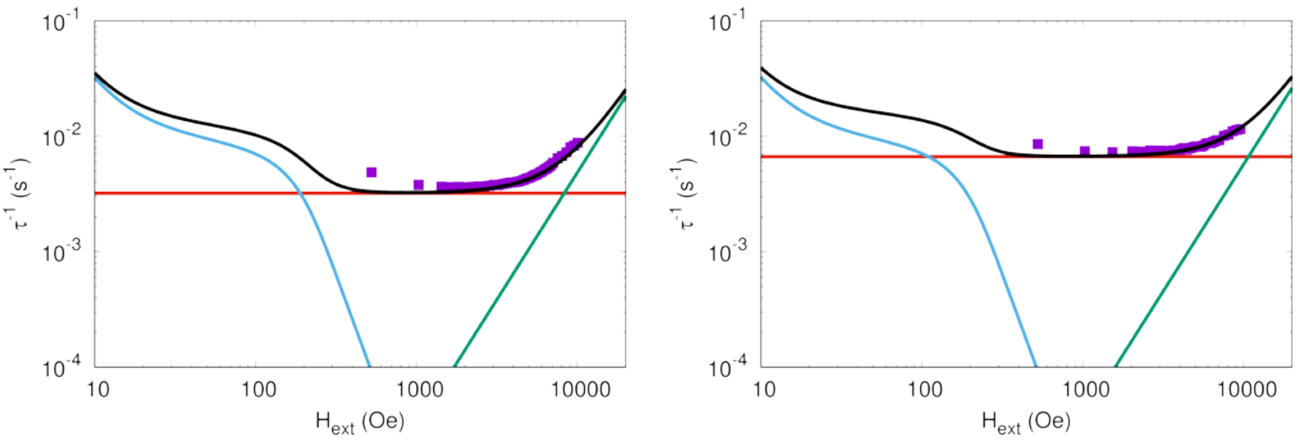

Supplementary Figure 18. left) Magnetic relaxation rate measured by DC magnetometry for **1** at 7 K (note the log-log scale). right) Magnetic relaxation rate measured by DC magnetometry for **1** at 8 K (note the log-log scale). For both plots: black line is a fit with Equation (4) using the parameters in Table 1 in the main text; red line is the Raman component alone, green line is the Direct component alone, blue line is the QTM component alone, Orbach component is not visible on this scale. Error bars are within the data points.

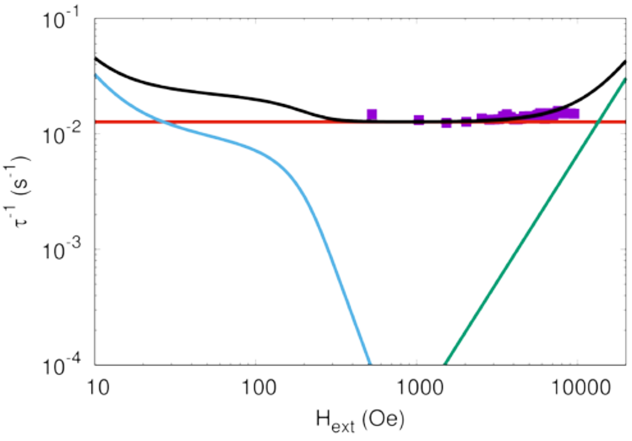

Supplementary Figure 19. Magnetic relaxation rate measured by DC magnetometry for **1** at 9 K (note the log-log scale). Black line is a fit with Equation (4) using the parameters in Table 1 in the main text; red line is the Raman component alone, green line is the Direct component alone, blue line is the QTM component alone, Orbach component is not visible on this scale. Error bars are within the data points.

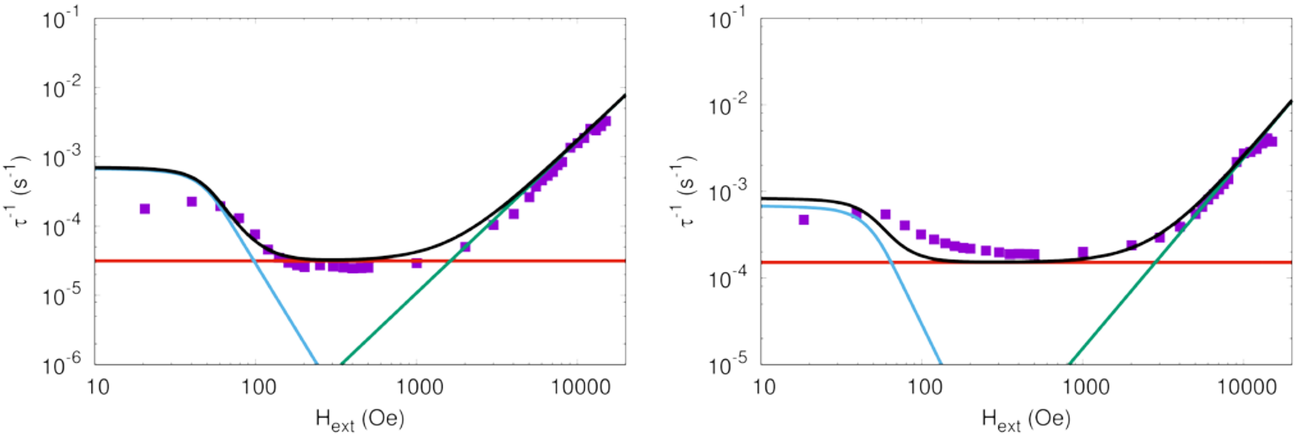

Supplementary Figure 20. left) Magnetic relaxation rate measured by DC magnetometry for **1a** at 3 K (note the log-log scale). right) Magnetic relaxation rate measured by DC magnetometry for **1a** at 4 K (note the log-log scale). For both plots: black line is a fit with Equation (4) using the parameters in Table 1 in the main text; red line is the Raman component alone, green line is the Direct component alone, blue line is the QTM component alone, Orbach component is not visible on this scale. Error bars are within the data points.

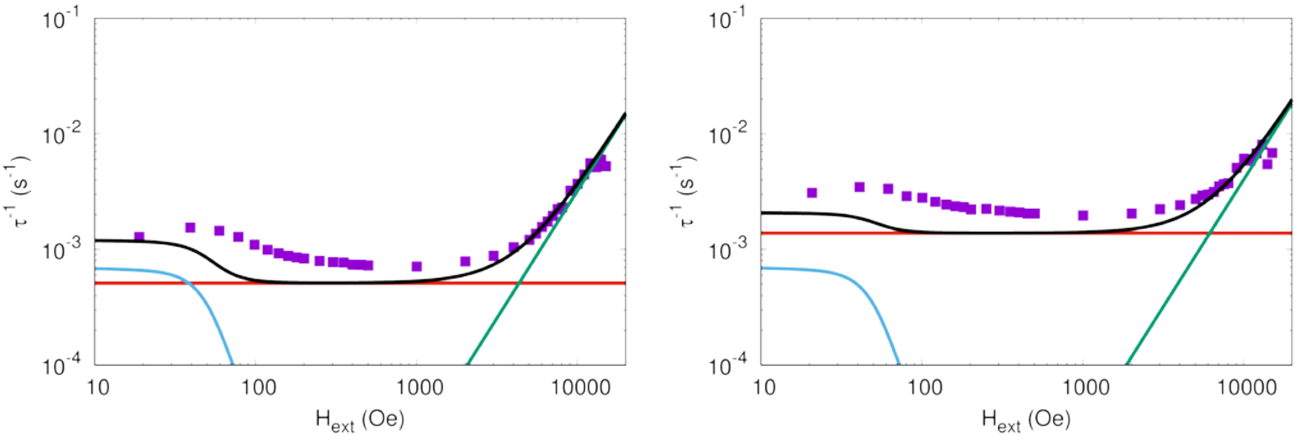

Supplementary Figure 21. left) Magnetic relaxation rate measured by DC magnetometry for **1a** at 5 K (note the log-log scale). right) Magnetic relaxation rate measured by DC magnetometry for **1a** at 6 K (note the log-log scale). For both plots: black line is a fit with Equation (4) using the parameters in Table 1 in the main text; red line is the Raman component alone, green line is the Direct component alone, blue line is the QTM component alone, Orbach component is not visible on this scale. Error bars are within the data points.

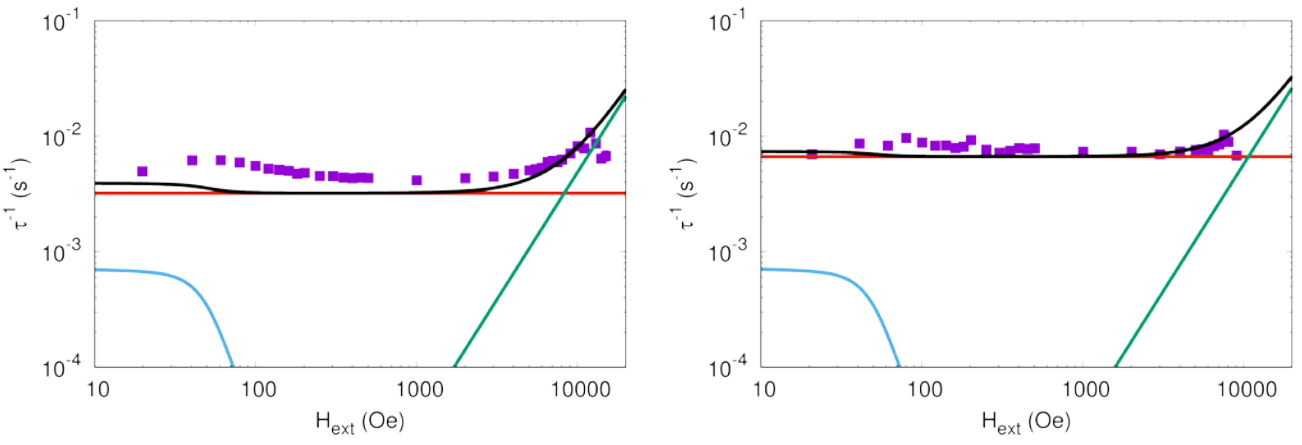

Supplementary Figure 22. left) Magnetic relaxation rate measured by DC magnetometry for **1a** at 7 K (note the log-log scale). right) Magnetic relaxation rate measured by DC magnetometry for **1a** at 8 K (note the log-log scale). For both plots: black line is a fit with Equation (4) using the parameters in Table 1 in the main text; red line is the Raman component alone, green line is the Direct component alone, blue line is the QTM component alone, Orbach component is not visible on this scale. Error bars are within the data points.

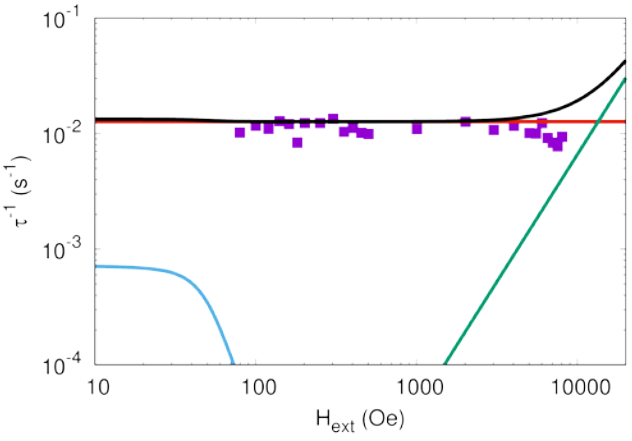

Supplementary Figure 23. Magnetic relaxation rate measured by DC magnetometry for **1a** at 9 K (note the log-log scale). Black line is a fit with Equation (4) using the parameters in Table 1 in the main text; red line is the Raman component alone, green line is the Direct component alone, blue line is the QTM component alone, Orbach component is not visible on this scale. Error bars are within the data points.

Supplementary References

1. Gereth, R. & Hubner, K. Phonon mean free path in silicon between 77 and 250 K. *Phys. Rev.* **134,** A235 (1964).
2. Hall, J. J. Electronic effects in the elastic constants of n-type silicon. *Phys. Rev.* **161,** 756 (1967).
3. Kinsler, L. E. *Fundamentals of acoustics*. (Wiley, 2000).
