# Supplementary material for: Field- and temperature-dependent quantum tunnelling of the magnetisation in a large barrier single-molecule magnet
Source: Nat Commun. 2018 Aug 7;9:3134. doi: 10.1038/s41467-018-05587-6 (PMC6081483; doi:10.1038/s41467-018-05587-6)
Supplement: Supplementary file 2 — Peer Review File [file 41467_2018_5587_MOESM2_ESM.pdf]

Editorial Note: this manuscript has been previously reviewed at another journal that is not operating a transparent peer review scheme. This document only contains reviewer comments and rebuttal letters for versions considered at Nature Communications.

REVIEWERS' COMMENTS:

Reviewer #1 (Remarks to the Author):

The authors have now addressed satisfyingly my questions from the last iteration, in particular by addition of the 164Dy experiment,I recommend publication of the ms as it is. No additional revision is needed anymore.
